# MEDICC2: whole-genome doubling aware copy-number phylogenies for cancer evolution

**DOI:** 10.1186/s13059-022-02794-9

**Published:** 2022-11-14

**Authors:** Tom L. Kaufmann, Marina Petkovic, Thomas B. K. Watkins, Emma C. Colliver, Sofya Laskina, Nisha Thapa, Darlan C. Minussi, Nicholas Navin, Charles Swanton, Peter Van Loo, Kerstin Haase, Maxime Tarabichi, Roland F. Schwarz

**Affiliations:** 1 Berlin Institute for Medical Systems Biology, Max Delbrück Center for Molecular Medicine in the Helmholtz Association (MDC), Robert-Rössle-Str. 10, 13125 Berlin, Germany; 2 Department of Electrical Engineering & Computer Science, Technische Universität Berlin, Marchstr. 23, 10587 Berlin, Germany; 3 BIFOLD, Berlin Institute for the Foundations of Learning and Data, Berlin, Germany; 4 Department of Biology, Humboldt University of Berlin, Unter den Linden 6, 10099 Berlin, Germany; 5 Division of Oncology and Hematology, Department of Pediatrics, Charité – Universitätsmedizin Berlin, corporate member of Freie Universität Berlin and Humboldt Universität zu Berlin, Augustenburger Platz 1, 13353 Berlin, Germany; 6 The Francis Crick Institute, London, UK; 7 Department of Mathematics and Computer Science, Free University of Berlin, Berlin, Germany; 8 UCL Medical School, University College London, London, UK; 9 Department of Genetics, The University of Texas MD Anderson Cancer Center, Houston, TX USA; 10 Cancer Research UK Lung Cancer Centre of Excellence, University College London Cancer Institute, London, UK; 11 Department of Medical Oncology, University College London Hospitals, London, UK; 12 Department of Genomic Medicine, The University of Texas MD Anderson Cancer Center, Houston, TX USA; 13 German Cancer Consortium (DKTK), German Cancer Research Center (DKFZ), Heidelberg, Germany; 14 Institute for Interdisciplinary Research, Université Libre de Bruxelles, Brussels, Belgium; 15 Institute for Computational Cancer Biology, Center for Integrated Oncology (CIO) and Cancer Research Center Cologne Essen (CCCE), Faculty of Medicine and University Hospital Cologne, University of Cologne, Cologne, Germany

**Keywords:** Somatic copy-number alterations, Chromosomal instability, Aneuploidy, Whole-genome doubling, Intratumor heterogeneity, Cancer evolution, Phylogenetic reconstruction, Single-cell sequencing

## Abstract

Aneuploidy, chromosomal instability, somatic copy-number alterations, and whole-genome doubling (WGD) play key roles in cancer evolution and provide information for the complex task of phylogenetic inference. We present MEDICC2, a method for inferring evolutionary trees and WGD using haplotype-specific somatic copy-number alterations from single-cell or bulk data. MEDICC2 eschews simplifications such as the infinite sites assumption, allowing multiple mutations and parallel evolution, and does not treat adjacent loci as independent, allowing overlapping copy-number events. Using simulations and multiple data types from 2780 tumors, we use MEDICC2 to demonstrate accurate inference of phylogenies, clonal and subclonal WGD, and ancestral copy-number states.

**Supplementary Information:**

The online version contains supplementary material available at 10.1186/s13059-022-02794-9.

## Background

Somatic copy-number alterations (SCNAs) and chromosomal instability (CIN) are hallmarks of many tumors and drive genome plasticity and intratumor heterogeneity (ITH) [1, 2]. SCNAs are subject to continuous evolution and selection across cancer types [3], and haplotype-resolved SCNA analyses have revealed parallel and potentially convergent evolution, including mirrored subclonal allelic imbalance (MSAI) events [4]. Besides their clinical relevance [5], SCNAs are a rich source of genetic variation that can be leveraged to reconstruct tumor evolution [6, 7]. However, for evolutionary reconstructions, SCNAs pose particular challenges, including statistical dependencies between genomic loci, overlapping of individual gain/loss events causing backmutations and physical constraints, e.g., that fully deleted genetic material cannot be regained at a later time point [6, 8, 9]. These characteristics of SCNA events necessitate an explicit evolutionary model of individual haplotype-specific copy-number changes to allow for accurate phylogenetic reconstructions.

Such an evolutionary model should also include whole-genome doubling (WGD) events [10–13], which have long been known to be linked to tumorigenesis [14–19], and which have been identified as key contributors to CIN [3, 11, 20, 21] and as potential therapeutic targets [22–24]. WGD involves tetraploidization of genomes frequently followed by immediate loss of individual chromosomes [12, 20], thus buffering cancer genomes against the accumulation of deleterious mutations [21] and forming a substrate for further genomic diversification [3, 21]. Statistical indicators of WGD include a high average ploidy [12] in relation to the frequency of loss-of-heterozygosity (LOH) events in a cohort [25], or evidence from the clone structure of multiple samples [18]. From an evolutionary perspective, reliably detecting WGD events requires weighing a complete doubling of the genome followed by chromosomal losses against successive gains of individual chromosomes.

While several SCNA-based evolutionary inference methods have been proposed in the past [26–29], they do not model WGD events, frequently make use of the infinite sites assumption [30] and thus cannot infer parallel evolution, and do not deal with statistical dependencies between genomic loci. They are further often restricted to solving the much simpler problem of tree inference with fully sampled data, i.e., where the ancestral (internal) nodes of the tree are accessible through sequencing, an unrealistic assumption in most cases. Alternatively, other studies [31, 32] use hierarchical clustering based on, e.g., Euclidean or Hamming distances, which are not based on evolutionary principles, to infer trees from SCNAs and interpret them as phylogenies of cancer genomes.

To address this, we have developed MEDICC2 to infer phylogenies from SCNAs based on the minimum-event distance (MED) [6, 33], i.e., the minimum number of evolutionary events (including LOH, WGD, and segmental gains and losses of arbitrary size) needed to transform one genome into another. MEDICC2 computes the MED including WGD events in linear time, reconstructs phylogenetic trees in the presence of homoplasy, infers ancestral genomes, and times SCNA events including WGD relative to each other. We apply MEDICC2 to 2778 tumors from the Pan-Cancer Analysis of Whole Genomes (PCAWG), where it accurately identifies WGD against a “gold standard” set of WGD calls determined using consensus copy-number profiles from six copy-number callers [25, 34]. Using multi-sample prostate cancer cases, we demonstrate MEDICC2’s ability to detect subclonal WGD events and to correctly place parallel evolution and MSAI events revealed by multi-sample phasing [3, 4]. We use orthogonally derived structural variant (SV) data from the same cohorts to validate the evolutionary events inferred by MEDICC2 and ultimately show how MEDICC2 infers phylogenies from allele-specific copy-number profiles for thousands of single cells without prior clustering or data aggregation.

## Results

### Inferring phylogenies from SCNAs with MEDICC2

**Fig. 1:**
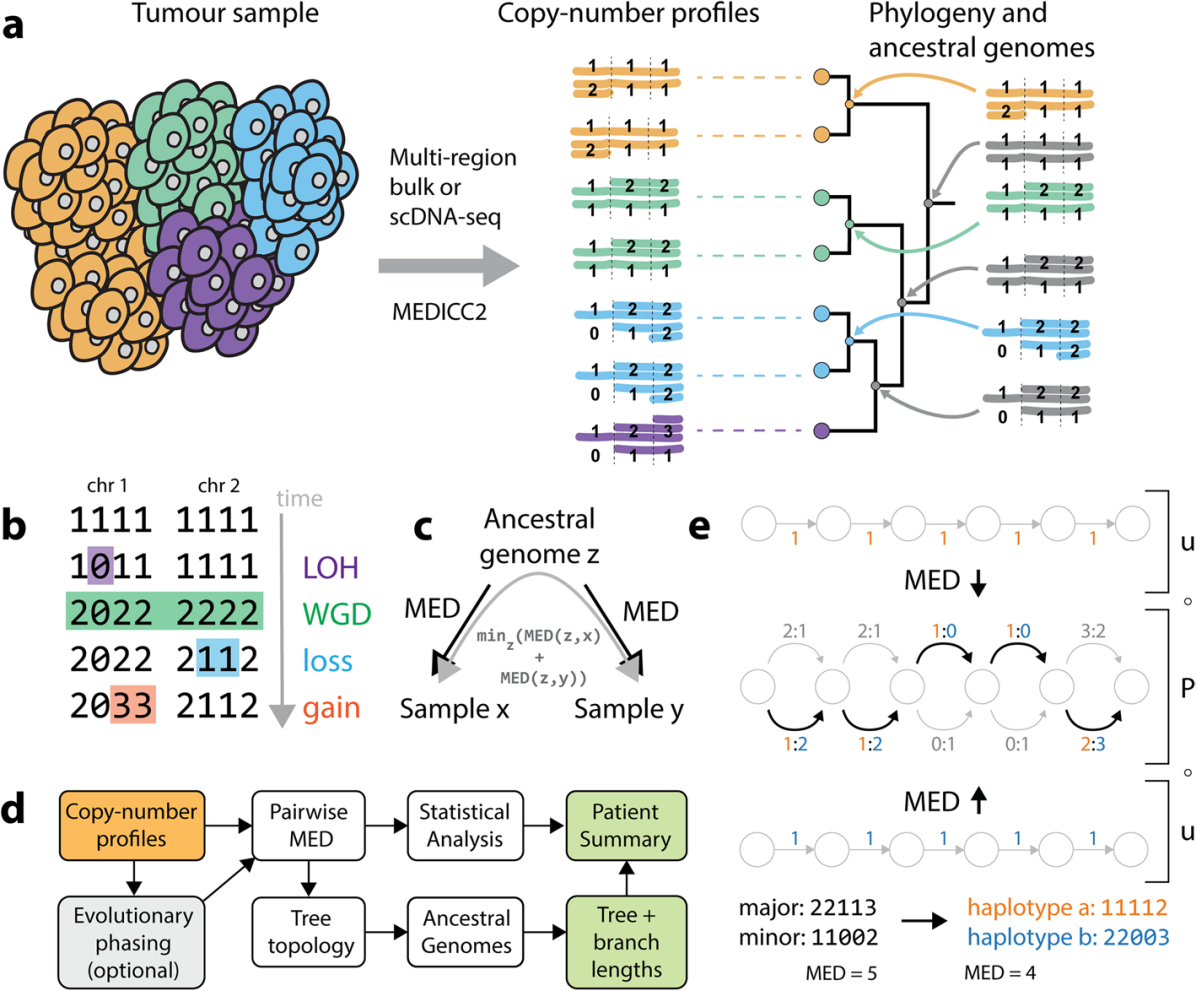
MEDICC2 algorithm. **a** MEDICC2 infers cancer phylogenies from SCNA data from single cells or bulk sequencing using a minimum-event distance (MED) and infers the ancestral genomes. It allows for backmutations, obeys biological constraints, and solves the phylogeny problem where ancestral genomes are not sampled. **b** Computing distances with WGD. Copy-number profiles are represented as vectors of positive integer copy numbers across chromosomes (here: two chromosomes with four segments each). To infer the correct MED, LOH events are considered first as lost segments cannot be re-gained by later events. WGD events span the full copy-number profile, whereas gain and loss events can affect an arbitrary number of segments within a chromosome. **c** Symmetric distance calculation. The MED from an ancestral state to a sample is asymmetric due to biological constraints. The final symmetric distance between two samples is computed as the sum of distances from an ancestral genome to both samples, while minimizing over all possible ancestors. **d** Schematic overview of the MEDICC2 workflow. Haplotype-specific copy-number profiles are either pre-phased or undergo evolutionary phasing (see **e**). Pairwise MEDs are computed between all genomes, followed by tree inference and ancestral reconstruction which determines the final branch lengths of the tree. Results are reported to the user as a patient summary and plot. **e** Evolutionary phasing. Copy-number profiles for both alleles are jointly encoded as an unweighted phasing FST P where both possible allele configurations are encoded at each position in the sequence. Evolutionary phasing then determines the optimal configuration (bold arrows) and extracts final haplotypes (orange and blue) by computing the MED between the phasing FST and two reference haplotypes. An example of major/minor copy number, phased copy number, and the MED from the diploid is shown at the bottom. Abbreviations: FST: Finite-state transducer, MED: Minimum-event distance, LOH: Loss-of-heterozygosity, WGD: Whole-genome doubling

MEDICC2 infers phylogenies and ancestral genomes from SCNAs (Fig. 1a) by solving the MED problem, originally formulated by us [6] and recently studied by Zeira et al. [33], using a weighted finite-state transducer (FST) framework [35]. Briefly, the MED between a pair of copy-number profiles is defined as the minimum number of gains and losses of arbitrary length needed to transform one copy-number profile into another (“Methods”). MEDICC2 thereby enforces physical constraints where gains of zero-copy segments are not permitted and zero-copy segments are “ignored” by subsequent operations, mimicking the absence of that segment of genomic DNA (Fig. 1b, Additional file 1: Fig. S1). This MED is thus asymmetric, and the symmetric distance between a pair of copy-number profiles is computed by minimizing the MED between two copy-number profiles and their evolutionary ancestor [6] (Fig. 1c). For this, the FST implementing the MED has to be composed with its inverse, a complex operation. To avoid constructing this explicitly, we here employ a new lazy composition strategy, which only expands the FST along the path required for shortest-path computation (“Methods”).

To model WGD events (MED-WGD), MEDICC2 processes whole-genome copy-number profiles including both haplotypes at once, while keeping track of chromosome boundaries. Standard gain and loss events terminate when they reach the end of a chromosome. WGD events are gains applied to all non-zero segments in the genome (thereby doubling both haplotypes) irrespective of chromosome boundaries. Tetraploidization followed by rapid chromosomal loss to reach a near-triploid state [12, 20] has been described in many tumor types and is naturally contained in our model in the form of a WGD event followed by multiple losses of individual chromosomes.

Before calculating distances, copy-number profiles are typically phased (Fig. 1d,e), either through the use of multi-sample reference phasing using Refphase [3], or through an internal evolutionary phasing routine (“Methods”), which chooses a haplotype configuration that minimizes the total MED between the genome and a reference genome, typically a diploid normal (Fig. 1e). MEDICC2 then infers the tree topology from pairwise MEDs between all genomes using neighbor joining [36] and calculates summary statistics as previously described [6]. Finally, ancestral copy-number profiles are reconstructed such that the total number of events along the tree is minimal, which determines the final branch lengths of the tree. The result is reported to the user as a patient summary and plot which includes the tree and inferred ancestral and terminal copy-number profiles, and change events, either globally for the whole genome or at user-defined positions of interest, e.g., at oncogenes and tumor suppressor genes.

**Fig. 2:**
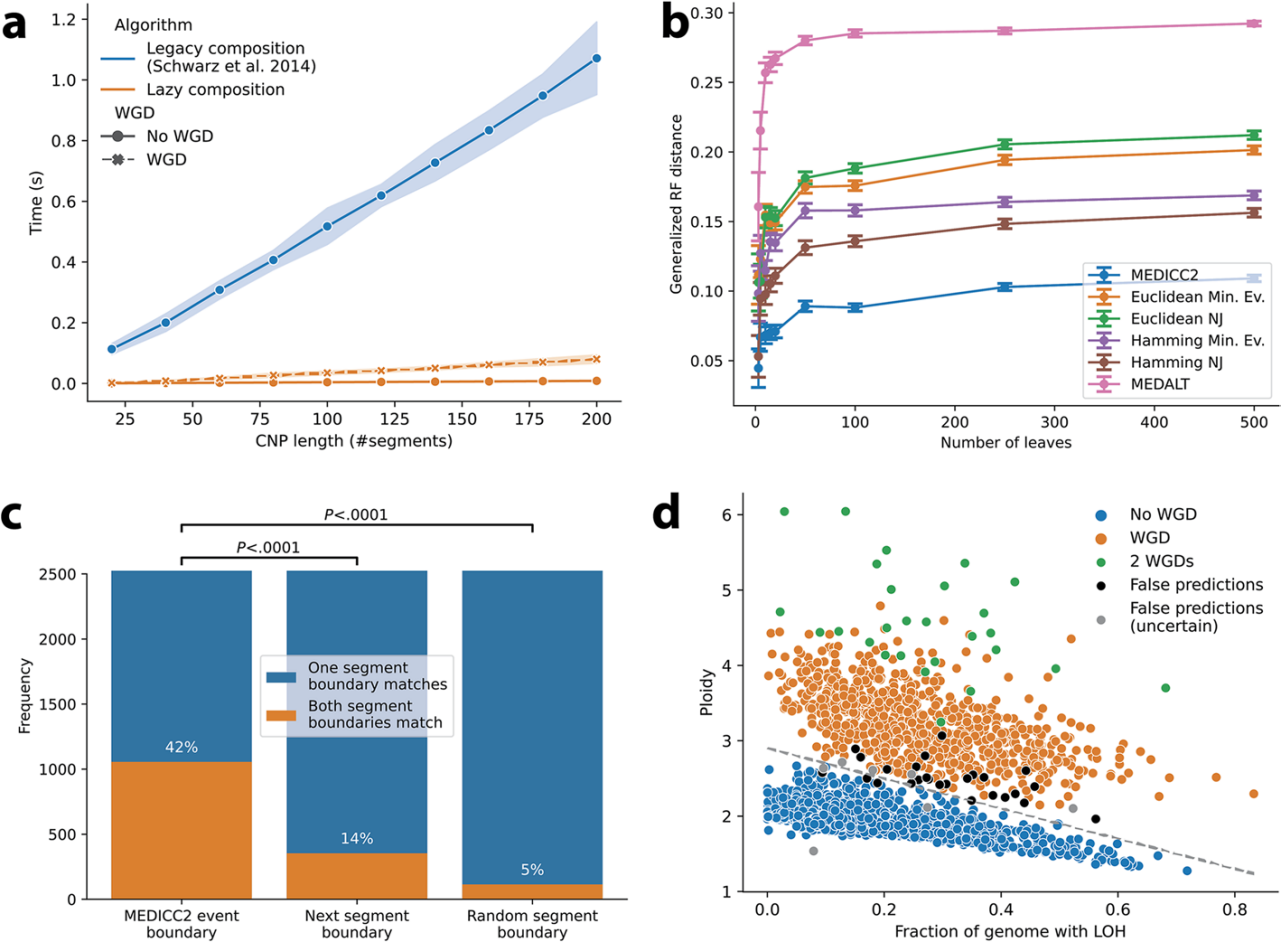
Algorithm performance and validation. **a** Runtime of different composition strategies. Copy-number profiles were simulated with increasing lengths from 20 to 200 segments. Computation time of the MEDs is linear with respect to the length of the input sequences. While MED-WGD took significantly longer to compute than the MED without WGD, the new lazy composition strategy reduced runtime by orders of magnitude. Shaded areas correspond to standard errors. **b** Performance on simulated data: Using an independent simulation routine we benchmarked MEDICC2 against a range of other methods. The reconstructed trees were compared to the simulated trees using the generalized Robinson-Foulds distance. As expected, the GRF distance rises with increasing tree size. MEDICC2 outperforms all other methods for all tree sizes. **c** Validation of MEDICC2 events with SVs. Pairs of MEDICC2 events and SVs were chosen based on an overlap of the starting segment. We assume MEDICC2 events to be supported by the SV if the ends also overlap. Shown here are the results using only duplications and deletions with size larger than 10Mbp. **d** The MEDICC2 WGD score for 2778 cancer genomes. Individual cancers are plotted based on their average ploidy and fraction of genome with LOH. The original separating line between WGD and non-WGD tumors was estimated by Dentro et al. as *y* = 2.9 − 2*x*. Correct “WGD” and “no WGD” predictions from MEDICC2 were marked in orange and blue while false predictions were marked in black and gray (latter if the PCAWG WGD status was “uncertain”). Abbreviations: NJ: Neighbor joining, Min. Ev.: Minimum Evolution

We first verified the technical accuracy and time complexity of the MED inference by simulating copy-number profiles with a known distance from a diploid normal under the MEDICC2 model. MEDICC2 correctly estimated the MED in linear time (Fig. 2a), and the inferred MED forms a lower bound to the true number of events (minimum event criterion) with and without WGD in contrast to Euclidean distance (*r*^2^=0.17, Additional file 1: Fig. S2). The new lazy composition strategy leads to a performance increase of about one order of magnitude, enabling distance calculations for a large number of samples or single cells.

We next assessed the tree reconstruction accuracy of MEDICC2 in comparison to alternative inference tools through simulations. To not bias the results, evolution was simulated at the level of the genome through chromosomal and segmental gains and losses but also copy-number neutral events including inversions and balanced translocations and complex events such as breakage-fusion-bridges and WGDs (“Methods”). From these simulated genomes with varying mutation rates, copy-number profiles were generated by counting the number of copies of each segment. The profiles were then subjected to different tree reconstruction strategies, including Euclidean and Manhattan distances with the neighbor joining [36] and minimum evolution [37] algorithms, as well as the recently developed tools MEDALT [28] and Sitka [38]. MEDICC2 outperforms other methods for all ranges of mutation rates and tree sizes, especially in the presence of WGDs and independent of the tree metric used (Fig. 2b, Additional file 1: Fig. S3).

As MEDICC2 makes the assumption that segments are contiguous with respect to the reference genome, we wanted to test its performance in the face of violations of this assumption. Therefore, we used our simulation routine to create datasets with increased numbers of translocations and inversions (which alter the order of segments with respect to the reference genome but do not change copy number). While the introduction of translocation and inversion events lead to a slight decrease in reconstruction accuracy, MEDICC2 proved largely robust to violations of the contiguity assumption and still outperformed all other methods (Additional file 1: Fig. S4).

We next assessed the computation time of MEDICC2 systematically and compared runtimes to those of Sitka and MEDALT on simulated trees. While the MED computation between two samples in MEDICC2 is linear in the number of genomic segments, pairwise MED calculation between all samples makes the overall algorithmic complexity quadratic in the number of samples. Overall, we found MEDICC2 to be slower than Sitka and MEDALT, but due to a new efficient parallelization strategy (“Methods” and [39]) together with the improved performance of the MED computation, runtime remained manageable even for up to 1000 samples (Additional file 1: Fig. S5 and Table S1).

### MEDICC2 identifies individual genomic events that change copy number and accurately detects WGD in 2778 cancers

MEDICC2 models individual genomic events that change copy number and that can span multiple segments in the input copy-number profiles. To investigate whether predicted MEDICC2 events accurately describe genome evolution, we compared the detected event boundaries in 2778 tumors from the Pan-cancer Analysis of Whole Genomes (PCAWG) [34] to orthogonal SV data from the same cohort. In addition to high-fidelity copy-number profiles and SV data, PCAWG provides reliable annotation of each tumor’s WGD status, which serves us as a “gold standard” for evaluating MEDICC2’s WGD detection performance.

We first extracted all copy-number events between a diploid normal and each of the 2778 PCAWG genomes. To compare the extracted copy-number events to SVs, we selected all MEDICC2 events where one of its event boundaries (start or end) overlaps with an SV breakpoint (“Methods,” Additional file 1: Fig. S6). We then counted how often the second SV breakpoint overlaps with (i) the second MEDICC2 event boundary, (ii) the next copy-number segment boundary (with respect to the first breakpoint), or (iii) a random copy-number segment boundary on the same chromosome (“Methods,” Additional file 1: Fig. S6). MEDICC2 events more frequently agree with SV breakpoints than the copy-number segments or random segment boundaries (Fig. 2c) irrespective of the size or type of SV considered (Additional file 1: Fig. S7). We find that the mismatched MEDICC2 breakpoints are close to the corresponding SV breakpoints and vice versa (Additional file 1: Fig. S8, S9). These findings confirm that MEDICC2 events more accurately describe genome evolution than measures based on copy-number segments alone.

Next, we tested MEDICC2’s ability to detect WGDs by comparing our results to the WGD status in PCAWG, which was inferred from the relationship between tumor ploidy and the percentage of the genome affected by LOH across the cohort [25] (“Methods”) with 818 samples labelled as WGD positive and 1960 as WGD negative. MEDICC2 correctly predicted the WGD status of 2668 out of the 2778 cases (96.0%), 12 of which were predicted to have undergone two consecutive WGD events. All of the 110 incorrect predictions were false negatives, i.e., they were labelled as WGD in PCAWG but not called by MEDICC2. Since PCAWG WGD annotations are also based on biological data with inherent noise and may contain errors, we investigated whether the 110 missed cases of WGD were marked as “WGD uncertain” by the PCAWG heterogeneity and evolution working group. Indeed, tumors with status “WGD uncertain” were significantly overrepresented among these tumors (17 out of the 110 incorrect MEDDIC2 predictions, *P* = 1.2 · 10^−8^, chi-square test). To increase sensitivity and in order to mitigate the effect of noisy data, we created 100 bootstrap replicates for each sample (“Methods”) and calculated the WGD evidence scores for each replicate. Marking samples as WGD if at least 5% of their bootstrap runs exhibited at least one WGD event increased the detection accuracy of WGDs to 98.8% (33 incorrect predictions: 6 false positives and 27 false negatives) (Fig. 2d, Additional file 1: Fig. S10) while maintaining an over-representation of false predictions among tumors with status “WGD uncertain” (7 out of the 33 incorrect MEDDIC2 predictions, *P* = 9.6 · 10^−6^, chi-square test). Bootstrap sampling identified an additional 15 samples (total of 27) that underwent two successive WGDs (Fig. 2d). We found the WGD detection accuracy to be largely independent of the bootstrap percentage threshold chosen (area under the precision-recall curve AUC=0.99, Additional file 1: Fig. S11).

These results demonstrate that MEDICC2 accurately reconstructs individual evolutionary events and infers the presence of WGD events even in single-sample studies, without the need for additional parameter estimation or cohort-level statistics. If required, bootstrap resampling can be used to increase sensitivity and resilience against noise.

### MEDICC2 reveals subclonal WGD events and parallel evolution in prostate cancer

We next reconstructed phylogenies and inferred ancestral genomes for a multi-sample, whole-genome sequencing (WGS) cohort with 10 metastatic prostate cancer patients introduced in Gundem et al. [40]. MEDICC2 inference took less than 2 min each on a desktop computer (Additional file 1: Table S2).

We first compared the MEDICC2 phylogenies based on copy-number profiles of the dominant subclone (derived with the Battenberg algorithm, “Methods”) in each sample to the SNV-based clone phylogenies produced by Gundem et al. Despite the differing resolution of the two approaches, we observed exact concordance, defined as Robinson-Foulds distance of zero, between the SCNA-based MEDICC2 phylogenies and the SNV-based clone phylogenies in 6 out of 10 tumors (A10, A12, A21, A29, A31, and A34) and partial concordance in the remaining 4 tumors (Robinson-Foulds distances: A17:3, A22:7, A24:2, and A32:2) (“Methods,” Additional file 1: Fig. S12-S20). Notably, using SNV cancer cell fraction information, metastatic samples from A22, A24, and A32 were classified as demonstrating polyclonal seeding with multiple subclones present within individual samples while A17 was classified as showing inconclusive evidence of such polyclonal seeding [40]. The copy-number profiles of these multiple subclones within a single sample will not be captured by querying only the dominant subclone within that sample (“Methods”) and therefore this may contribute to the partial concordance observed between the MEDICC2 and SNV-based phylogenies in these tumors.

**Fig. 3:**
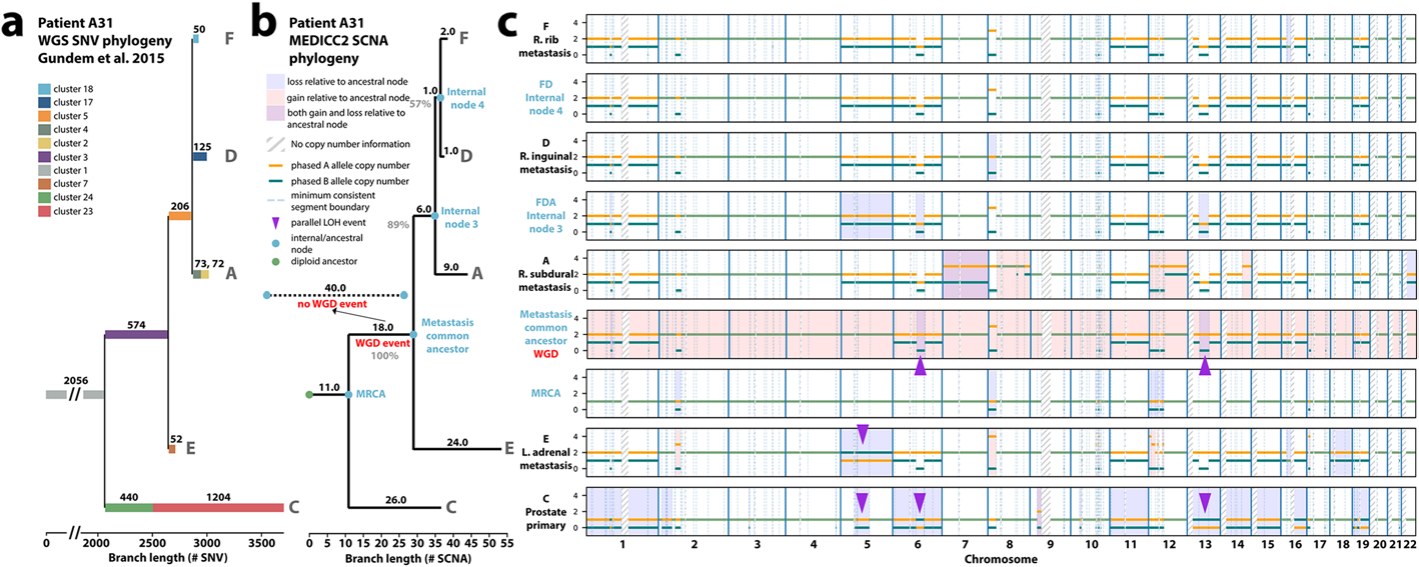
Evolutionary history of tumor subclones from patient A31. **a** SNV-based phylogeny. Reproduction of the SNV-based phylogeny as described in Gundem et al. [40] for the multi-sample prostate cancer tumor case with one sample (C) from the primary tumor and four samples (A, D, E, and F) from distinct metastatic sites. Original reconstruction was performed using an n-dimensional Bayesian Dirichlet process to cluster estimated cancer cell fractions of the single-nucleotide variants (SNV) identified in the WGS across samples. Only the major subclone of each sample is shown (“Methods”). **b** MEDICC2 phylogeny. Using multi-sample phased copy-number profiles, MEDICC2 detected the presence of WGD in the metastatic samples and its absence in the primary sample from A31. The MEDICC2 analysis identifies multiple MSAI events as well as parallel LOH on 6 and 13 (purple arrows). Individual events are marked in the copy-number track where they occur: gains (red) and losses (blue). The gray number in each branch corresponds to its bootstrap-confidence score while the WGD events from the MEDICC2 event detection are marked in green

**Fig. 4:**
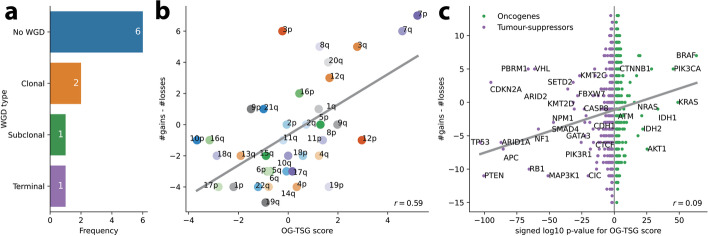
Event detection for the Gundem et al. [40] cohort. **a** WGD detection. In the 10 patients, a total of 4 WGDs were detected, two of which were clonal, one subclonal and one in a terminal branch. **b** Distribution of arm-level events. Using the MEDICC2 event detection routine, we detected the number of times a whole chromosome arm was either gained or lost in a single branch. The gains and losses were aggregated over all patients and samples into a single score. This score was compared against the oncogene - tumor suppressor gene (OG-TSG) score derived by Davoli et al. [41]. A clear correlation between the gains/losses and the OG-TSG score (which is not based on copy numbers) is visible. **c** Distribution of gene-level events. The analysis was repeated on the basis of all 1729 individual genes present in the Davoli et al. dataset. On the *x*-axis, we plotted the base-10 logarithm of the genes’ *p*-values and flipped the sign for the oncogenes to create a single, continuous *x*-axis for both genesets. A small correlation is visible which becomes more pronounced when only considering the top 100 genes. Names are given for genes with *p* < 10^−20^

An illustrative example of a comparison between MEDICC2 and the original SNV-based phylogenetic reconstructions is that of A31 (Fig. 3), which consists of sample C from the primary tumor and four samples (A, D, E, and F) from distinct metastatic sites. A31 was later analyzed as part of the PCAWG cohort [25] and found to demonstrate a subclonal WGD event affecting all metastatic samples but not the primary sample C. In addition to faithfully recovering the original phylogeny, MEDICC2’s ancestral reconstruction correctly detected and placed the WGD event at the ancestor of the metastatic samples (WGD evidence score *s*_*A*31_ = 22, Fig. 3b). In the 10 patients, four WGD events were identified, two of which were clonal and two were subclonal with both subclonal WGD events occurring in metastatic samples (Fig. 4a). In A31, this subclonal WGD was followed by a gain on chromosome 8p and multiple chromosome-wide losses. The most recent common ancestor (MRCA) of all A31’s samples however revealed only moderate SCNA burden with clonal LOH on chromosomes 2, 6, 12, and 17, indicative of the substantial divergence between the primary tumor and the metastases. Finally, the ancestor of the three metastatic samples A, D, and F revealed an MSAI loss on chromosome 5 different from metastasis sample E.

For validation, we repeated the comparison of MEDICC2 events with SV data for all Gundem trees. Events that were assigned to ancestral nodes were compared to SVs present in all child samples. Despite the smaller size of the dataset (50 samples) compared to the single-sample PCAWG cohort, MEDICC2 events agree with the SV data (Additional file 1: Fig. S21).

We next compared the branch lengths of the SNV-based trees and the SCNA-based MEDICC2 trees, where branch lengths correspond to the number of SNVs and to the number of SCNA events larger than 1 Mb respectively. We first investigated the relationship between the roof-to-leaf distances in the SNV and SCNA trees globally across the cohort and observed a significant correlation between SNV and SCNA root-to-leaf lengths (*ρ* = 0.57, *P* = 1.6 × 10^−5^, Spearman correlation, Additional file 1: Fig. S22).

However, the SNV-based and SCNA-based trees demonstrated distinctly different lengths from the root (diploid normal) to the MRCA, relative to the maximal root-to-leaf distance of the tree. For example, in A31, this “trunk” was found to be shorter in the SCNA-based MEDICC2 tree with 11/54 SCNA events (11 SCNA events relative to 54 SCNA events on the longest root-to-leaf distance in the MEDICC2 tree) when compared to 2056/3700 SNVs (2056 SNVs relative to 3700 SNVs on the longest root-to-leaf distance in the SNV tree). This suggests that there have been relatively few founder SCNAs compared to a large number of founder SNVs, potentially due to a larger number of SNVs present in the tissue before malignant transformation. This finding was replicated in 9/10 of the prostate tumors with A34 being the exception (Additional file 1: Fig. S12-S20).

In A31, the root-to-leaf distances of the SNV-based and MEDICC2 trees were relatively similar to one another for samples A (SNV: 2981/3700 vs MEDICC2: 41/54), F (SNV: 2886/3700 vs MEDICC2: 35/54), and D (SNV: 2961/3700 vs MEDICC2: 34/54). However, the root-to-leaf distances of the two methods were more different for branches E and C. The branch terminating at the dominant clone of metastatic sample E was relatively long in the MEDICC2 tree compared to the SNV tree (SNV: 2682/3700 vs MEDICC2: 54/54) (Fig. 3b). This is due to multiple SCNAs affecting chromosome 12 in sample E which may suggest the presence of a complex event resulting in the co-occurrence of these SCNAs. In contrast, the root-to-leaf distance of sample C from the primary tumor in the SNV-based tree is relatively long compared to the MEDICC2 tree (SNV: 3700/3700 vs MEDICC2: 31/54). This branch is the only one to demonstrate additional substitution-based driver mutations affecting *TP53* and *KMT2C* beyond the drivers present clonally in the trunk. In this tumor, the metastatic samples are derived from a minor subclone in the primary that underwent WGD and developed additional structural variant driver mutations, potentially reflecting the previously reported links between increased ploidy and structural variation with metastasis [42, 43].

Recently, we developed a multi-sample reference phasing algorithm that maintains consistent phased haplotypes across samples from a single patient’s disease to reveal additional SCNA heterogeneity across human cancers [3, 4]. This additional heterogeneity results from the detection of MSAI as well as SCNA-mediated parallel evolution where the same SCNA event (e.g., an LOH event) occurs independently affecting distinct haplotypes within an individual patient’s disease [3, 4, 44, 45]. Since MEDICC2 models both haplotypes individually and does not employ the infinite site assumption, it can infer both MSAI-mediated homoplasy and homoplasy affecting the same allele by assigning these parallel events to separate branches of the tree. Multi-sample reference phasing analysis of the samples from A31 identified multiple MSAI events as well as parallel LOH on chromosomes 6 and 13 (Fig. 3b). MEDICC2 assigned the independent origins of these parallel events to the branch corresponding to the emergence of the dominant clone in the primary sample and to the branch corresponding to emergence of the common ancestor of all the metastatic samples (Fig. 3b). MEDICC2’s ability to correctly identify and locate these parallel evolutionary events revealed by multi-sample phasing provides additional evidence for a diverging evolutionary trajectory between primary and metastatic samples, absent from its original analysis [40].

We were further interested in whether the inferred tree topologies and SCNAs can be used to detect preferentially gained and lost regions, potential indicators of positive selection [3]. To this end, we used the oncogene (OG) and tumor suppressor gene (TSG) scores derived by Davoli et al. [41] for individual genes as well as on the level of chromosome arms. MEDICC2’s event detection algorithm (“Methods”) allows calculating the net number of gains and losses along the phylogenetic tree in regions of interest and counts events only once at the node in the tree where they occur. Across the 10 patients in this cohort, a clear correlation is visible (Pearson *r*=0.59, *p*<.0001) between the MEDICC2 event score and the OG-TSG score on the level of chromosome arms reported by Davoli et al. [41] (Fig. 4b). Additionally, on the level of all 1729 individual genes from Davoli et al., we observed a global correlation of *r*=0.09 (*p*<.0001) (Fig. 4c) which rises to *r*=0.25 (*p*=0.01) when considering only the top 100 genes. Despite the low sample size of 10 patients and the fact that the OG-TSG score was calculated on a pan-cancer dataset, the results show the ability of MEDICC2 to infer regions of interest by detecting distinct gain and loss events in the individual copy-number trees.

### MEDICC2 infers SCNA phylogenies from single-cell data

Recent advances in single-cell technology have enabled the collection of copy-number profiles of thousands of cells. While large single-cell experiments constitute a major opportunity to study tumor evolution with higher precision and on a larger scale, they also bring unique challenges. The lower coverage of single-cell studies lead to a lower signal-to-noise ratio than conventional methods and therefore to less reliable and more noisy copy-number profiles. The large number of copy-number profiles representing cells increases the computational burden, in particular for pairwise distance calculations and ancestral reconstruction. Due to the new fast composition algorithm (Fig. 1e) and an efficient parallelization strategy (“Methods” and [39]), MEDICC2 processes thousands of cells efficiently, running on 32 cores for less than 1 h (Additional file 1: Table S3).

Here, we apply MEDICC2 to a previously published single-cell study of triple-negative breast cancer by Minussi et al. [46] looking at the two patients highlighted in the paper, TN1 and TN2, with 1100 and 1023 cells, respectively. In the original study, the authors defined “superclones” and “subclones” by two separate clustering methods in the two-dimensional UMAP space created from pairwise Manhattan distances. Consensus copy-number profiles were created from these clusters and a minimum evolution tree was created from the Manhattan distances between these consensus profiles. This indirect way of determining the phylogeny of these cells involved a number of data abstractions that involved manual selection of hyperparameters (e.g., for the clustering algorithms).

**Fig. 5:**
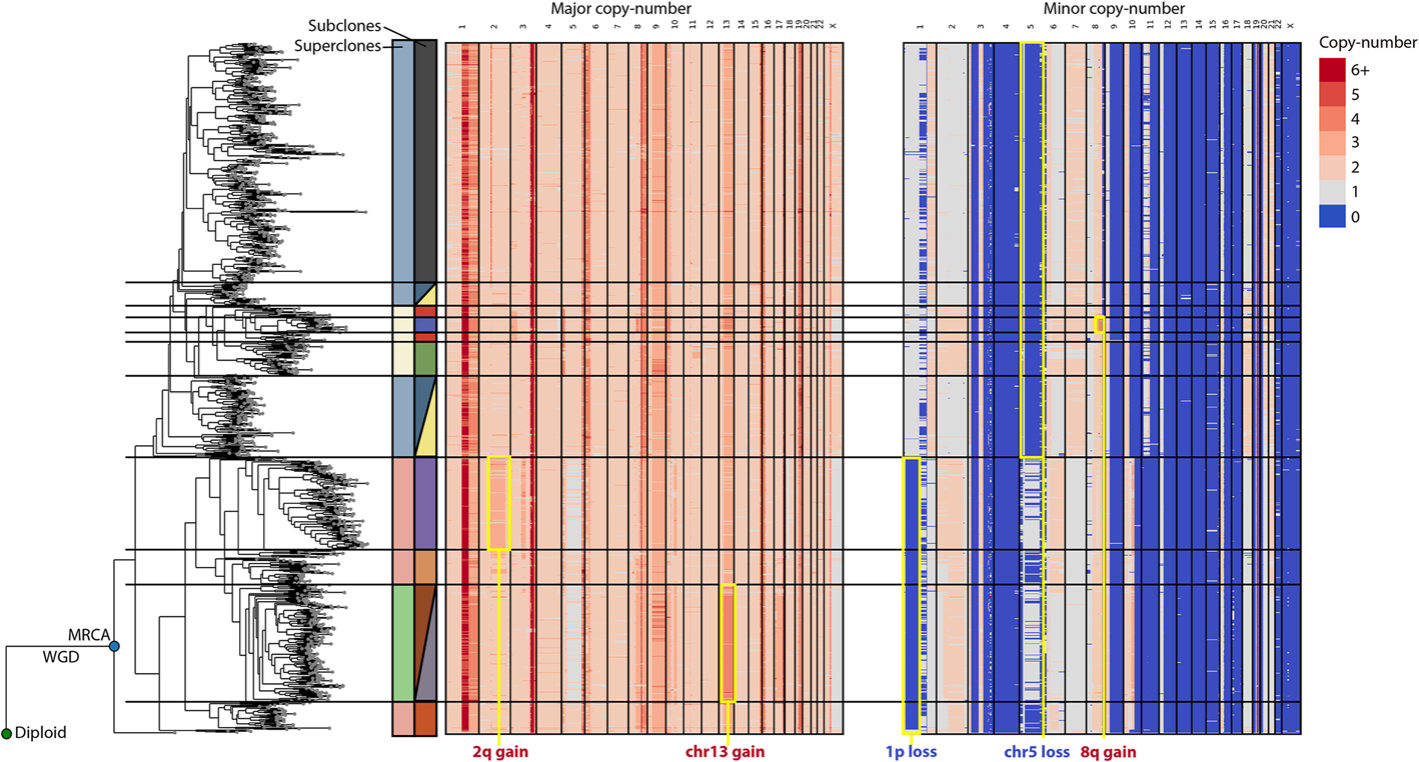
Inferred phylogeny for single-cell data with 1023 cells. Inferred phylogeny and allele-specific copy-number profiles for patient TN2 from Minussi et al. [46]. The diploid and most recent common ancestor to all cells are marked with green and blue circles, respectively. We manually selected clades from the phylogeny to match the superclones and subclones of the original publication. These are marked next to the tree in the colors of the original publication and with horizontal lines. The structure of the tree corresponds very clearly with distinct features of the copy-number profiles and matches the clonal structure derived in the original publication. Selected synapomorphies of the clone structure are highlighted with a yellow border and annotated on the figure

We instead derived allele-specific copy numbers from the original raw data (“Methods”) and ran MEDICC2 directly on the allele-specific copy-number profiles to reconstruct phylogenies for all cells without intermediate clustering steps or consensus profiles (Fig. 5 and Additional file 1: Fig. S23a). We then mapped superclones and subclones from the original publication to the MEDICC2 tree and found a high degree of concordance between the clonal architecture revealed by MEDICC2 and the original results [46], in contrast to a simple tree based on Manhattan distance between all cells (Additional file 1: Fig. S23b-c). For TN2, MEDICC2 recreates all superclones and most subclones from the original publication, while for TN1 it consolidates two superclones into one, but otherwise detects them as in the original publication. In addition, MEDICC2 correctly detected truncal WGDs from the single cells in both patients as described [46], without the need for additional whole-exome sequencing. While the original study reports truncal branch lengths similar to the maximal MRCA to leaf distance, suggesting that roughly half of the SCNA events happened before emergence of the MRCA, we find truncal branch lengths substantially shorter in the MEDICC2 phylogenies (42/164 for patient TN1 and 71/238 for patient TN2). These findings are in concordance with our results for the metastatic prostate cancer patients described above and provide further evidence for substantial clonal diversification after emergence of the MRCA.

Our analyses demonstrate that MEDICC2 infers tree topologies that provide substantial biological insight, while previous approaches using general measures such as the Manhattan distance were not able to recover the clonal architecture of the tumor (Additional file 1: Fig. S23). In contrast to clustering of consensus profiles, MEDICC2 retains single-cell information when inferring tree topologies and ancestral genomes. To the best of our knowledge, MEDICC2 is the only available algorithm that can reliably create accurate copy-number phylogenies from thousands of single cells.

## Discussion

Methods for the computationally challenging task of phylogenetic tree inference have proliferated in studies of cancer genomics. In order to make such inference tractable using SNVs, SCNAs, or combinations thereof, a number of simplifications are often employed. The infinite site assumption, in which every genomic position can mutate at most once, is almost universally applied despite its frequent violation by both SNVs and SCNAs in cancer [3, 47, 48]. Another common simplification that is violated by SCNAs is to assume independence of adjacent genomic loci. Existing methods variously attempt to overcome this issue by utilizing pseudo single-site mutations [49] or by considering breakpoints only [38]. Finally, many methods do not solve the NP-complete Steiner tree problem of phylogenetic inference [50], but instead solve the much simpler minimum spanning tree problem, identifying a tree that optimally connects only the sampled clones, ignoring ancestral or potentially unsampled populations, allowing inference in polynomial time [28].

MEDICC2 reconstructs the evolutionary history of cancer from haplotype-specific SCNA profiles without these assumptions. It employs a new explicit evolutionary model of copy-number change which includes WGD events and computes the MED [6] in time linear in the number of genomic segments and at a fraction of its original runtime. Unlike MEDALT [28], MEDICC2 addresses the Steiner tree problem [50], finding not only an approximately optimal tree topology but, importantly, the ancestral states connecting the taxa under an evolutionarily consistent model. As an additional difference, MEDICC2 computes distances between any pair of haplotype-specific copy-number profiles via a common ancestor, incorporating irreversible changes such as LOH affecting either haplotype or homozygous deletions, states that are frequently observed in tumor evolution [25, 51]. While the algorithm is overall quadratic in the number of samples, our efficient parallelization allows its application to single-cell datasets consisting of thousands of cells. MEDICC2 additionally extracts individual SCNA events, including clonal and subclonal WGD and provides statistical robustness assessment of the inferred trees.

One limitation of our work is that we only consider genomic alterations that change copy number and assume that all alterations are contiguous with the reference genome. Additionally, the MEDICC2 model does not explicitly represent complex genomic rearrangements, such as breakage-fusion-bridge cycles or chromothripsis, but instead relies on basic elementary operations to recreate such complex events. Despite this, our simulations have shown that MEDICC2 accurately infers phylogenies in the presence of complex events, outcompeting alternative methods, and that it is substantially robust to violations of the contiguity assumption by for instance translocations and inversions. By comparing the MEDICC2 events with SV data, we furthermore confirmed that MEDICC2 events are rooted in actual biological processes and more accurately reflect genome evolution than measures based on copy-number segments alone. In the future, complex events might be integrated explicitly into the MEDICC2 model, depending on the additional computational complexity required, to further improve reconstruction accuracy.

Similarly, WGD in cancer has been proposed to result from endoreduplication [52], mitotic dysfunction [53], or cytokinesis failure [54]. Nearly all of these mechanisms suggest a diploid to tetraploid transition (tetraploidization) [55], frequently followed by return to a near-triploid state through subsequent chromosome losses [20], possibly with preceding LOH events [56]. MEDICC2 can replicate this behavior naturally through the combination of LOH events, a WGD event and multiple independent chromosome-wide losses. As every chromosome loss is counted as a separate event, the MED might overestimate the true number of events. However, our model seems to fit real-world copy-number profiles extremely well as verified on 2778 WGS tumors from the PCAWG cohort.

Our recent work [3, 4] and that of others [44] has highlighted the importance of using multi-sample phasing to reveal additional SCNA heterogeneity taking the form of MSAI or parallel evolution of similar SCNAs from distinct haplotypes [3, 4]. Since MEDICC2 does not employ the infinite site assumption, it can reveal homoplasy on alternating haplotypes (MSAI) as well as on the same haplotype where an independent origin of two events leads to a more parsimonious phylogeny than a shared ancestry. In the Gundem et al. cohort [40], MEDICC2 provided additional support for the divergence of the primary and metastatic samples through the detection of a subclonal WGD event and parallel evolution in A31. Analysis of the net gains and losses of chromosome arms and individual genes along the inferred trees for all patients showed a clear correlation with the OG-TSG score [41]. This demonstrates the ability of MEDICC2 to find genomic events potentially under positive selection for clinical interpretation of tumor evolution. In the future, analysis of a larger cohort could yield further insights into preferentially gained and lost regions of the genome in a cancer-type-specific way.

Since MEDICC2 does not itself infer SCNAs from either bulk or single-cell raw data and is agnostic of the sequencing modality used to generate its input, its results are dependent upon accurate cell, bulk sample, or subclone-level copy-number profiles, and the resolution of the original data. Future advances, for example in single-cell sequencing [57] for total [58] and allele-specific copy-number calling [59], or through co-inference of copy-number and tree topology [60], that increase resolution and decrease noise levels will be usable by MEDICC2 without modification. As we have shown, MEDICC2 can process thousands of single cells and thereby infer inter- and intra-region evolution. It outperforms pairwise Manhattan distances from the original study, creating a tree topology that matches the previously identified super- and subclones with high accuracy directly from single-cell data without additional parameter fitting or the creation of consensus profiles.

## Conclusions

In summary, systematically determining the number and order of WGD, arm-level SCNAs, and focal events that have occurred in the evolutionary history of a tumor has not yet been performed on a large scale and has previously been the preserve of theoretical mathematical modelling [61, 62]. MEDICC2 enables the reconstruction and timing of the individual SCNA events present in the evolutionary history of a tumor that may overlap and build upon one another. This will allow much more detailed dissection of WGD, aneuploidy, and CIN across the genome utilizing single-sample, multi-sample, and single-cell approaches, than the measures of the proportion of the genome affected by SCNA that much of the field has previously relied on.

## Methods

### The MEDICC2 model

To solve the MED problem, we employ a finite-state transducer (FST) framework as previously described [6], following the notation of Mohri [63] (Additional file 1: Fig. S1a). Copy-number profiles are represented as vectors of positive integer copy numbers (*k*)_1. . *n*_ , with 0 ≤ *k* ≤ 8 , where each integer copy number represents a genomic segment *i*. Chromosome boundaries are marked by a chromosome separator character “X” and both haplotypes are concatenated and separated by “X.” We represent these allele-specific copy-number profiles as unweighted finite-state acceptors (FSA) *A* = (*Σ*, *Q*, *E*, *i*, *F*) (Additional file 1: Fig. S1c) and evolutionary events as weighted FSTs *T* = (*Σ*, *Q*, *E*, *i*, *F*, *λ*, *ρ*) with (input and output) copy-number alphabet *Σ* = {0, .., 8, *X*} (per allele), a finite set of states *Q*, a finite set of transitions *E*, an initial state *i* ∈ *Q*, a set of final states *F* ⊆ *Q*, an initial weight *λ*, and a final weight *ρ* (Additional file 1: Fig. S1d-f). Transitions between states are equipped with an input symbol *l*_*i*_ ∈ *Σ* (input copy number) and an output symbol *l*_*o*_ ∈ *Σ* (output copy number) and a weight *w*. All weights *λ*, *ρ*, *w* are taken from the positive integers including zero and calculations are carried out over the tropical semiring, i.e., weights are summed along the path of a FST and the final weight between a pair of sequences is the minimum over all possible paths

1 $$T\left[x,y\right]=\underset{\pi \in P}{\min}\ {\sum}_i \quad w{\left[\pi \right]}_i$$

(see [35]), where *P* is the set of all possible paths transforming *x* to *y*.

FSTs and FSAs can be subjected to a variety of operations, of which *composition* (“∘”) is of particular importance. During composition, a new FST is constructed in which the set of states is the cartesian product of the set of states of the two input FSTs. The composition *S* of two FSTs *T*_1_ and *T*_2_ then assigns a weight to any pair of input and output sequences by chaining their transduction

2 $$S\left[x,y\right]=\left({T}_1\circ {T}_2\right)\left[x,y\right]=\underset{z}{\mathit{\min}}\ \left({T}_1\left[x,z\right]+{T}_2\left[z,y\right]\right)$$

via intermediary sequence *z* [63]. Composition is also used to effectively compute the score or total weight *T*[*x*, *y*] that a FST *T* assigns to a pair of sequences *x* and *y* (Eq. 1) by representing *x* and *y* as two unweighted acceptors and running a single-source shortest distance algorithm (SD) over the composition *x* ∘ *T* ∘ *y* [63].

3 $$T\left[x,y\right]=\underset{\pi }{\min}\ {\sum}_i\quad w{\left[\pi \right]}_i= SD\left(x\circ T\circ y\right).$$

Composition enables us to combine multiple evolutionary event FSTs into a final FST in which the individual events are carried out successively in order of composition, and to transform the asymmetric MED into a symmetric MED for calculation of the pairwise distance matrix [6] (Additional file 1: Fig. S1g).

### Calculating the minimum-event distance

It has been shown previously that the standard MED can be solved by considering losses separately before any gains [33]. Indeed, only loss-of-heterozygosity (LOH) events, i.e., losses which reduce haplotype-specific copy numbers to zero, must be considered first, as subsequent gain and loss events must ignore the positions with copy-number zero. The MED however is oblivious to the ordering of any subsequent gains and losses. When including WGD events (MED-WGD), LOH events must again be dealt with before any other event. In addition, WGD events must come before any segmental losses and gains, for example to allow for the deletion of segments previously gained during a WGD event (Fig. 1b, Additional file 1: Fig. S1b,g). The inclusion of WGD events further introduces non-determinism into the problem as locally WGD events cannot be distinguished from segmental gains before taking the full sequence into consideration (Additional file 1: Fig. S1b,h).

We thus define four one-step FSTs which model one of four different evolutionary events considered: (i) LOH events (*T*^1^_*LOH*_), (ii) segmental (+1) gains (*T*^1^_*G*_), (iii) segmental (-1) losses (*T*^1^_*L*_) without LOH, and (iv) WGD (+1 for all non-zero segments) events (*T*^1^_*WGD*_). LOH events, gains, and losses must terminate when they reach separator character “X” (Additional file 1: Fig. S1d,f). WGD events do not terminate at “X” and leave it unchanged (Additional file 1: Fig. S1e). In the one-step FSTs, each sequence position can only be affected by a single event. For example, the one-step FST for segmental gains *T*^1^_*G*_ only allows copy-number changes of arbitrary length from 1 to 2, 2 to 3, and so on, but not, for example, from 1 to 3. To span the full range of possible events, the one-step FSTs are each composed *n* times with themselves and the maximum copy-number dictates the number of compositions necessary: *n* =  ∣ *Σ* ∣  − 1 for LOH events and *n* =  ∣ *Σ* ∣  − 2 for segmental losses and gains and WGD events [6]. The resulting event FSTs *T*_*LOH*_, *T*_*G*_, *T*_*L*_, and *T*_*WGD*_ are then chained (composed) into the asymmetric MED-WGD FST.

4 $$T={T}_{LOH}\circ {T}_{WGD}\circ {T}_L\circ {T}_G$$

The final MED-WGD between copy-number profiles *x* and *y* is then computed following Eq. 3 (Additional file 1: Fig. S1g):

5 $$T\left[x,y\right]= SD\left(x\circ T\circ y\right)= SD\left(x\circ {T}_{LOH}\circ {T}_{WGD}\circ {T}_L\circ {T}_G\circ y\right)$$

Analogously, the simple MED is built via composition as in

6 $$T={T}_{LOH}\circ {T}_L\circ {T}_G$$

and distance calculation is carried out as in Eq. 5.

As noted previously the MED and MED-WGD are asymmetric. To compute symmetric distances *S*[*x*, *y*] between pairs of copy-number profiles connected in a phylogenetic tree, we compute the score between *x* and *y* via its common ancestor using the kernel composition of *T* with its inverse *T*^−1^[64, 65] (Fig. 1c):

7 $$S\left[x,y\right]= SD\left(x\circ {T}^{-1}\circ T\circ y\right)$$

As the number of states in a composed FST is the product of the states of the input FSTs, explicit computation of the composition in Eq. 7 is computationally expensive. We therefore employ a new computation strategy based on lazy (on-demand) composition followed by shortest-path computation using a shortest-first queue [66]. Lazy composition prevents full expansion of the composed FST before determining the shortest path and instead expands the FST only along the path visited [66].

### MED speed and accuracy evaluation

To assess the performance of the new lazy composition strategy and the accuracy of the MED calculation, we simulated copy-number profiles following the MEDICC2 evolutionary model. A random number of evolutionary events was generated using a Poisson process with rate parameter *μ* = 10 (reconstruction accuracy test) and *μ* = 20 (speed test). In the reconstruction accuracy test, each event had a 5% probability to be a WGD event, and a 47.5% probability of being a gain or loss respectively. In the speed test, to prevent too many deletions, the gain probability was set to 80%. The start of an event was selected uniformly at random from the set of remaining available positions (positions with copy number ≠0) and event lengths were drawn from a geometric distribution with success probability parameter *p* = 0.2. Events were applied to the sequence obeying biological constraints, i.e., no gain of segments with copy-number zero and forced ending of events at chromosome boundaries, the latter with the exception of WGD. For the reconstruction accuracy test, sequences were fixed at length 50 (five chromosomes of length 10 each). For the MED speed test, sequence lengths were varied from 20 to 200 segments (Fig. 2a).

### Linear time evolutionary phasing

Traditionally, allele-specific SCNAs are reported in major and minor copy number, as the relative phasing of copy-number segments to each other is unknown. We introduced the multi-sample reference phasing implementation *Refphase (version 0.3.0)* [3, 67] to leverage relative phasing information in a multi-sample sequencing scenario and used it to identify MSAI events across human cancers [3, 4, 45]. In situations where multi-sample reference phasing is not feasible, e.g., in single-sample scenarios, we developed evolutionary phasing [6], where the assignment of major and minor copy numbers to parental haplotypes is chosen to minimize the sum of MEDs over both parental haplotypes (minimum evolution criterion). In its original form, evolutionary phasing was achieved through the use of a weighted context-free grammar in concert with our original MED [6], a computationally costly solution. To enable phasing for a large number of segments and genomes, we here provide a novel phasing strategy which solves the evolutionary phasing problem exactly, but at a fraction of the original runtime, by staying within the realm of regular grammars and FSAs.

To do so, we first encode copy-number profiles for both alleles jointly as an unweighted phasing FST *P* as follows: the FST follows a linear structure with a number of states equal to the number of segments +1. Two transitions occur between each neighboring pair of states and the two transitions have as input symbols major copy numbers and as output symbols minor copy numbers and vice versa (Fig. 1e). Due to these mirrored input and output symbols every valid path through the phasing FST *P* thus determines an assignment of copy-number alleles to haplotype 1 and haplotype 2. The set of of all 2^*n*^possible paths for a sequence of length *n* through this FST corresponds to the set of possible phasing choices. To choose the most parsimonious haplotype assignment, this phasing FST is then composed from the left and from the right side with a composed FSA *u* = (*d* ∘ *T*)↓of the diploid FSA *d* (encoding all-1s) with the MED-WGD FST *T*, projected to its output (↓). Shortest-path (SP) computation over this composite yields the optimal phase with a total score equal to the sum of MEDs over both parental haplotypes. Separate haplotypes *h*_*a*_ and *h*_*b*_ can be extracted by projection to input and output followed by weight removal:

8 $$\begin{array}{c}h_a=\uparrow SP\;\left(u\circ P\circ u\right)\\h_b=SP\;\left(u\circ P\circ u\right)\downarrow\end{array}$$

### Simulating genome evolution

To evaluate the performance of MEDICC2’s tree reconstruction algorithm, first a tree topology for a given number of leaves was created by randomly joining sample labels and rooting the tree at the diploid. The branch lengths and therefore the number of events per branch were determined using a Poisson distribution with *λ* = *Δt* · *S* · *μ*, where *Δt* was set to 1, *S* represents the length of the genome (440 segments, see below), and *μ* represents a variable rate parameter. To avoid biases, somatic evolution was modelled on the level of the genome, not the copy-number level, along the tree starting at the diploid. We chose 2 × 22 chromosomes (two sets of haplotypes) with 10 segments of uniform size each which resemble the makeup of many actual bulk copy-number profiles. At every branch, the genome was mutated with a number of genomic events based on the corresponding branch length. These events encompass gains and losses of whole chromosomes, focal losses, insertions, breakage-fusion-bridges (BFB), whole-genome doublings (WGD), and copy-number neutral events such as balanced and unbalanced translocations and inversions. For example, if a segment from chromosome 1 is moved to chromosome 2 through an unbalanced translocation and chromosome 2 is subsequently gained, the segment of chromosome 1 is also gained. In order to prevent the occurrence of homozygous deletions, we prevented deletions of haplotype 2 which in turn also lowered the effective loss to gain ratio. By choosing this approach, we ensure that the simulation is not biased towards the approach of MEDICC2 (which rather models the evolution of copy-number profiles and not individual segments) and mirrors actual tumor evolution. In the absence of actual event probabilities, we kept all events to be equally likely with the exception of BFBs and WGDs. The probability of BFBs was set to 10% of the other events and for the WGD we chose four different probabilities: 0.000125 for the simulation of large trees reminiscent of single-cell experiments (Fig. 2b), and three levels for the simulation of medium-sized trees (0 for “No WGD”, 0.0125 for “Low WGD” and 0.065 for “High WGD” (Additional file 1: Fig. S3a).

For the large tree scenario, we simulated 25 trees each for all combinations of the mutation rate *μ* ∈ [0.01, 0.025, 0.05] and the number of leaves *N* ∈ [5, 10, 15, 20, 50, 100, 250, 500]. For the medium tree scenario, we simulated 25 trees each for all combinations of the mutation rate *μ* ∈ [0.01, 0.025, 0.05] and the number of leaves *N* ∈ [5,10,15,20] and the three levels of WGD as described above.

In order to check the effect of the assumption that segments are contiguous with respect to the reference genome, we simulated trees with rate *μ* = 0.05 for *N* = 20 leaves. Here we restrict the simulation to only create gains/losses (both focal as well as chromosomal) as well as translocations and inversions. In the absence of translocations and inversions, we expect a total number of 400 gain and loss events. We now increase the number of translocations and inversions up to a ratio of 25 (ratio between number of translocations/inversions and gains/losses) (Additional file 1: Fig. S4).

Reconstructed trees were evaluated using the generalized Robinson-Foulds (GRF) distance as implemented in the R package *TreeDist* [68]. The GRF is based on the widely used Robinson-Foulds distance which measures the number of splits that occur in both trees. The GRF improves this metric by taking the similarity of splits that are not perfect matches into account. We furthermore used the regular Robinson-Foulds distance (as implemented in the R package *ape* [69]) and the Quartet distance (as implemented in the R package *Quartet*) to prevent any potential biases from the tree metric used (Additional file 1: Fig. S3c).

### Comparison to other methods

We compared MEDICC2 to a range of widely used methods which encompassed Euclidean- and Hamming-distance-based trees created both through neighbor joining and minimum evolution. For neighbor joining, we used the implementation of MEDICC2 and for the minimum evolution tree we used the function *fastme.bal* from the R package *ape* [69].

As a representative of algorithms that create minimum spanning trees (MST), we compared against MEDALT[28] and as a representative of methods based on changepoints we compared against Sitka [38] (Additional file 1: Fig. S3b).

MEDALT was run with default parameters. After running MEDALT, we transformed the minimum spanning trees into phylogenies. To this end, we replaced all cells that are positioned on internal nodes of the tree with dummy nodes and added the samples back in with branch length zero as children leaves of the respective dummy nodes. Note, that the minimum spanning trees can create multifurcations which we cannot resolve and are left as multifurcating trees in the phylogeny.

Sitka was run as instructed by its GitHub Readme page (as of 01.06.2022) using the parameters that were used in the original publication for real datasets (taken from Supplemental Table 2) [38]. We removed internal nodes that only had a single child node.

As Sitka is based on a perfect phylogeny assumption, it places breakpoints as the internal nodes of the phylogenetic tree. Some resulting trees wrongfully placed these breakpoints as leaf nodes instead of internal nodes. We removed these leaf nodes in order to make the tree comparable to the simulations.

### Validation dataset

In order to validate MEDICC2’s event detection algorithm, we applied MEDICC2 to 2778 single-region tumor samples from the Pan-cancer Analysis of Whole Genomes (PCAWG) [34]. The copy-number profiles, SVs, and WGD status of the individual samples were downloaded from the ICGC Data Portal (https://dcc.icgc.org/releases/PCAWG/). All samples were phased using MEDICC2’s evolutionary phasing algorithm.

### Overlap of MEDICC2 events with structural variants

To assess the accuracy of the reconstructed MEDICC2 events, we selected all MEDICC2 events which overlap on at least one boundary with the boundary of a SV (100kbp proximity). Next we checked whether the other boundary of the MEDICC2 event also coincided with the other boundary of the same SV (“supported events”). The number of supported events was compared to two null models: firstly, using the next segment boundary and, secondly, using a random segment boundary on the same chromosome. The “next segment boundary” was defined as the closest segment boundary to the initial overlapping breakpoint in the direction of the other SV boundary. That means if the initial overlap matches the start of a SV, the next boundary will be chosen downstream. Similarly, the random segment boundary was chosen from all possible segment boundaries between the initial overlap and the end of the chromosome in the direction of the second SV boundary.

As the dataset contains many subclonal SVs that are not present in the copy-number profiles of the samples, we removed all SVs that did not overlap with a copy-number change on both breakpoints. Events and SVs were filtered at different minimum sizes (100kbp, 1Mbp, and 10Mbp) and also filtered based on the type of SV (either all types or solely deletions and duplications, Additional file 1: Fig. S7). Only samples that had at least 10 MEDICC2 events and 10 SVs were selected for the analysis (1476 samples for 100kbp, 1221 for 1Mbp, and 850 for 10Mbp).

### WGD detection

To facilitate WGD detection, we calculate the MED with (MED_WGD_) and without (MED_noWGD_) the possibility of WGD between each PCAWG tumor and a diploid normal sample and computed the WGD evidence score *s*_*i*_ as

$${s}_i=\textrm{ME}{\textrm{D}}_{\textrm{noWGD}}\left(d,{t}_i\right)-\textrm{ME}{\textrm{D}}_{\textrm{WGD}}\left(d,{t}_i\right),$$

where *t*_*i*_ represents a PCAWG tumor profile and *d* represents a standard diploid normal sample. Because MED_noWGD_(*x*, *y*) >= MED_WGD_(*x*, *y*) for any valid set of copy-number profiles *x* and *y*, the score *s*_*i*_ is always non-negative (*s*_*i*_ ≥ 0) and a score of *s*_*i*_ ≥ 1 indicates a preference for a WGD event to have occurred.

By replacing the multi-step WGD transducer *T*_WGD_ in (Eq. 4) with *n*-step WGD transducers for variable *n*, we can test for multiple WGD events. For example, the scores MED_1 WGD_(*x*, *y*) > MED_2 WGDs_(*x*, *y*) = MED_3 WGDs_(*x*, *y*) indicate two WGDs to have taken place.

In order to increase the robustness of our predictions, we repeated the analysis with 100 bootstrap runs (see below). Samples that exhibited WGDs (or multiple WGDs) in at least 5% of the bootstrap runs were classified as WGDs (or multiple WGDs, respectively).

The effect of the bootstrap percentage threshold on the final outcome is explored in Additional file 1: Fig. S11.

### Event detection and correlation with OG-TSG score

For comparisons between events detected in MEDICC2 and the OG-TSG score, we downloaded 1729 gene annotations from Davoli et al. [41] and the aggregated chromosome-arm-wise OG-TSG scores that measure the occurrences of OGs and TSGs on a given arm. To extract events, we leverage the ancestral reconstruction routine in MEDICC2. Trees are then traversed in postorder. Relative copy-number changes are determined for all segments and events are counted in the branch where the change occurs, thereby taking parallel evolution into account while preventing counting the same event multiple times in multiple samples from the same patient. Change events were then overlapped with regions of interest, i.e., the positions of OGs and TSGs as well as the chromosome arms. An event is detected if there is at least 90% overlap between the event and the region of interest. Gains and losses are summed across all branches and patients to arrive at the final “#gains - #losses” score for each gene / chromosome arm. The event detection routine is available to MEDICC2 users by providing BED files with regions of interest and MEDICC2 can calculate the number and exact location of gains/losses of these regions along the evolutionary trajectory.

### Resampling for robustness estimation

The bootstrap [70, 71] is a classical approach in phylogenetics to assess the robustness of an inferred tree to perturbations of the data. During bootstrapping of a multiple sequence alignment, columns are drawn from the original data with replacement and a large number of resampled datasets (typically 100–1000) are created. The tree reconstruction method of choice is then employed on all bootstrap datasets and the relative frequency with which a branch (or taxon split) of the original tree appears in the set of bootstrapped trees forms a support value for this branch. A necessary requirement for this approach is the independence of sites in the alignment. Since this assumption does not hold for copy-number profiles, we use the following alternative resampling strategies for copy-number profiles in MEDICC2:

1. Chromosome-wise bootstrap: Here, whole chromosomes are drawn with replacement from the original chromosomes to create a bootstrap sample. As losses and gains end at chromosome boundaries and as WGD events are ignorant to the order and number of chromosomes, this approach does not introduce false events while still providing a sufficiently large sample space, albeit at the cost of a coarse-grained resolution. Therefore, not all bootstrap samples will be equally representative of the underlying data.
2. Segment-wise natural jackknife: Here, N segments are drawn with replacement from the original N segments, discarding all duplicates. On average, this is equivalent to discarding $\frac{1}{e}$ randomly selected segments [72]. The jackknife approaches the bootstrap distribution and due to the lower number of resulting segments has a speed advantage over the chromosome-wise bootstrap. However, the jackknife generally generates less accurate representations of the original data than the bootstrap. Branch support values are indicated by their percentage value on the respective branches (see Fig. 3b).

### Parallelization strategy

Single-cell experiments with thousands of cells demand high-performing methods as the pairwise distance calculations scale with *O*(*N*^2^) and are therefore exceptionally computationally expensive. In addition to the performance improvements when calculating the MED, we implemented a parallelization routine to make MEDICC2 applicable to hundreds to thousands of cells. To this end, we utilized a recently proposed parallelization strategy [39] to split the *N* × *N* pairwise distance calculations into smaller chunks that can be run in parallel. In the method used, the N samples are split into *p*^2^ + *p* groups of size *p* (where *p* is the smallest prime such that *p*^2^ ≥ *N*) and the pairwise distances within the individual groups are calculated such that a given pair is never calculated twice. This allows for a theoretical speed-up by the factor *p*^2^ + *p* which for all practical concerns is only limited by the number of available cores [39].

### Bulk data processing and analysis

The bulk SCNA analysis was performed in two stages. First, as part of the PCAWG cohort [25], each tumor sample was analyzed individually with the Battenberg algorithm [73] to produce a sample-level inferred purity, ploidy, and copy-number segmentation with associated allele-specific copy-number states. Only copy-number segmentation from autosomes was included in the study. The Battenberg algorithm is able to detect subclonal SCNAs. However, in our analyses, we used only a single full genome-wide copy-number profile representing the dominant subclone per tumor sample comprising both clonal SCNAs and those subclonal SCNAs present in ≥50% of tumor cells within that sample.

Next, these Battenberg outputs, as well as the input data used for Battenberg including heterozygous SNP B-allele frequencies, for all samples from a tumor, were jointly analyzed to produce haplotype-specific SCNAs through the application of Refphase, a multi-sample reference phasing algorithm [3]. We then defined a tumor consensus segmentation profile by combining breakpoints from each SCNA segmentation profile from each individual tumor sample. This tumor-level analysis of haplotype-specific SCNAs may reveal instances of mirrored subclonal allelic imbalance (MSAI) [3, 4] in which SCNAs that affect the opposite haplotypes in different samples from the same tumor result in different haplotypes having a higher copy number in different samples. This causes the identity of the heterozygous SNPs belonging to the most prevalent haplotypes to differ between tumor samples.

A subset of these MSAI events may be considered parallel events. These parallel events involve the same class of SCNA, for example a gain on the “A” haplotype in one sample (e.g., from “AB” to “AAB”) and an independent gain on the “B” haplotype in another sample (e.g., from “AB” to “ABB”) may constitute evidence for convergent evolution and positive selection. In contrast, independent evolution involving differing classes of SCNA in different samples from the same tumor may result in MSAI but not constitute parallel evolution of the same class of SCNA. An example of this could include a gain of “A” in one sample resulting in an “AAB” copy-number state and a loss event in another sample resulting in a lone copy of the “B” allele.

Reference phasing, as described above, considers all genomic segments independently of each other, and while often segments span entire chromosomes, sometimes different reference samples may be chosen for different segments on the same chromosome. Since phasing is restricted to continuous regions of allelic imbalance, we estimate phasing along the genome across multiple segments within chromosomes (“horizontal phasing”) by using an evolutionary criterion. Briefly, the assignment of heterozygous SNPs to “A” and “B” haplotypes for all bins within a single chromosome is chosen to minimize the number of copy-number events between a diploid normal sample and the tumor.

### Bulk phylogeny comparison

The SNV-based phylogenetic reconstructions were reproduced from Gundem et al. “Figure 2: Subclonal structure within 10 metastatic lethal prostate cancers.” with additional information from “*Supplementary Table - Subclones*” (also from Gundem et al.) using the following columns: “*cluster*” that details the clusters used to generate the original phylogenies, “*cluster.colour*” that is the cluster’s color in the original publication, “# subs from WGS data” that shows the number of SNVs present within each cluster, “*samples containing*” that shows which samples contain each cluster, and “*CCF values*” that show the cancer cell fraction values for each cluster in each sample that it is present in. The branch lengths of these phylogenies are determined by the number of SNVs present in each cluster that contributes to the branch.

We compared unnormalized root-to-leaf lengths between the SNV-based clone phylogenies and the MEDICC2 phylogenies at the cohort level, and when discussing individual tumors, we normalized each root-to-leaf length by the maximum observed root-to-leaf length of the corresponding phylogeny for that tumor. To compare topologies, we used the regular Robinson-Foulds distance (as implemented in the R package *ape 5.6.1*) to evaluate similarity between the SNV-based clone phylogenies and the MEDICC2 trees. We took the leaves as annotated in “Fig. 2: Subclonal structure within 10 metastatic lethal prostate cancers.” from Gundem et al. When multiple samples were listed as a single leaf, we introduced a bifurcation at this position, e.g., tumor A12 with leaf AC. In addition, when multiple samples were listed in multiple leaves, we chose the leaf with each sample’s largest CCF contribution to represent that sample. Finally, the Gundem et al. trees contain multifurcations, which support a variety of possible bifurcating trees. For comparison with a binary hypothesis tree as inferred by MEDICC2, we considered all possible bifurcations obtainable from any given multifurcation and used the minimum Robinson-Foulds distance across this set of bifurcations to determine a possible match.

### Single-cell data processing and analysis

Segmented log ratios of read counts within genomic bins and total copy-number profiles of single-cell triple-negative breast cancer data were obtained from ref [46]. Allele counts at 1000G SNP positions were obtained for each single cell using alleleCounter (v.4.0.0) as described in ref [46].

#### Fitting to integers

The log ratios were centered to zero by subtracting the mean to obtain the logR. logR values were fitted to integers by identifying the offset *ψ* that minimizes the sum of distances across segments of the *n*_tot_=logR-*ψ* to their values rounded to the closest integers round (*n*_tot_), weighted by the lengths of the segments *w*: *argmin*_*ψ*_∑_*i* ∈ *segments*_*w*_*i*_ × (*n*_*tot*, *i*_ − *round*(*n*_*tot*, *i*_))^2^ ∣ *n*_*tot*, *i*_ =  *log* (*R*_*i*_) − *ψ*. In the original publication, the log ratios were fitted to integers by using the same fluorescence-activated cell sorting (FACS) ploidy value as the offset for all cells. Since individual cells can harbor private SCNAs, their ploidy can indeed vary around their average FACS ploidy. Therefore, we derived the average number of copies along the genome calculated from the published total copy-number profiles (~ initial value of *ψ* according to FACS ploidy) and performed a search within {0.85*ψ*, 1.15*ψ*} by steps of 0.01 to further optimize the offset to minimize the distance to integers within each individual cell.

#### Getting haplotype-specific copy-number profiles and identifying heterozygous SNPs

Across all cells from the same patient, allele counts were summed to get a pseudo bulk profile. FACS sorting based on ploidy enriches for tumor cells, but still 10–15% of cells were normal contaminants [46]. Thus even in LOH regions, heterozygous SNPs can be identified. As described in ref [46], heterozygous SNPs with allele counts for genotype A and B, c_A_ and c_B_, were defined as those with P(Bin(c_A_ + c_B_, 0.99) ≤ c_A_) < 0.01 and P(Bin(c_A_ + c_B_, 0.99) ≤ c_B_) < 0.01. At each heterozygous SNP position, the genotype with the highest read count in the pseudo bulk was assigned to the major allele.

#### Fitting within cells

After phasing all heterozygous SNPs, for each segment, the maximum likelihood estimate of the BAF *b*_mle_ is derived as follows: from each *b* belong to the possible values between 0 and 1 by steps of 0.001, *b*_mle_ is the value of the BAF *b* that maximizes the likelihood of a Binomial distribution with probability *b*, number of successes is the total number of reads bearing the genotypes assigned to the major allele, and the number trials is the total number of reads.

#### Fitting across cells

To account for the noise in *n*_tot_ and BAF, copy-number states of each segment are assigned by fitting these data to integers across cells. Each cell’s segment is assigned to allele-specific copy-number states as follows: first, it is assigned to its closest integer allele-specific copy-number state, i.e., {round(*n*_tot_*BAF), round(*n*_tot_)-round(*n*_tot_*BAF)}; second, at each populated allele-specific copy-number state across cells, the noise parameter for a Gaussian distribution is estimated from the non-rounded integers, with the mean being the total integer corresponding to the integer state, and the parameters for a Beta distribution are estimated from the segments’ BAF values, keeping the mean of the Beta as the BAF of the corresponding integer state; then, each cell’s segment is re-assigned to the allele-specific copy-number states that minimize the sum of its LogR and BAF likelihoods normalized across states; the weight given to the likelihood from the LogR can be modulated to best assign states from diploid cells ((1.9<ploidy<2.1) to {1,1} across segments (here, 50% more weight was given to the likelihood from the LogR); and the second and third steps are repeated a hundred times or until convergence.

Using the major minor configuration of the data as described above, MEDICC2 was run with standard settings on 32 cores for patient TN1 and TN2 of the cohort. By looking at the final tree and the corresponding copy-number profiles, clades in the tree were manually assigned to the corresponding super- and subclone of the original publication. In order to recreate the minimum evolution trees from the original publication [46], we created phylogenies using the function *fastme.bal* from the R package *ape* [69] based on the pairwise Manhattan distance.

## Supplementary Information

Additional file 1: Supplementary Figures S1-S23 and Supplementary Tables S1-S3.

Additional file 2. Peer review history.

## Data Availability

MEDICC2 is implemented in Python 3 and freely available under GPLv3 on Bitbucket (https://bitbucket.org/schwarzlab/medicc2) [74] and in the Bioconda repository. MEDICC2 uses OpenFST 1.8.1 and its Python wrapper pywrapfst for manipulation of finite-state machines. Core algorithms are implemented in C++ as a Cython extension by linking to the OpenFST library. All data and code to reproduce the figures of this publication are present in the repository. All code relating to this publication is also available at Zenodo [75].
