# Supplementary material for: MEDICC2: whole-genome doubling aware copy-number phylogenies for cancer evolution
Source: Genome Biol. 2022 Nov 14;23:241. doi: 10.1186/s13059-022-02794-9 (PMC9661799; doi:10.1186/s13059-022-02794-9)
Supplement: Supplementary file 1 — Additional file 1: Supplementary Figures S1-S23 and Supplementary Tables S1-S3. [file 13059_2022_2794_MOESM1_ESM.pdf]

Additional file 1

for MEDICC2: whole-genome doubling aware copy-number phylogenies for cancer evolution

Tom L Kaufmann[*,#], Marina Petkovic[*], Thomas BK Watkins[*], Emma C Colliver, Sofya Laskina, Nisha Thapa, Darlan C Minussi, Nicholas Navin, Charles Swanton, Peter Van Loo, Kerstin Haase, Maxime Tarabichi, Roland F Schwarz[#]

* these authors contributed equally

# corresponding authors: roland.schwarz@uni-koeln.de; tom.kaufmann@mdc-berlin.de

Supplementary Figures 2

Supplementary Tables 25

Supplementary Figures

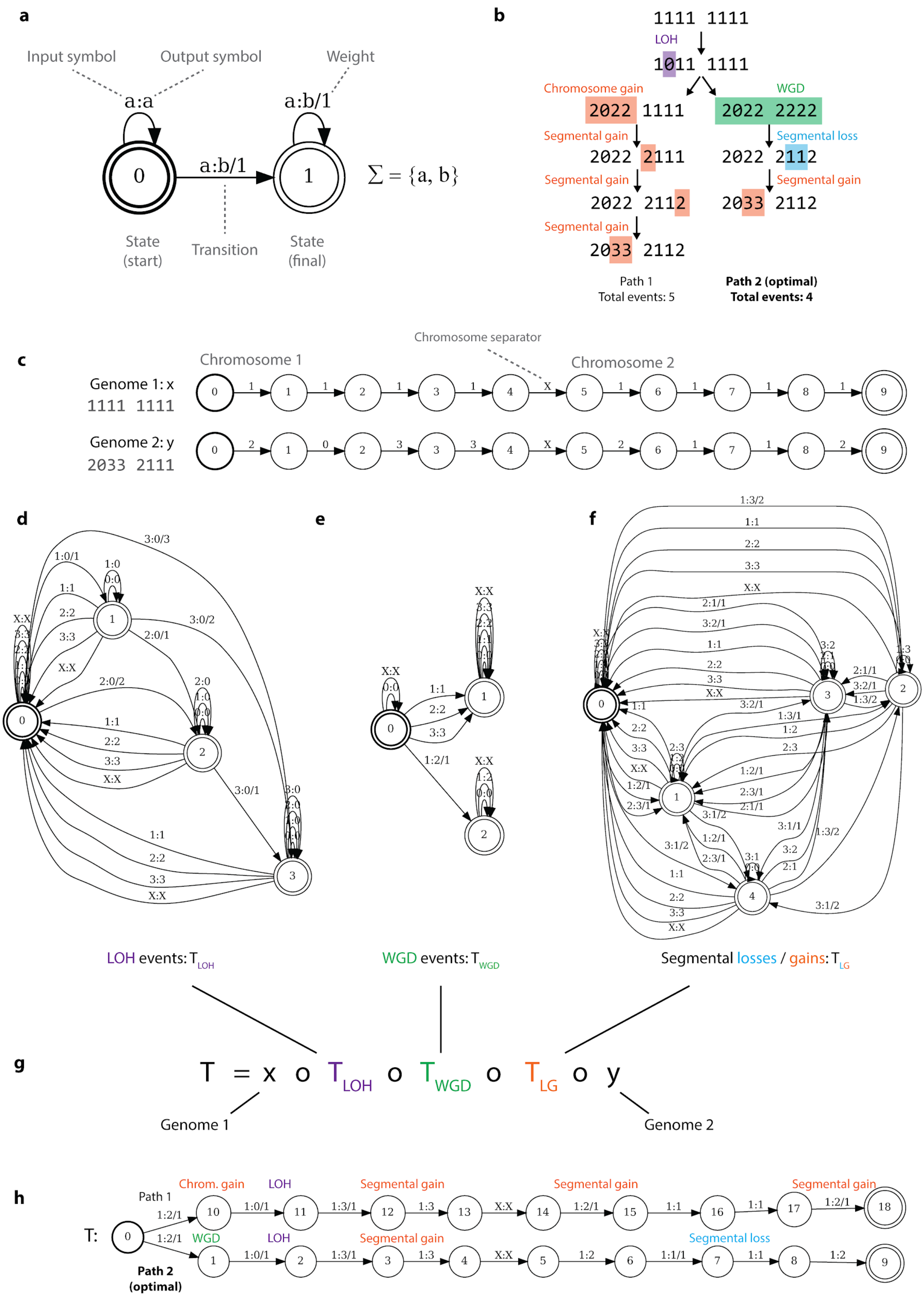

Fig. S1: Exemplary construction of the MED using the FST framework. a) FSTs describe transformations of strings over an alphabet Sigma (here {a,b}). They consist of states (circles) and transitions (arrows). Transitions are equipped with an input symbol followed by a colon and an output symbol, as well as a weight or cost of taking that transition indicated after the slash ("/"). **b)** The running example from Figure 1b has two different paths, one including a WGD event, and one without, both of which lead to the desired final copy-number profile. However, the path which includes the WGD event takes one event less (MED=4) compared to the one without the WGD event (MED=5), and will be preferred. **c)** Copy-number profiles / genomes are represented as acceptors, i.e. unweighted finite-state machines where each transition only contains a single output symbol. Chromosome boundaries are marked with chromosome separator character "X". **d-f)** Separate FSTs describe transformations for LOH events (d), WGD events (e) and other segmental gains and losses of arbitrary length (f). **g)** These FSTs are composed with both genomes into the final asymmetric MED FST "T", the shortest path in which identifies the minimally necessary series of events (MED).

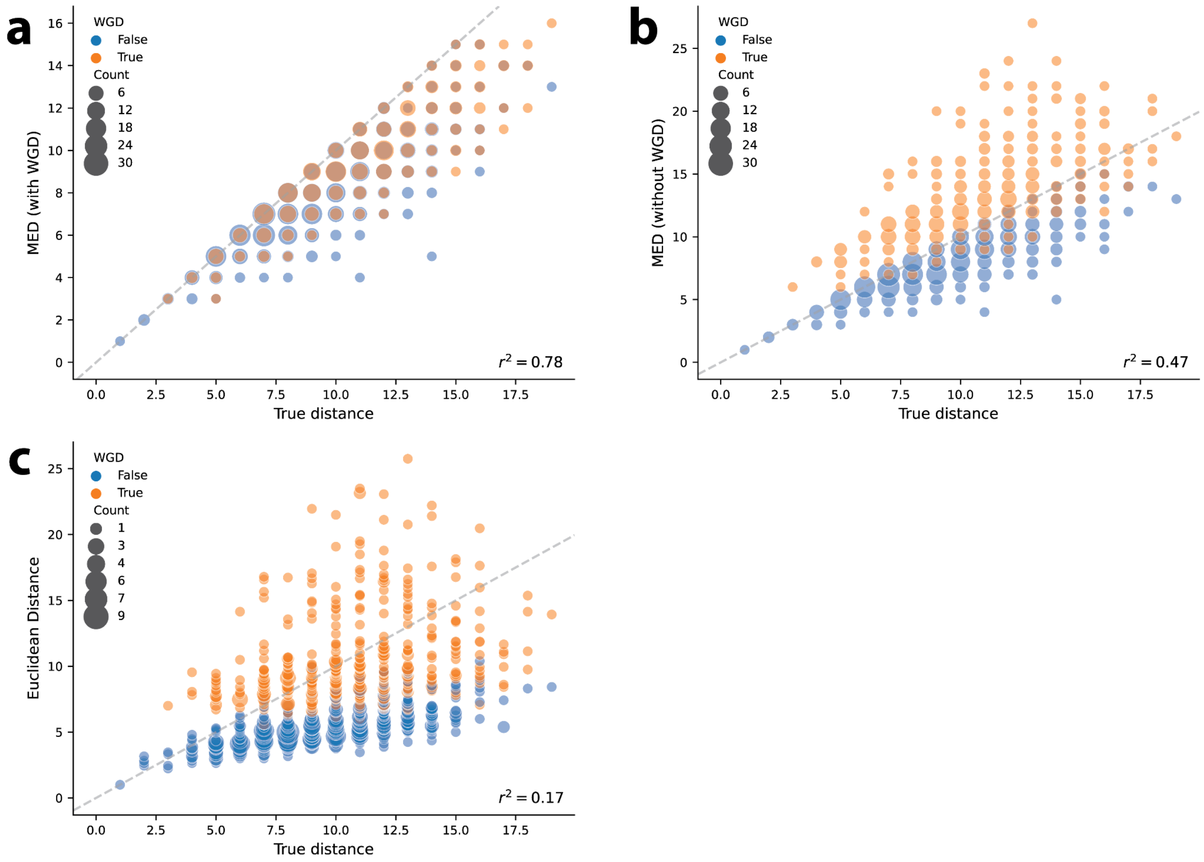

Fig. S2: Model performance on simulated distances

a) Using 1000 random instances of copy-number evolution starting from a diploid genome, MEDICC2 correctly infers distances no greater than the true simulated distance if using MED-WGD (r^2=0.78). **b)** If not taking WGD events into account the MED overestimates distances in sequences where WGD events have occurred (orange). **c)** Euclidean distance is unable to recover the actual tree distance in profiles with and without WGD events.

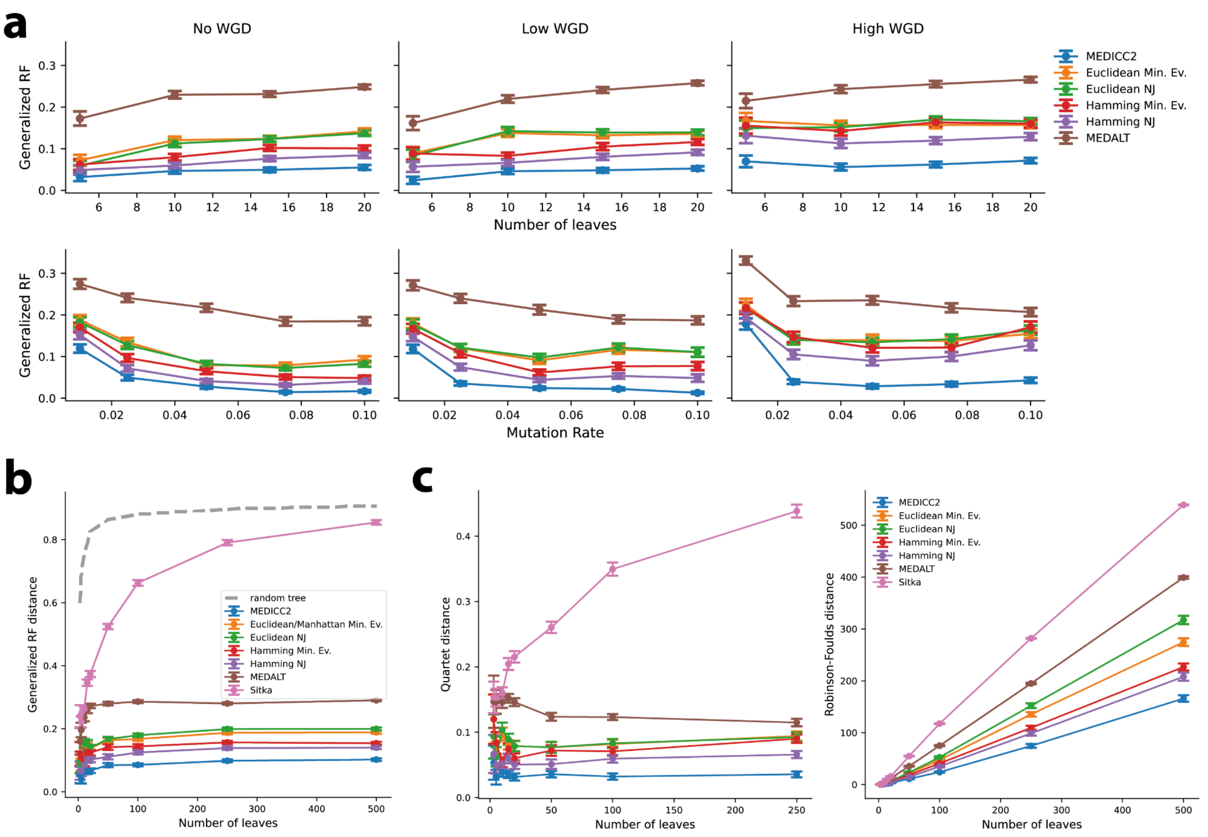

Fig. S3: Model performance on simulated trees.

a) Results of the tree reconstruction for a range of intermediate tree sizes, mutation rates and WGD rates. b) Results for the phylogenetic tree reconstruction method Sitka which uses a Markov chain Monte Carlo method for creating the tree and therefore performs poorly on the simulated trees. **c)** MEDICC2 outperforms all other methods also for two other, widely-used tree

distance measures: the Quartet distance and the Robinson-Foulds distance. Note that the Quartet distance cannot be calculated above 250 leaves due to limitations in the library used. Error bars in all figures correspond to the standard error across all runs.

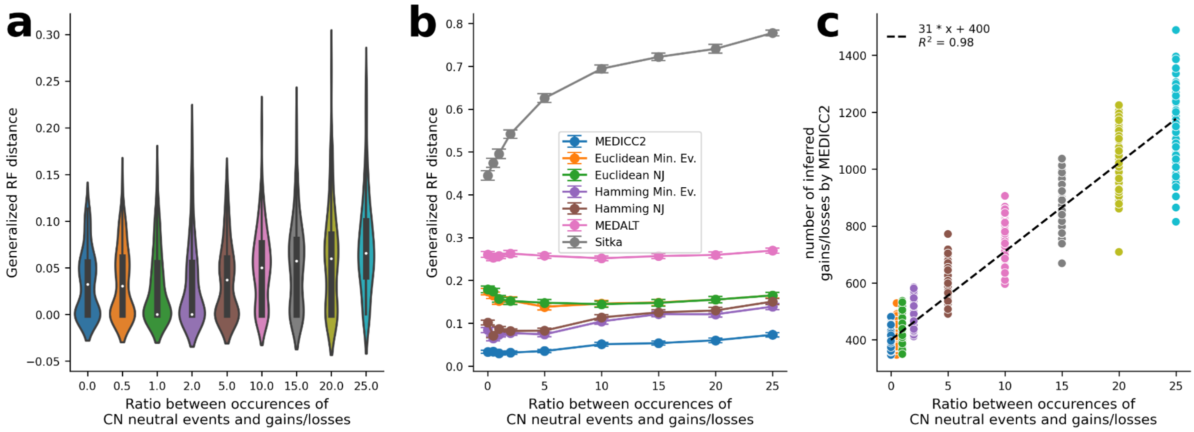

Fig. S4: Effect of translocations and inversions on MEDICC2's performance.

Translocations/inversions will violate the assumption that copy-number events are contiguous with respect to the reference genome. Simulations with an increasing number of translocations/inversions over a fixed amount of gains and losses (both focal and chromosome wide) for 20 leaves and a mutation rate of 0.05 show that: **a)** MEDICC2's reconstruction error increases only slightly with increased translocations and inversions. **b)** For all ratios, MEDICC2 still outperforms all other methods. Breakpoint-based methods such as Sitka seem most strongly affected by translocations and inversions. Error bars correspond to standard errors. **c)** The number of inferred gains and losses increases linearly with the increasing presence of translocations and inversions. A linear model with a fixed intercept of 400 (the number of expected gains/losses), shows a slope of 31 +- 1. For example, a ratio of 10, which leads to 4,000 additional translocations and inversions, leads to MEDICC2 inferring an additional 310 gains and losses on average.

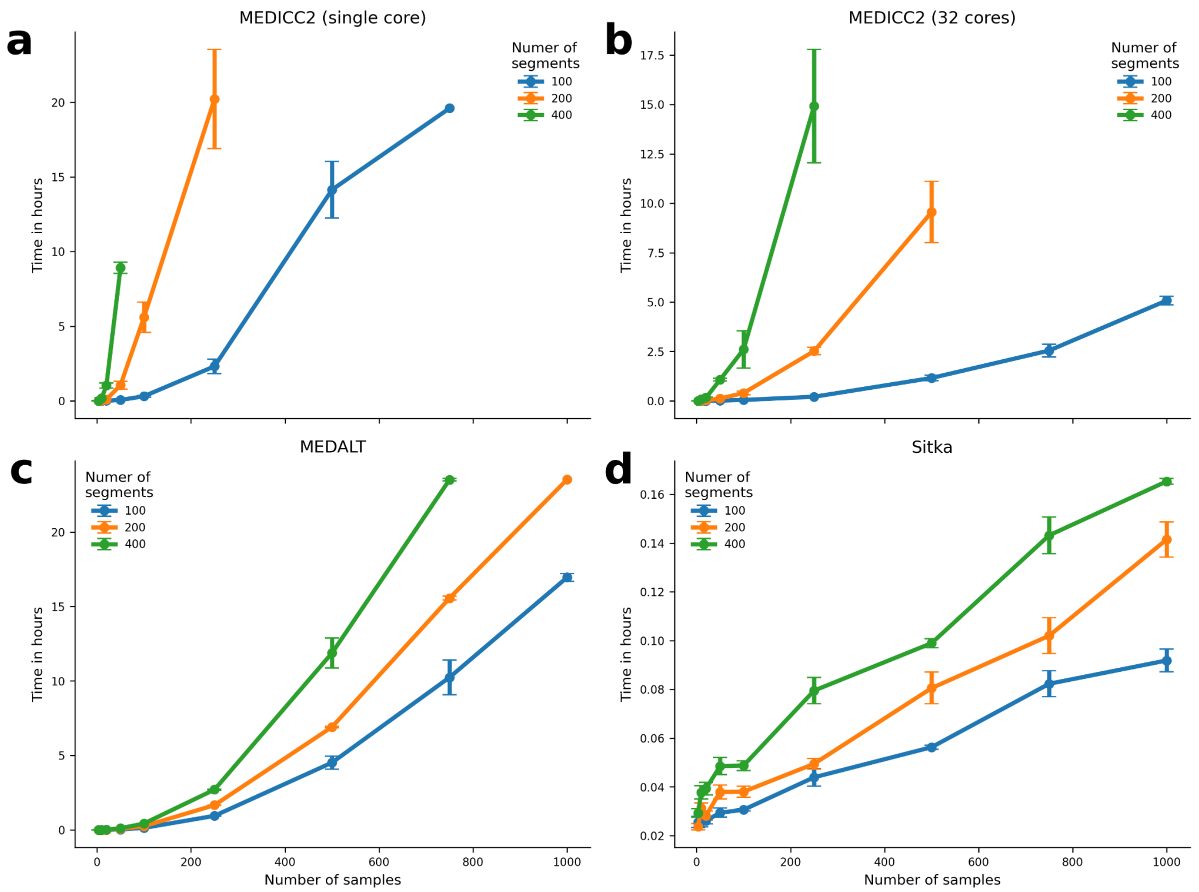

Fig. S5: Performance on simulated data.

The overall runtime of MEDICC2 is quadratic in the number of samples. MEDALT's total runtime is also quadratic in the number of samples but is less strongly affected by the number of segments since it is not computing the symmetric edit distance via a common ancestor. Sitka has a linear runtime relationship to the number of samples. The data was simulated using our simulation framework with a mutation rate of 0.05 and 100, 200 and 400 segments with 5 independent simulations per datapoint. Inferences that took longer than 24 hours were omitted and error bars represent standard errors between the 5 runs. Some data points do not have an error bar because 4 out of the 5 runs took longer than 24 hours, for example MEDICC2 (single core) 750 samples with 100 segments.

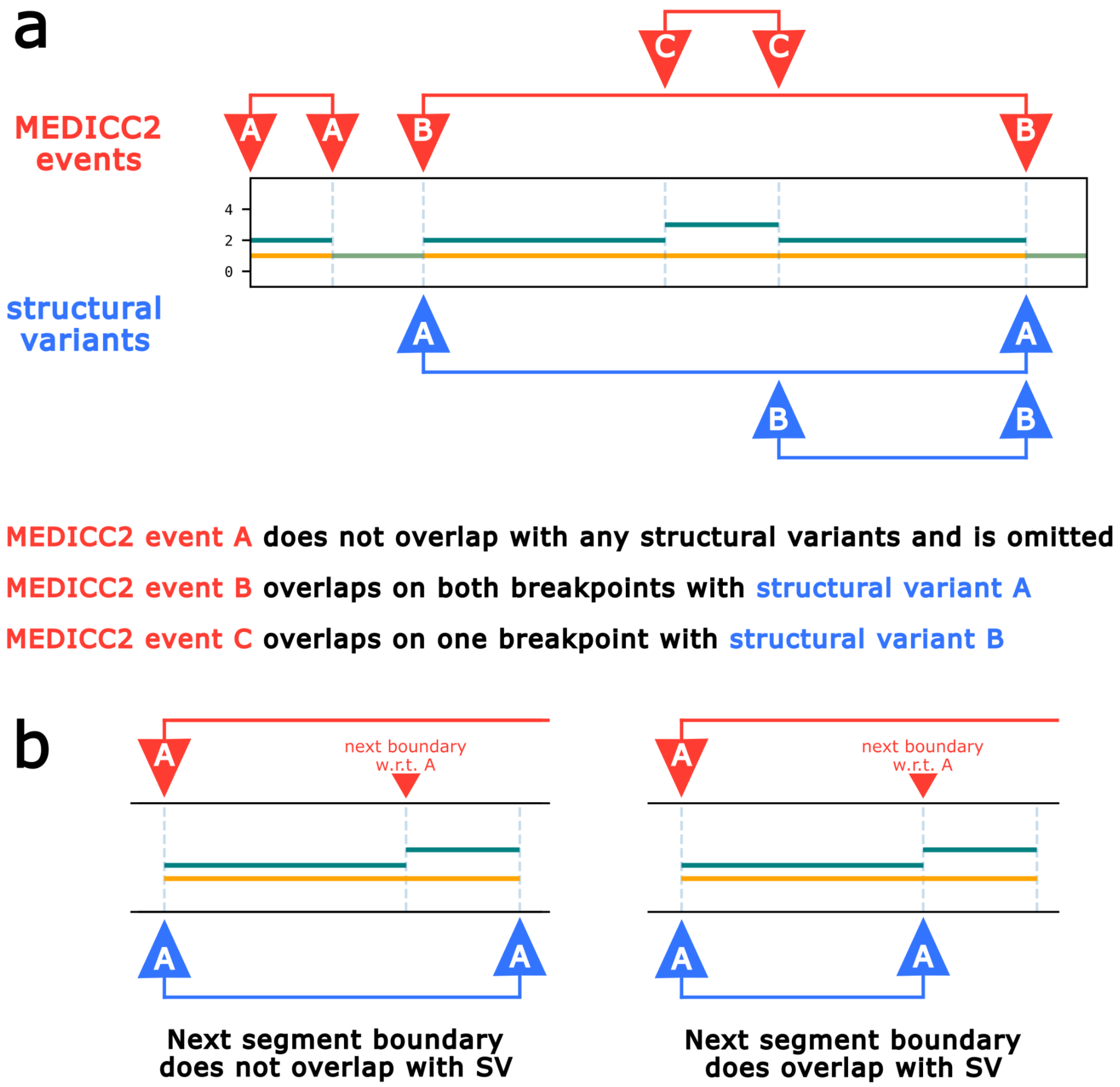

Fig. S6: Explanation of the structural variant validation routine.

a) In order to select MEDICC2 events and structural variants for the validation routine we checked for all MEDICC2 events that have at least one overlap with a structural variant. MEDICC2 event A does not overlap with any structural variant and is therefore omitted from the analysis. MEDICC2 event B overlaps with the structural variant B on both breakpoints whereas MEDICC2 event C only overlaps on one side. **b)** We compared MEDICC2 to a null model that just considers the next segment boundary. In the left example, the next segment boundary does not overlap with the structural variant whereas in the right example it does.

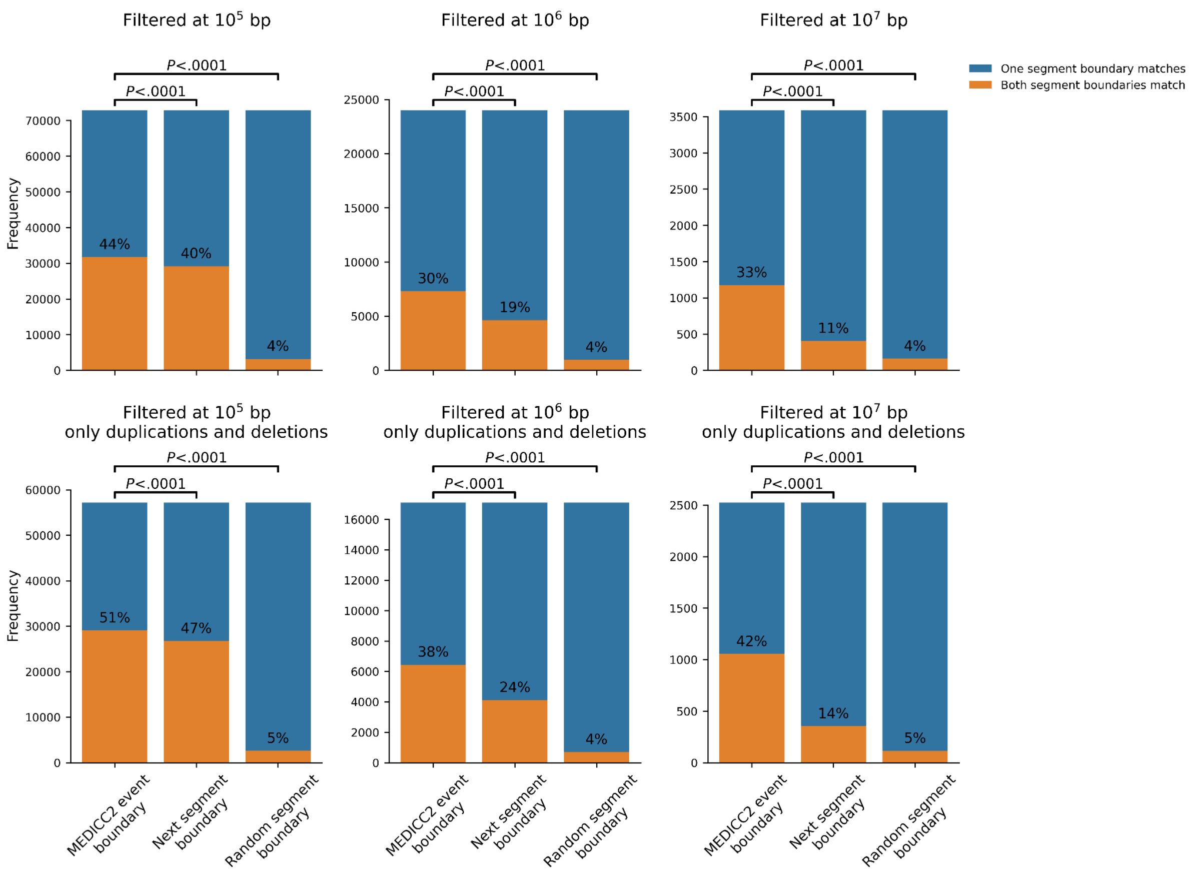

Fig. S7: Model validation using structural variants. Pairs of MEDICC2 events and SVs were chosen based on an overlap of the starting segment. We assume MEDICC2 events to be supported by the SV if the ends also overlap. SVs were filtered based on their type (only duplications and deletions - bottom row - or all types - top row) and on their size.

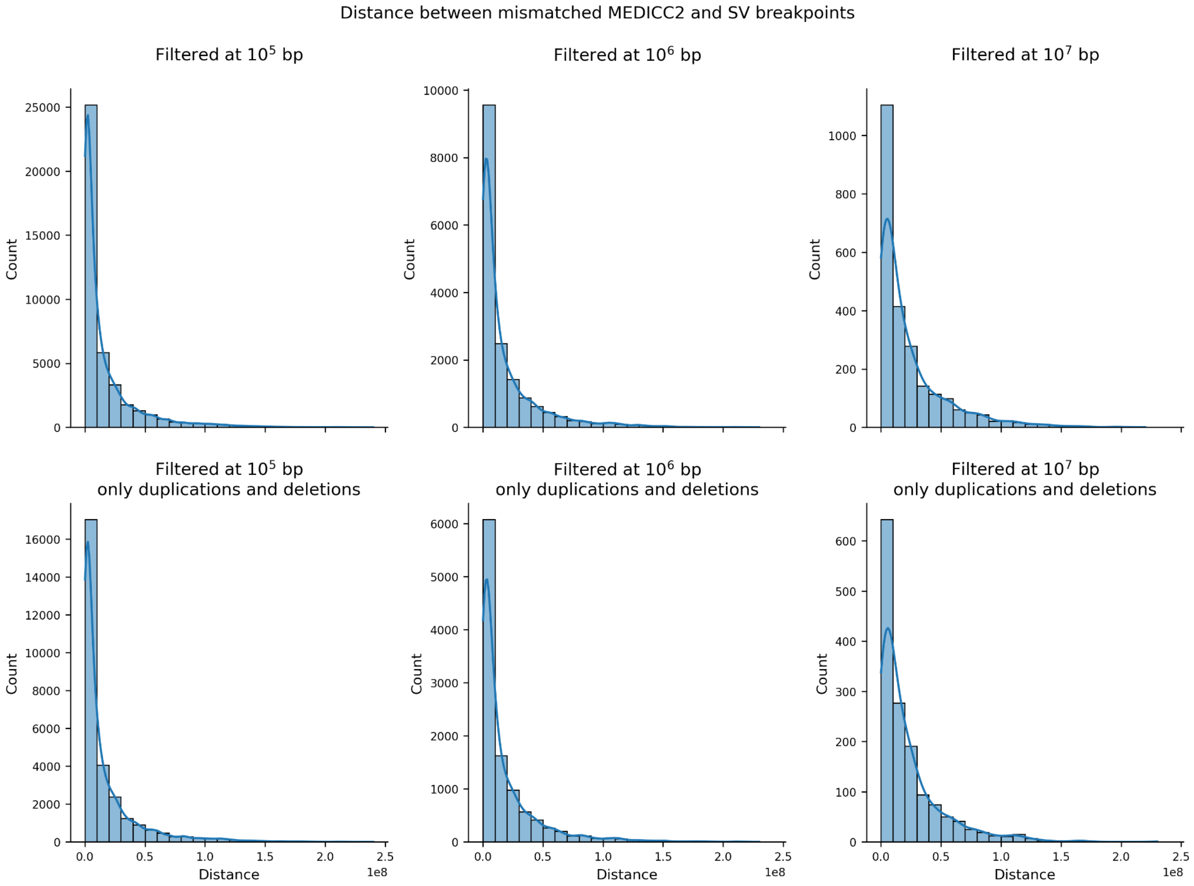

Fig. S8: Model validation using structural variants, distance of mismatched breakpoints. In the SV validation analysis, for every mismatched breakpoint (labelled as “One segment boundary matches” in Fig. S7) we measured the distance (in Mb) between the mismatched MEDICC2 and SV breakpoints. For all filters, more than 40% of all mismatched MEDICC2 breakpoints were within 10Mb of the correct SV breakpoint.

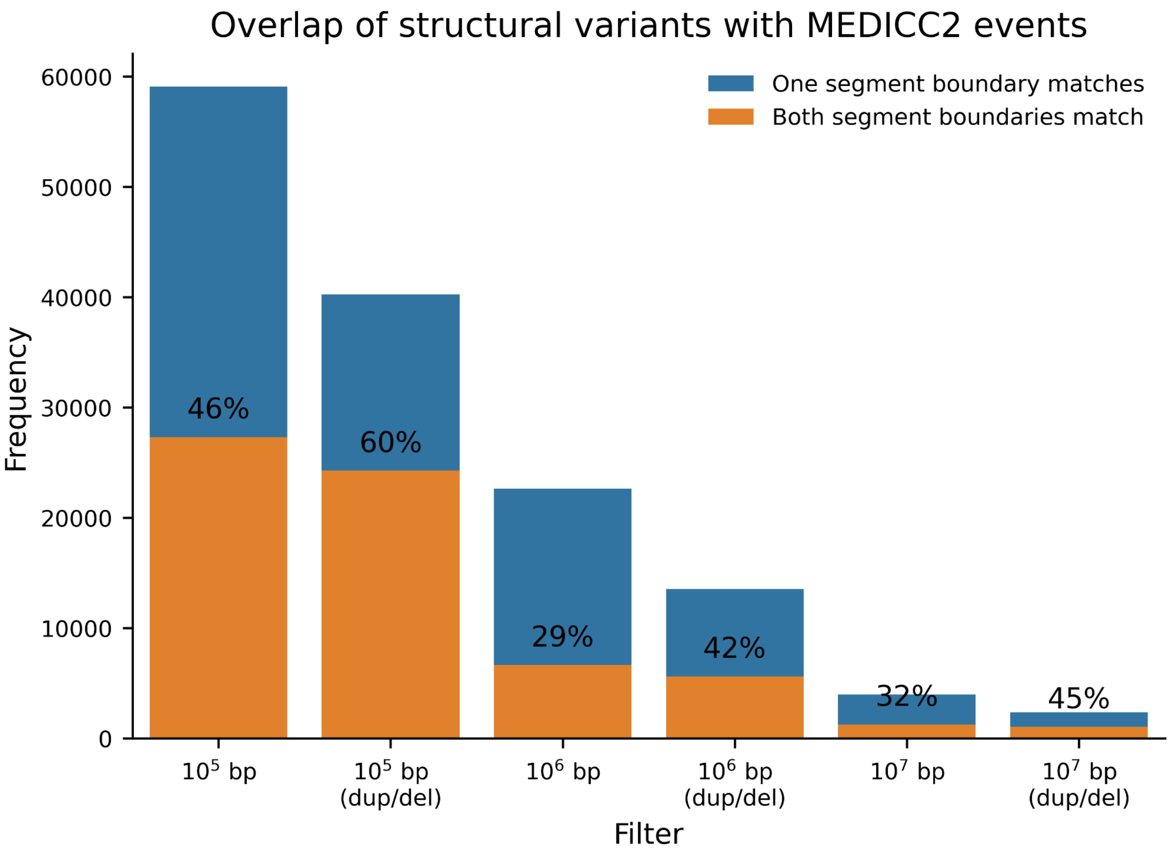

Fig. S9: Overlap of structural variants with MEDICC2 events. Repeating the SV validation analysis from the perspective of the SVs. To this end, we select all SVs that overlap on at least one breakpoint with a MEDICC2 event (marked as “One segment boundary matches”) and check which percentage of those also overlap on the matching second breakpoint (“Both segment boundaries match”). Note that due to symmetry in the analysis, the number of SVs in the “Both segment boundaries match” category is the same as the number of MEDICC2 events in that category. The number of MEDICC2 events and SVs with label “One segment boundary matches” is not the same because multiple MEDICC2 events can overlap with a single SV and vice versa.

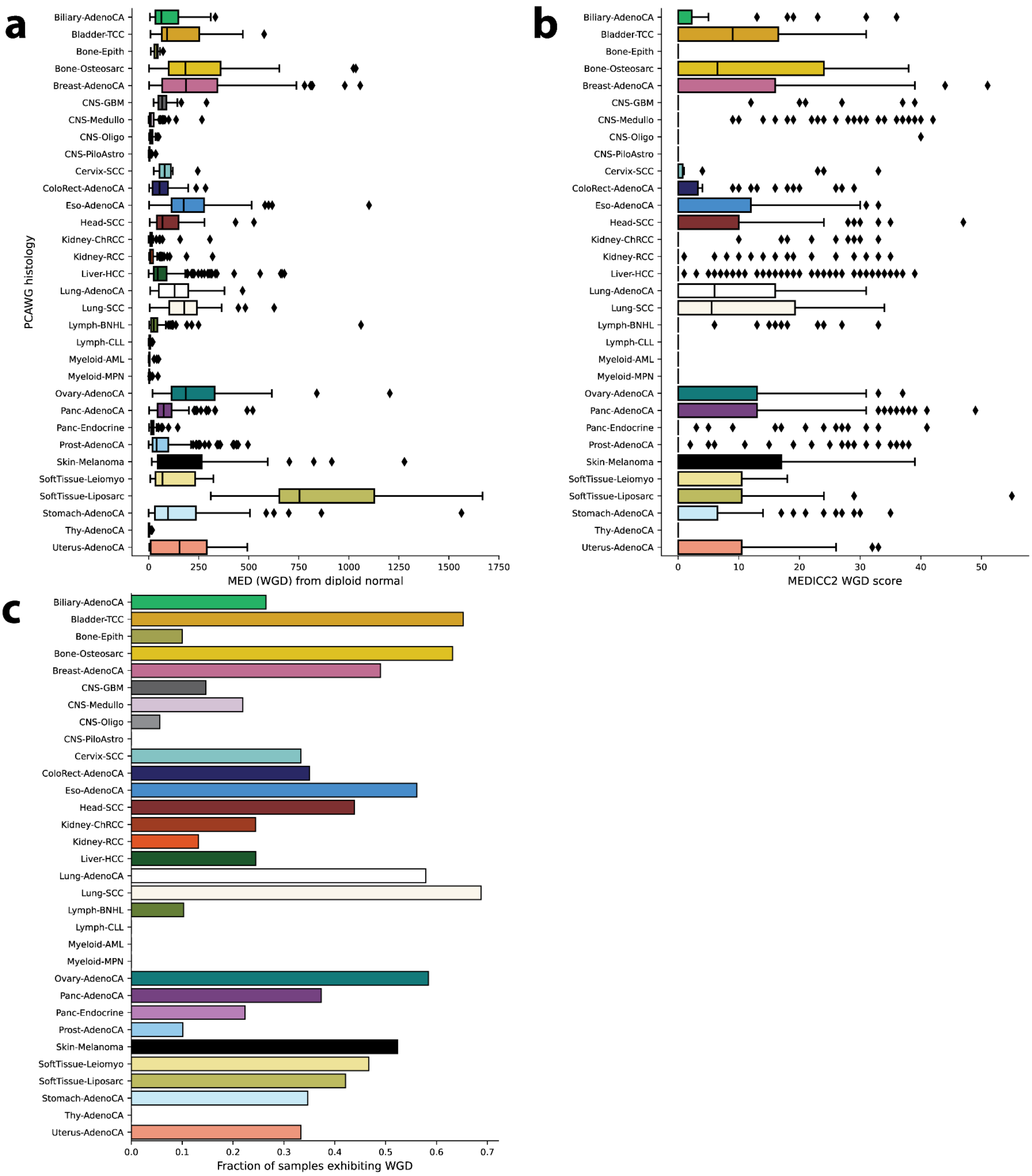

Fig. S10: Overview of the PCAWG cohort.

a) MEDICC2 MED-WGD for all PCAWG tumours show differing levels of copy-number evolution. **b) MEDICC2 WGD score for all PCAWG tumours** and **c) Fraction of samples exhibiting WGD** show varying degrees of WGD events across the cohort. Boxplots in *a* and *b* show the quartile of the distributions with whiskers extending to fit all data points that are not outliers based on the interquartile range.

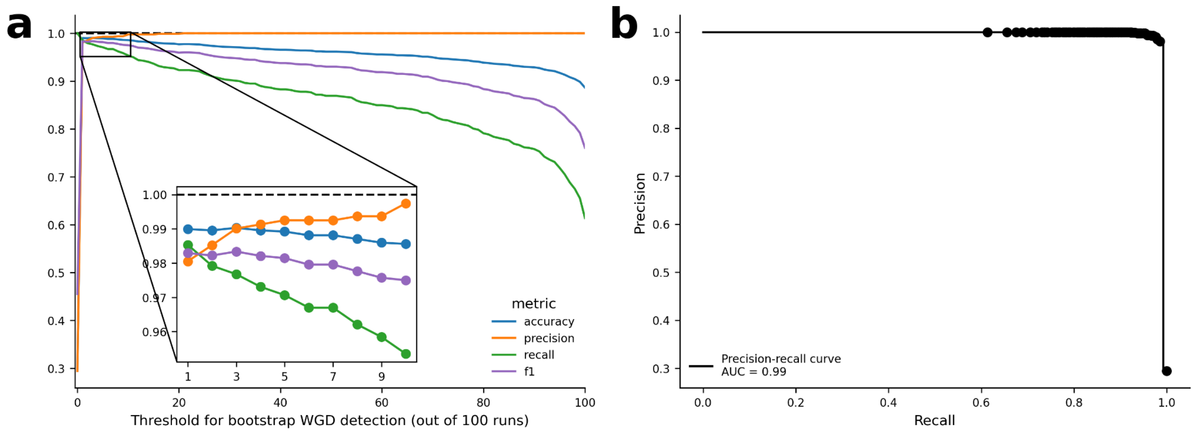

Fig. S11: Effect of the bootstrap percentage threshold for the bootstrap WGD detection.

a) Accuracy, precision, recall and f1 score for all possible values of the bootstrap percentage threshold for WGD detection. As expected, the recall decreases with increasing threshold while the precision rises (reaching a value of 1.0 for a threshold of 20%). The accuracy and f1 score both reach their maximum value (0.990 and 0.983 respectively) for a threshold of 3%. Both values decrease after this peak. However, both accuracy and f1 score stay reasonably high for threshold values <=10%. **b)** Precision-recall curve for the MEDICC2 bootstrap WGD detection with an area under the curve of 0.99.

Supplementary Figures 12 - 20:

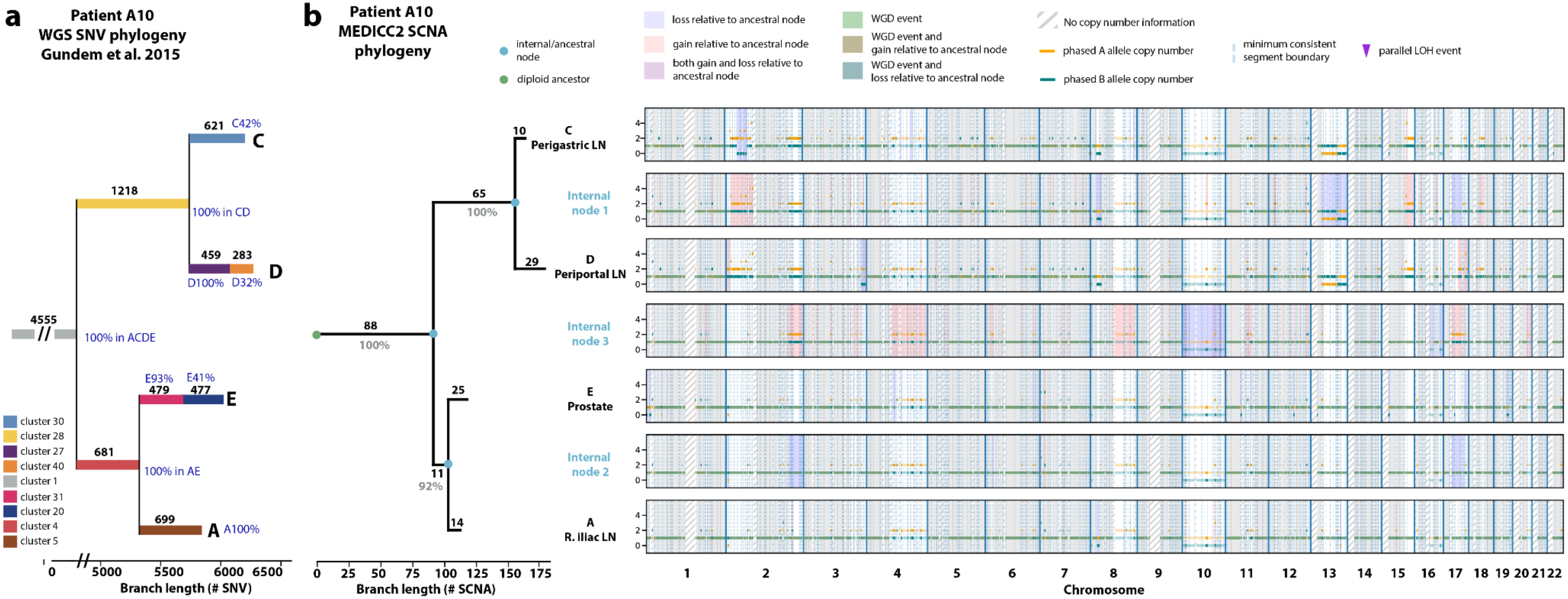

Fig. S12: Evolutionary history of tumour subclones from patient A10.

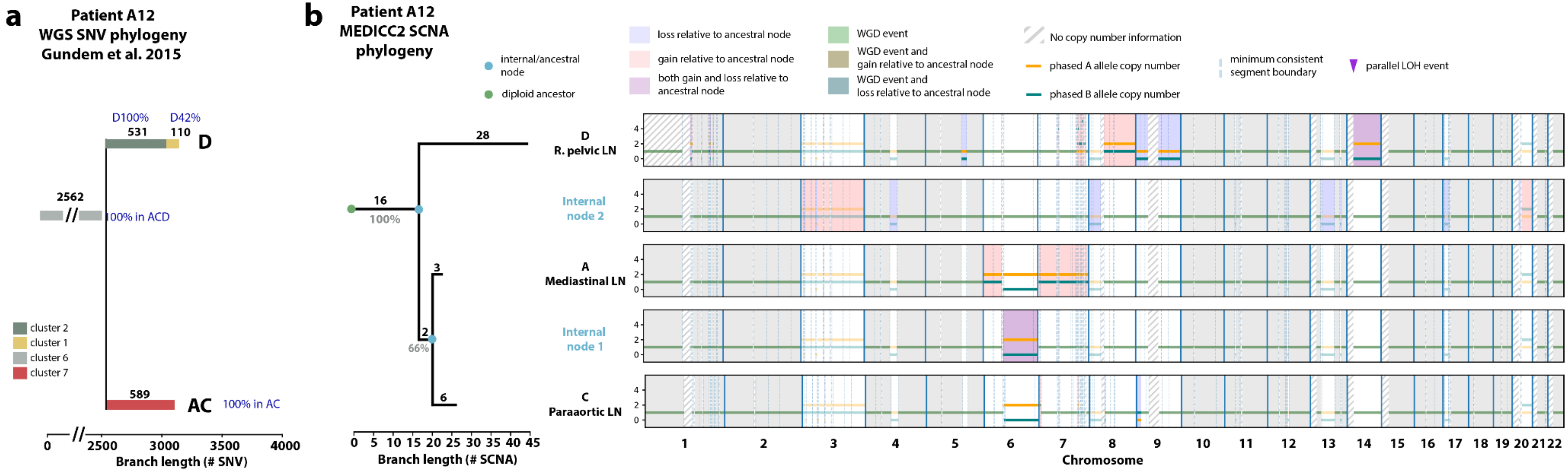

Fig. S13: Evolutionary history of tumour subclones from patient A12.

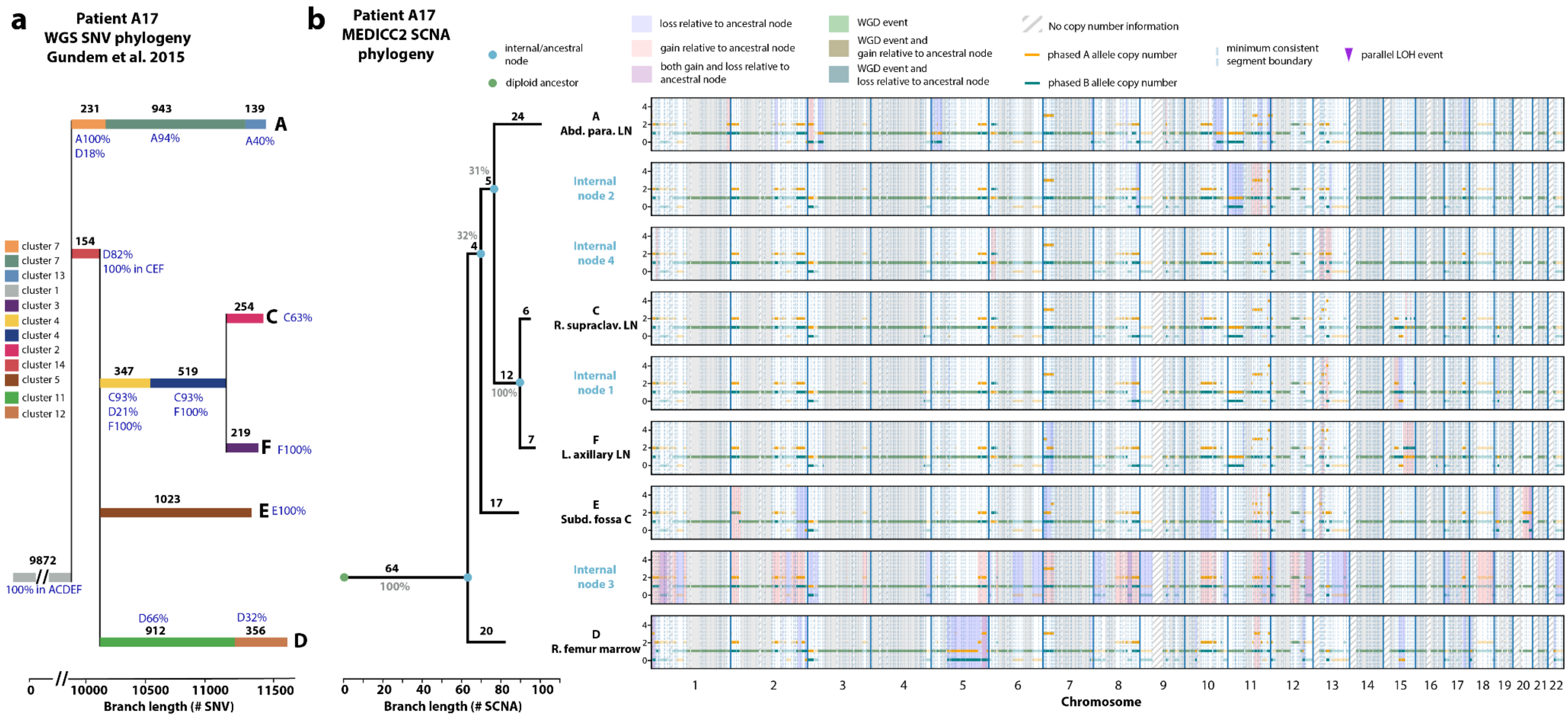

Fig. S14: Evolutionary history of tumour subclones from patient A17.

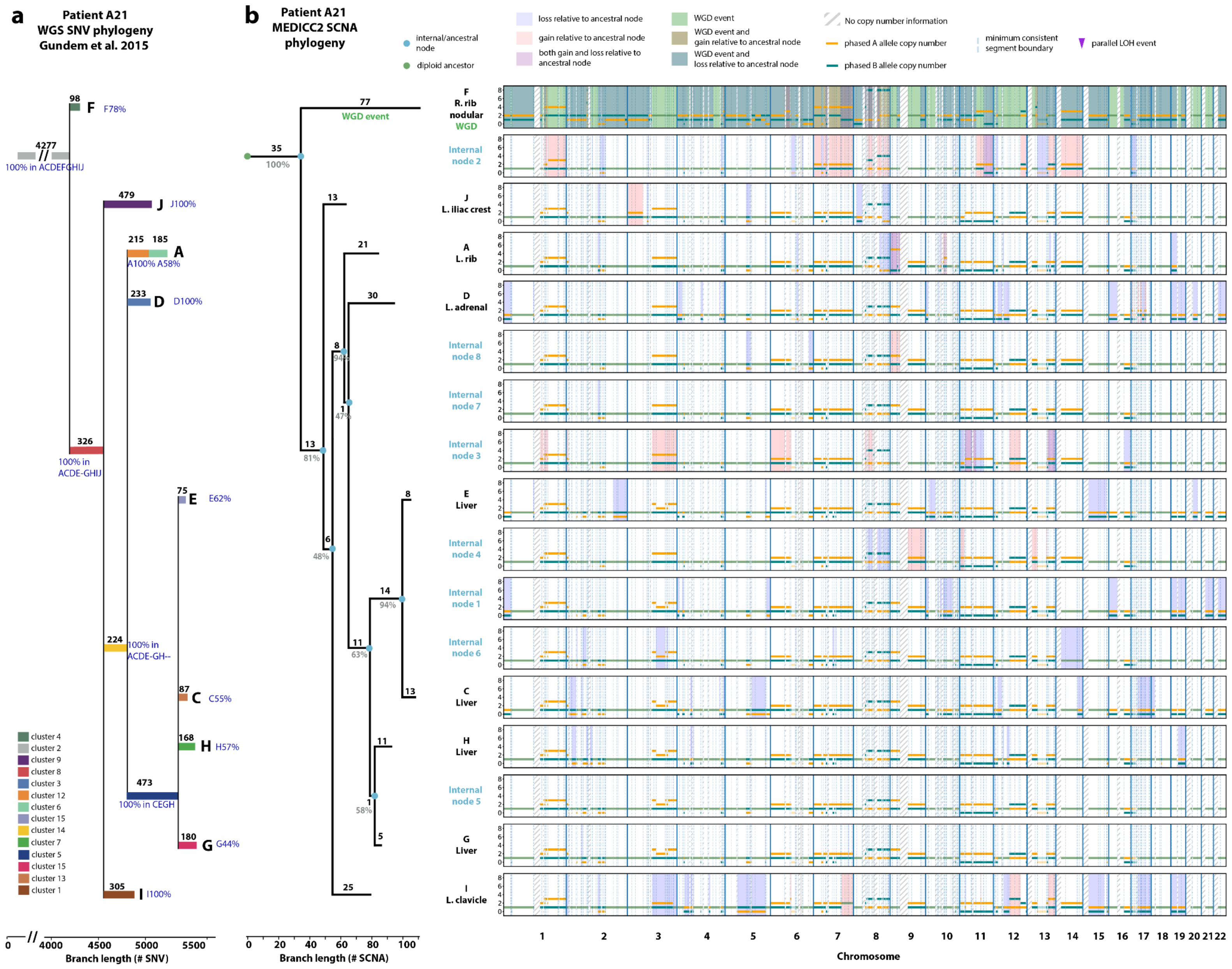

Fig. S15: Evolutionary history of tumour subclones from patient A21.

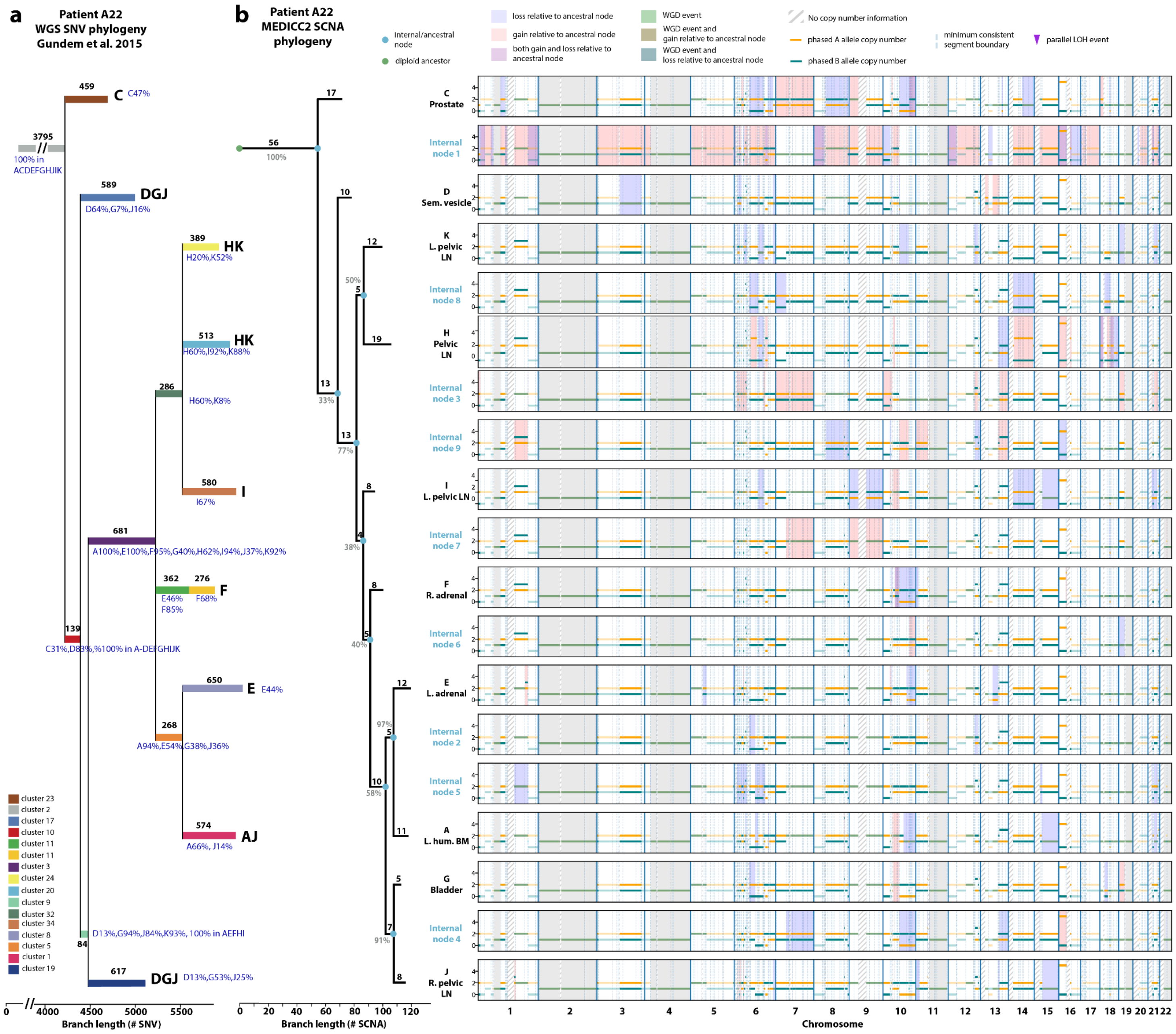

Fig. S16: Evolutionary history of tumour subclones from patient A22.

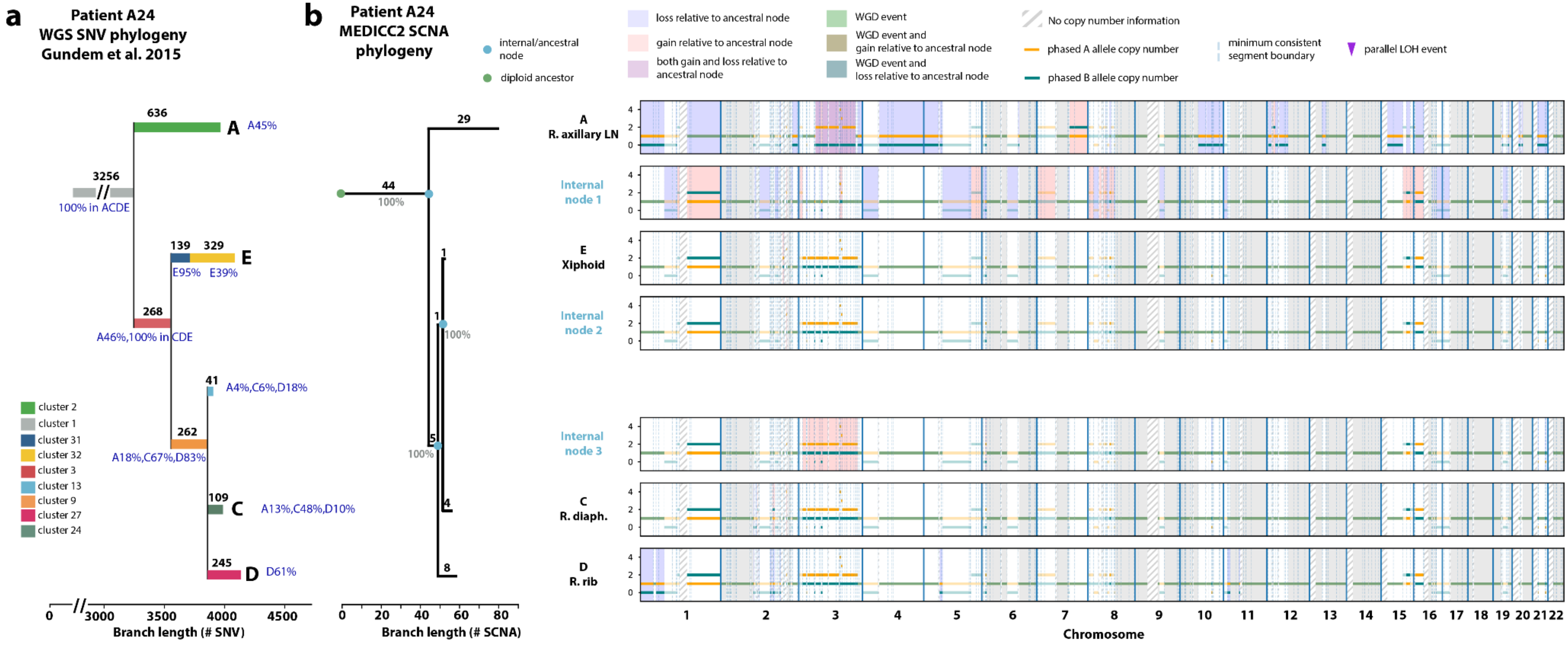

Fig. S17: Evolutionary history of tumour subclones from patient A24.

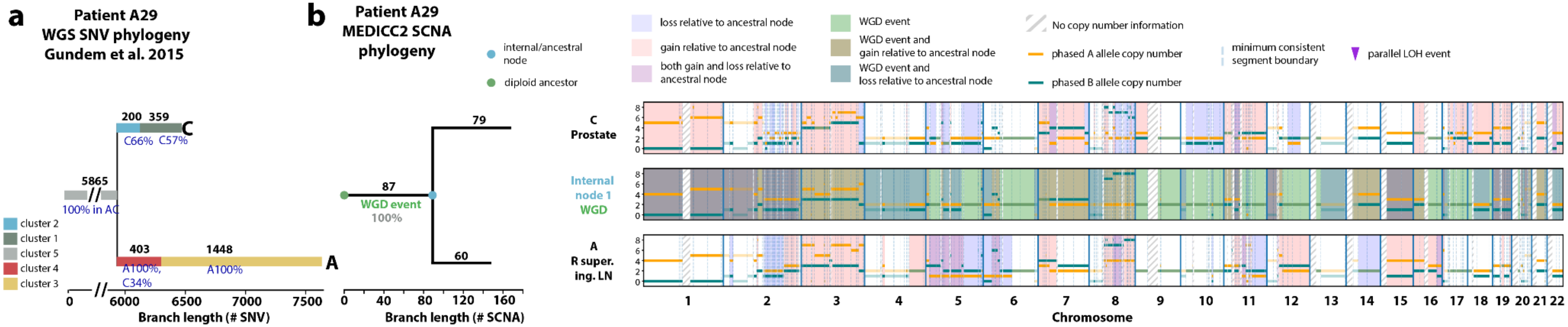

Fig. S18: Evolutionary history of tumour subclones from patient A29.

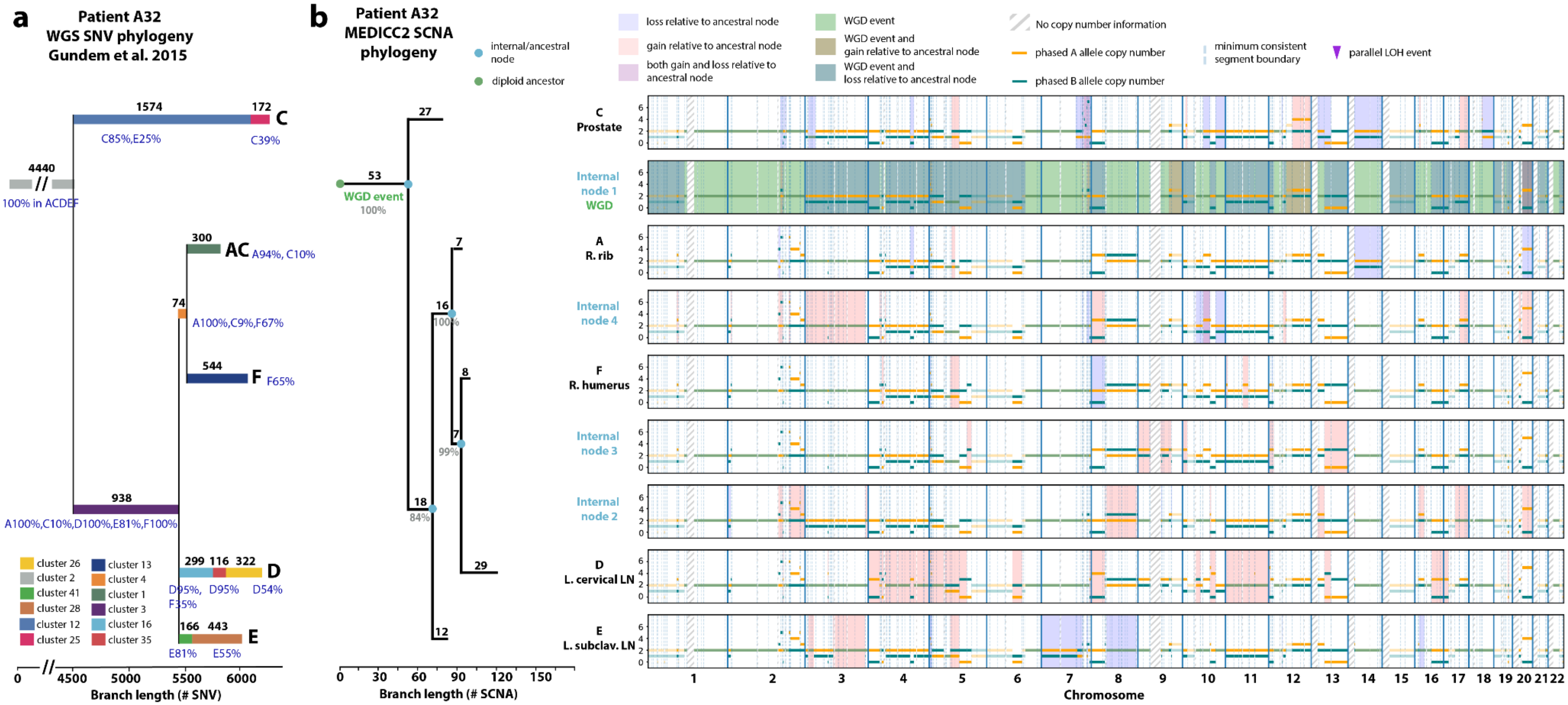

Fig. S19: Evolutionary history of tumour subclones from patient A32.

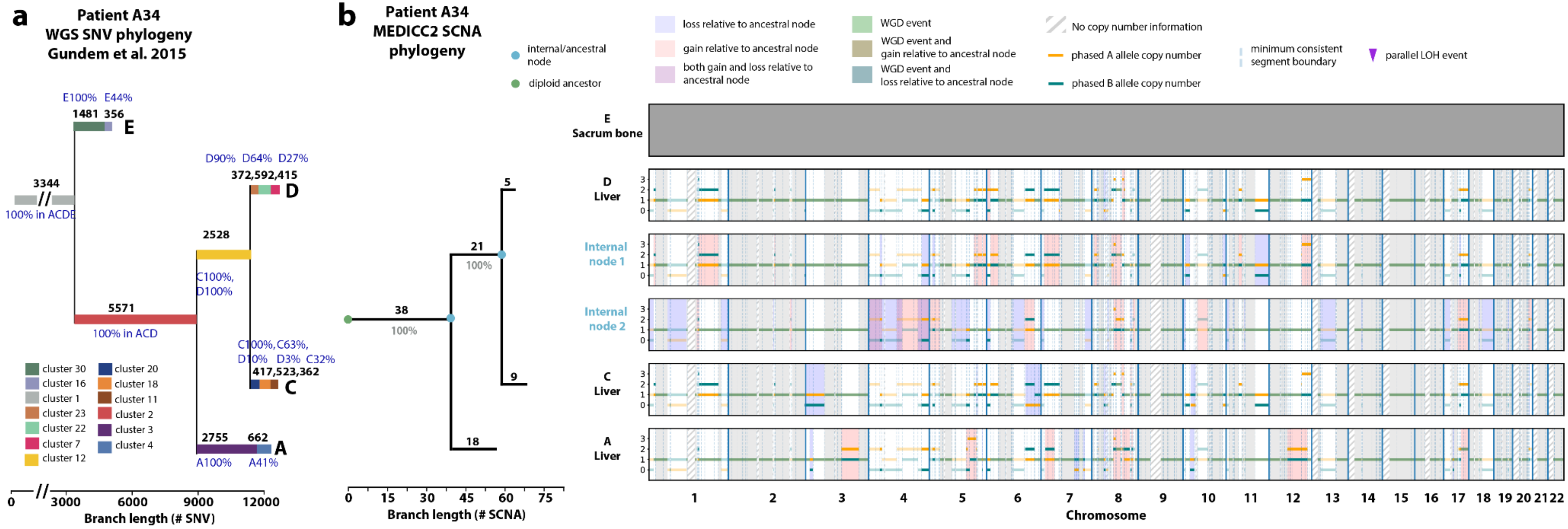

Fig. S20: Evolutionary history of tumour subclones from patient A34.

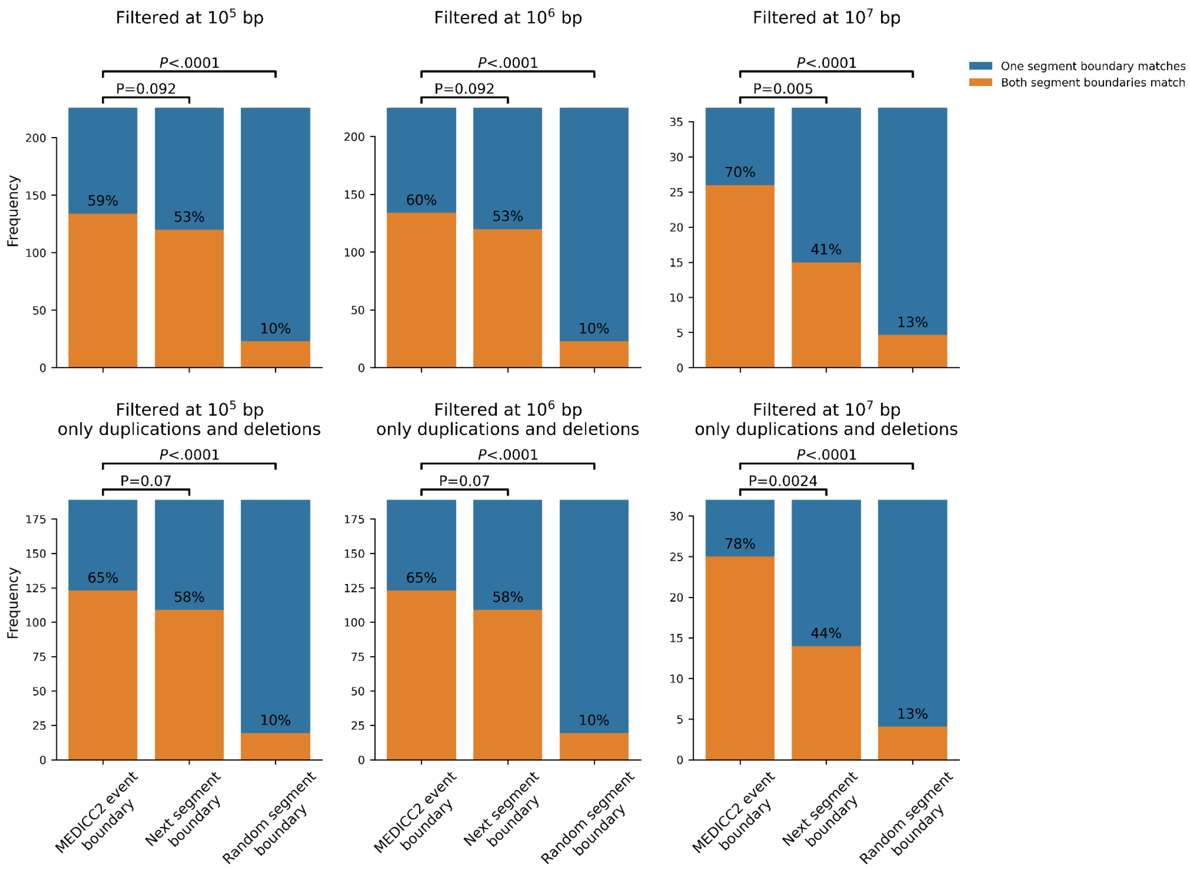

Fig. S21: Model validation using SVs on multi-sample prostate cancer data. We repeated the SV validation analysis for the 10 patients from the Gundem et al. cohort. Despite the limited number of samples (50 in total) we find a strong overlap of MEDICC2 events with SVs similar to the single-sample data (Fig. S7).

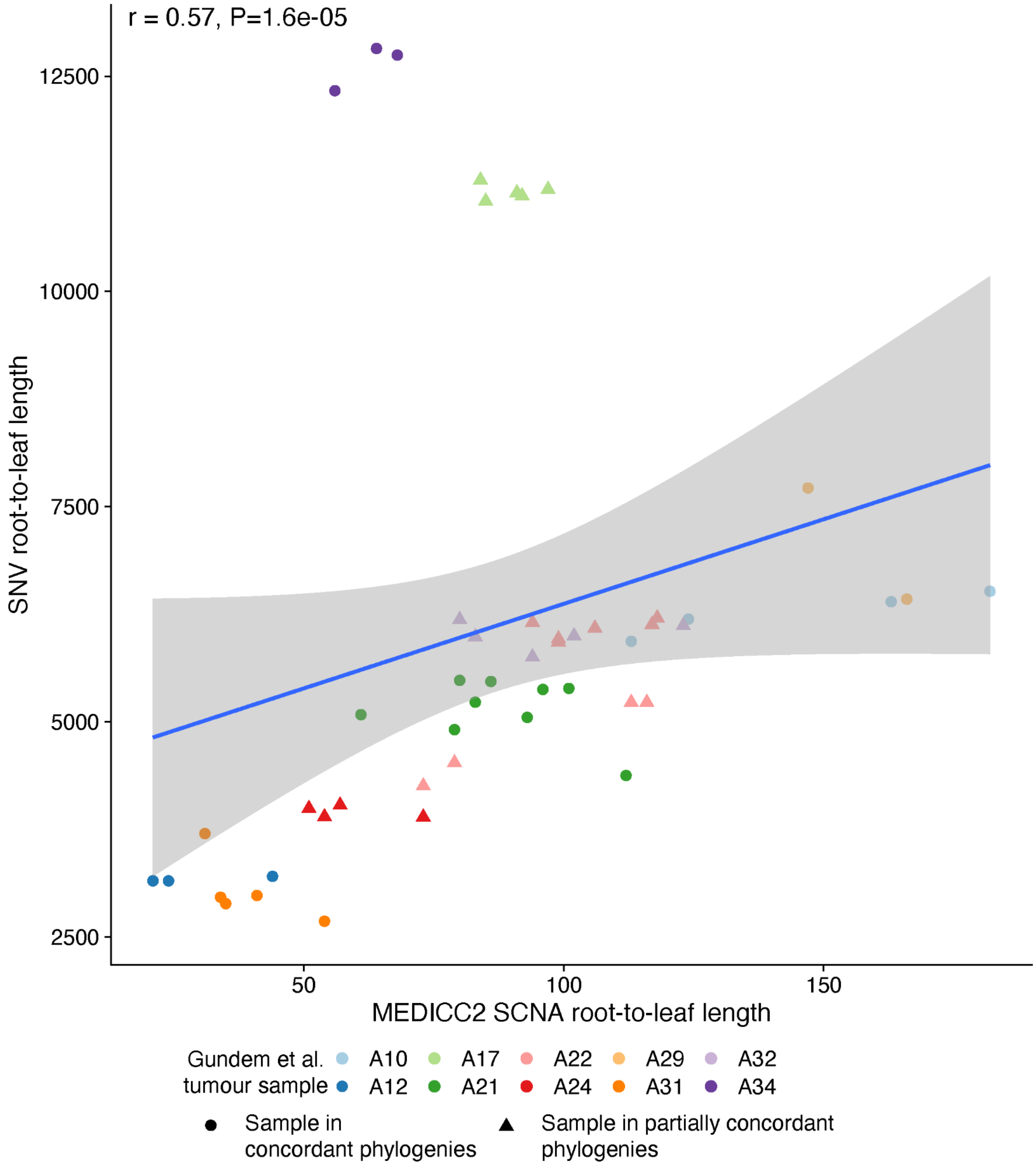

Fig. S22: Comparison of root-to-leaf distances across the cohort

Scatterplot showing the absolute root-to-leaf lengths for samples from the MEDICC2 phylogenies and SNV-based clone phylogenies reproduced from Gundem et al. Data points are coloured by the tumour the samples are from and their shape corresponds to whether the MEDICC2 and SNV-based clone phylogenies demonstrated concordance (circles) or partial concordance (triangles) as defined by the Robinson-Foulds distance. The grey shaded area represents the 95% confidence interval. ρ and P values are from a Spearman correlation test.

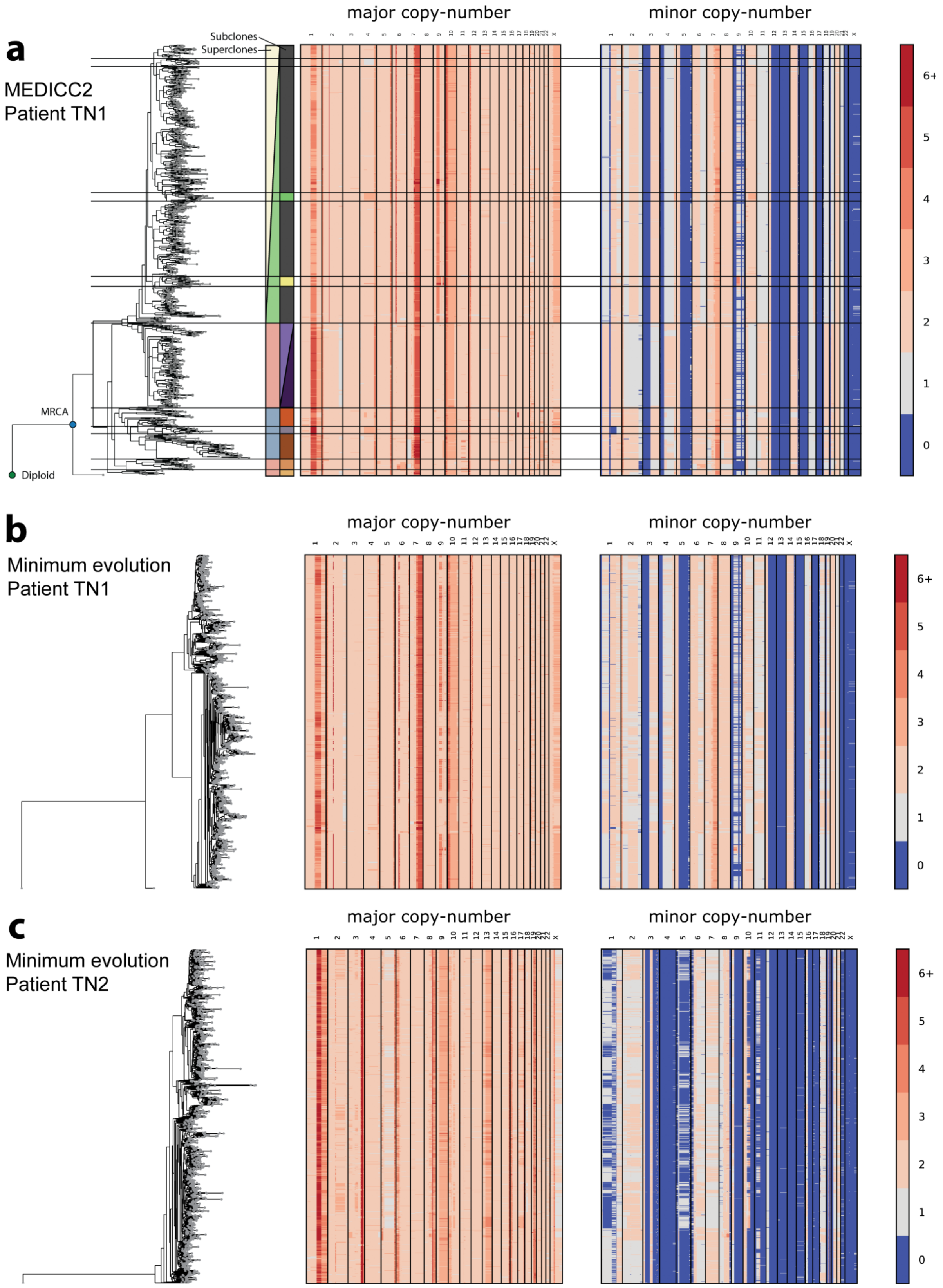

Fig. S23: Single-cell cohort from Minussi et al. 2021.

a) Inferred phylogeny and allele-specific copy-number profiles for patient TN1 from Minussi et al.

2021. Superclones and subclones are marked as in the original publication. **b)** and **c)** show the inferred phylogenies using the Manhattan-distance based minimum evolution tree as described in the original publication. Unlike the MEDICC2 trees, the Manhattan trees do not recover the super- and subclone structure.

Supplementary Tables

	**Reconstruction error (Generalised RF distance)**			**Runtime (in hours)**			
**Number of leaves**	**MEDICC 2**	**MEDALT**	**Sitka**	**MEDICC 2**	**MEDICC 2 (32 cores)**	**MEDALT**	**Sitka**
**3**	0.00	0.12	0.12	0.00	0.00	0.00	0.02
**10**	0.03	0.23	0.26	0.01	0.01	0.00	0.03
**20**	0.04	0.25	0.44	0.09	0.02	0.01	0.03
**50**	0.05	0.26	0.62	1.05	0.12	0.06	0.04
**100**	0.06	0.27	0.76	5.60	0.40	0.26	0.04
**250**	0.07	0.27	0.87	20.22	2.52	1.67	0.05
**500**	0.07	0.28	0.92	>24	9.55	6.91	0.08

Table S1: Comparison of runtime and reconstruction accuracy between MEDICC2, MEDALT and Sitka.

The data was simulated using our simulation framework with a mutation rate of 0.05 and 200 total segments. Each entry is the mean value from 25 runs for the reconstruction error and 5 runs for the runtime.

patient	# samples	# segments	time in seconds (average of 5 runs)
**A10**	5	622	19.7
**A12**	4	143	2.3
**A17**	6	606	46.0
**A21**	10	340	113.7
**A22**	11	255	33.8
**A24**	5	254	9.9
**A29**	3	271	3.1
**A31**	6	142	4.2
**A32**	6	249	9.7
**A34**	4	288	6.0

Table S2: Performance for the Gundem et al. 2015 dataset.

The analysis was performed on a single-core personal computer with intel core i-7 vPro (8th generation) processor.

**patient**	**# cores**	**time in hours (average of 5 runs)**
**TN1 (1100 samples, 81 segments)**	1	5.6
	2	2.4
	4	1.4
	8	0.7
	16	0.6
	32	0.5
**TN2 (1023 samples, 107 segments)**	1	9.7
	2	4.9
	4	2.7
	8	1.8
	16	1.2
	32	1.0

Table S3: Performance for the Minussi et al. 2015 dataset with varying number of cores used. The analysis was performed on a high computing cluster with CPUs from the Intel Xeon Processor E5 family.
