# Supplementary material for: MEDICC2: whole-genome doubling aware copy-number phylogenies for cancer evolution
Source: Genome Biol. 2022 Nov 14;23:241. doi: 10.1186/s13059-022-02794-9 (PMC9661799; doi:10.1186/s13059-022-02794-9)
Supplement: Supplementary file 2 — Additional file 2. Peer review history. [file 13059_2022_2794_MOESM2_ESM.docx]

**Review history**

**First round of review**

**Reviewer 1**

The authors tackle the problem of tumor phylogeny inference based on copy-number aberrations. While there are a number of tumor phylogenetic methods available, many of them make restrictive assumptions about evolution that are not applicable to copy-number aberrations. CNA evolutionary models are challenging because events are large, complex and frequently overlapping, introducing correlation and making distance measurements challenging. The authors introduce MEDICC2, an algorithm based on an event based model to tackle this problem. MEDICC2 is a considerable advance over both MEDICC and other existing work in the space, allowing it to accurately model parallel evolution, WGD events (which are highly common and present a significant confounder to copy number models) and uses a steiner tree formulation (instead of a minimum spanning tree).

I found this manuscript to be well written and impressive. The methodology is well motivated and addresses an important problem in cancer phylogenetics. The methods introduce non-trivial algorithmic advances and are described in a way that should be approachable to a broader audience. The software itself is available open-source, is well-documented and easy to install (through package managers conda and pip, as well as directly from source), and already being used by the cancer genomics community. I make some specific comments below, but overall I think this is a valuable contribution and was a pleasure to read.

Major comments

The main thing that would greatly strengthen this paper for me is a discussion/evaluation of the trade-offs in evolutionary model accuracy/complexity in this space, and where MEDICC2 lies on the spectrum. This model is neither the least nor the most complex model studied for copy-number/genomic rearrangement evolution and there are tradeoffs for moving up and down the complexity ladder. The manuscript in its present form compares against "simpler" methods but doesn't address the computational cost of its added complexity. Likewise, it doesn't directly highlight the ways that the model itself makes simplifications (e.g., events are contiguous with respect to the reference genome, an assumption that may no longer be valid when considering events overlapping with novel somatic adjacencies). I don't believe these are true limitations of the method - as the saying goes, "All models are wrong, but some are useful". But I do think this isn't well understood by people outside of a very small group and would greatly contextualize this work.

Specifically, runtime isn't directly reported in the manuscript (authors do note the O(N^2) scaling complexity, but this is mentioned deep in the methods, and doesn't give a sense of the constants involved). In our own experience running the software, MEDICC2 is considerably slower than multiple other tools in the space and running on large single cell datasets requires considerable computational resources. Showing runtimes of different tools (e.g. on simulated data) would contextualize the tradeoff in accuracy results and be useful for potential users of the software.

How limiting is the assumption that events are contiguous with respect to the reference?

Changepoint methods (e.g. , sitka https://doi.org/10.1101/2020.05.06.058180 ) are also interesting in this discussion: the no breakpoint reuse Dollo model is less constricting and likely more biologically valid than the ISA of interval-based methods, but don't consider copy-number magnitude treat breakpoints independently (unlike MEDICC2). As a result, they are potentially (and in my experience) considerably faster, but a head-to-head comparison on accuracy with MEDICC2 would be interesting.

The SV analysis is interesting but I'm confused as to what "next" or "nearest" copy-number boundary means (nearest to the first MEDICC2 breakpoint, the second breakpoint, or nearest to the SV boundary)? Also, the reported concordance (even for duplications/deletions only) is fairly low (<50%) — while 100% concordance wouldn't be expected, it'd be useful to see this analysis taken further to understand where this divergence is coming from. Currently the reported result is one-sided (how many of MEDICC2's calls show up in the SV data?) but a precision/recall type analysis would yield more insight (how many SV supported duplication/deletions are identified by MEDICC2?). Likewise are the patterns in differences? - e.g., is this jitter (the SV boundary is just out of the 100kb proximity)? Are boundary breakpoints mismatched (that is, each breakpoint overlaps with an SV boundary but not the same event)?

For the bulk analysis if A31 - are samples assumed to be homogeneous? How valid is this assumption (with respect to these specific samples) and could violations of this assumption potentially confound results, especially with regards to the conclusion of parallel events?

Minor Comments:
The model section in methods would greatly benefit from a figure (even supplementary).

"Validation dataset" and "Overlap of MEDICC2 events with structural variants" mentions the data of "individual cells" when discussing PCAWG bulk tumor samples - is this an error?

P.20, line 8: "poisson" -> "Poisson"
P.20, line 19: "The start of an event of gain or loss events in the sequence The start of an event"
P.27, line 21: "The logged ratios Logged ratios"
The paper switches back and forth between "tumor" and "tumour"

**Reviewer 2**

The authors present MEDICC2, a significant update to their previous similarly named method. The new method is able to account for whole genome duplication, uses a new lazy method for efficient computation of the FST score, and is able to reconstruct ancestral allele specific copy number states. The authors show broad utility by applying their data to the problem of WGD detection in single sample WGS, and phylogenetic reconstruction in multi-sample WGS and single cell low pass WGS. The software implementing the method is high quality and easy to use. I list my major and minor concerns below.

Major:

1. Page 4 line 29, the authors should emphasize that the calculation of MED is linear in the length of the sequence. It would be helpful to have a more thorough breakdown of the time complexity relative to number of cells/samples and max copy number.

2. In figure 5, why is chromosome 5 minor allele copy number heterogeneous in the clones at the bottom half of the phylogeny. The q arm of chromosome 5 is inferred to be lost producing LOH many times throughout the phylogeny. Is there a solution for which this region is lost once? Would some other region then be inferred to be recurrently gained or lost for such a solution? How are the authors sure of the presented solution over a solution for which 5q is lost only once? Alternatively, are some of the calls of loss or lack of loss related to the noisiness of the data?

3. The authors limited comparison with MEDALT, a minimum spanning tree based method, could be improved. The authors do not specify how they convert the spanning tree to a phylogeny for comparison with MEDICC2 results. A reasonable approach would be to take all internal node samples/cells and place them on leaves connected to the internal node by a zero length branch. Is something like this what was done? Can the authors provide more insight into the differences between the MEDICC2 and MEDALT solutions and why MEDALT performs so poorly?

4. The WGD results on page 8 are somewhat confusing. What is the breakdown of PCAWG WGD annotations for the 2778 cases? As written it sounds as if all 2778 are WGD.

5. Page 8, line 20, how many of the 110 were annotated as WGD uncertain?

6. Page 8, line 27, 5% of bootstrapped samples is a very low bar for calling WGD. What threshold is actually needed to provide a significant boost in detection accuracy?

7. The authors apply MEDICC2 to the Gundem et al., for which subclonality at sampled sites like makes sample level phylogenies less informative for certain patients. For A31 it appears that a sample phylogeny is equivalent to the clone phylogeny. Can the same be said for the data from the other patients? How much of an issue is the subclones and their presence / mixing across multiple sites?

8. Page 19, line 14, the authors normalize by the maximum root to leaf distance. Was this done independently per left (sample) or was the entire tree normalized by the longest branch in the tree? The SNV / SCNA branches appear to be locally correlated (dependent on normalization) with the exception of the trunk, the branch to E (burst of complex SCNA) and the branch to C (driver SNVs?). The text here could be a little more clear and care should be taken with describing the normalization. Possibly an independent normalization (based on the cohort?) would better expose the branches with expected ratio of SNV to SCNA vs those that have unexpected differences in rates?

Minor:

1. Page 6, line 60, "counting genomic segments" is ambiguous, counting the number of copies of each segment?

2. Page 8, line 9, "predicted to have undergone two WGD events" … please specify if these events were parallel or consecutive

3. Page 8, line 32, did you mean supp figure 4 instead of supp figure 14?

4. For figure 5, i assume the left is total copy number and the right is minor copy number but i couldnt find labels or info from the figure legend.

**Authors’ response to reviewers**

Reviewer reports:

Reviewer #1

The authors tackle the problem of tumor phylogeny inference based on copy-number aberrations. While there are a number of tumor phylogenetic methods available, many of them make restrictive assumptions about evolution that are not applicable to copy-number aberrations. CNA evolutionary models are challenging because events are large, complex and frequently overlapping, introducing correlation and making distance measurements challenging. The authors introduce MEDICC2, an algorithm based on an event based model to tackle this problem. MEDICC2 is a considerable advance over both MEDICC and other existing work in the space, allowing it to accurately model parallel evolution, WGD events (which are highly common and present a significant confounder to copy number models) and uses a steiner tree formulation (instead of a minimum spanning tree).

I found this manuscript to be well written and impressive. The methodology is well motivated and addresses an important problem in cancer phylogenetics. The methods introduce non-trivial algorithmic advances and are described in a way that should be approachable to a broader audience. The software itself is available open-source, is well-documented and easy to install (through package managers conda and pip, as well as directly from source), and already being used by the cancer genomics community. I make some specific comments below, but overall I think this is a valuable contribution and was a pleasure to read.

Major comments

1) The main thing that would greatly strengthen this paper for me is a discussion/evaluation of the trade-offs in evolutionary model accuracy/complexity in this space, and where MEDICC2 lies on the spectrum. This model is neither the least nor the most complex model studied for copy-number/genomic rearrangement evolution and there are tradeoffs for moving up and down the complexity ladder. The manuscript in its present form compares against "simpler" methods but doesn't address the computational cost of its added complexity. Likewise, it doesn't directly highlight the ways that the model itself makes simplifications (e.g., events are contiguous with respect to the reference genome, an assumption that may no longer be valid when considering events overlapping with novel somatic adjacencies). I don't believe these are true limitations of the method - as the saying goes, "All models are wrong, but some are useful". But I do think this isn't well understood by people outside of a very small group and would greatly contextualize this work.

Authors’ response: We thank the referee for their helpful comments, and agree that greater efforts could have been made to place our findings within the context of the field. We have attempted to illustrate the varying complexity of approaches taken by others and to better contextualise our methodology and its impact on MEDICC2’s runtime compared to other tools.

In order to achieve this we have rewritten the Discussion section of the manuscript, elucidating the most commonly used simplifications in the field of cancer phylogenetics, and have given examples of methods which employ these simplifications. We have further expanded the Discussion to include a more detailed section about the ramifications of the contiguity assumption implicit to MEDICC2. To investigate this in more detail we have conducted systematic simulations which assess the effect of translocations and inversions violating the contiguity assumption on tree reconstruction accuracy (in line with your comment #3). We have also expanded our comparison on simulated data to include Sitka (in line with your comment #4) and have added Supplementary Table 1 and Supplementary Figure 5 which allow side-by-side comparison of the runtime/complexity and reconstruction accuracy of all tools.

For the simulation approach we used the same framework that was also used for the reconstruction accuracy benchmarking and simulated a wide range of trees with between 3 and 1000 samples and between 100 and 400 segments. Generally speaking, running MEDICC2 on data with ≤25 samples is feasible in a few minutes on a personal computer without extensive computational resources. However, in the case of large-scale single-cell datasets, as the calculation of evolutionary distances scales with O(N^2), we recommend the use of MEDICC2’s parallelization capabilities by running it with multiple cores.

The results show that MEDALT’s total runtime is also quadratic in the number of samples but is less strongly affected by the number of segments since it is not computing the symmetric edit distance via a common ancestor. Sitka has a linear relationship to the number of samples, but also performs worst of all compared tools.


Supplementary Figure 5: Performance on simulated data.
The overall runtime of MEDICC2 is quadratic in the number of samples. MEDALT’s total runtime is also quadratic in the number of samples but is less strongly affected by the number of segments since it is not computing the symmetric edit distance via a common ancestor. Sitka has a linear runtime relationship to the number of samples. The data was simulated using our simulation framework with a mutation rate of 0.05 and 100, 200 and 400 segments with 5 independent simulations per datapoint. Inferences that took longer than 24 hours were omitted and error bars represent standard errors between the 5 runs. Some data points do not have an error bar because 4 out of the 5 runs took longer than 24 hours, for example MEDICC2 (single core) 750 samples with 100 segments.

Supplementary Table 1: Comparison of runtime and reconstruction accuracy between MEDICC2, MEDALT and Sitka.
The data was simulated using our simulation framework with a mutation rate of 0.05 and 200 total segments. Each entry is the mean value from 25 runs for the reconstruction error and 5 runs for the runtime.


2) Specifically, runtime isn't directly reported in the manuscript (authors do note the O(N^2) scaling complexity, but this is mentioned deep in the methods, and doesn't give a sense of the constants involved). In our own experience running the software, MEDICC2 is considerably slower than multiple other tools in the space and running on large single cell datasets requires considerable computational resources. Showing runtimes of different tools (e.g. on simulated data) would contextualize the tradeoff in accuracy results and be useful for potential users of the software.

Authors’ response: We would like to thank the reviewer for pointing out the missing information about runtime in the manuscript. For the datasets used in the manuscript (Gundem et al. 2015 and Minussi et al. 2021) the runtime is reported as Supplementary Tables 2 and 3. In the case of the single-cell dataset (Minussi et al. 2021) we have furthermore shown the effect of using multiple cores. We have also systematically assessed the runtime requirements and reconstruction accuracies of MEDICC2, MEDALT and Sitka through simulations to allow side-by-side comparisons. The results are reported at the end of Results Section 1 (“Inferring phylogenies…”), Supplementary Table 1 and Supplementary Figure 5.

Please also refer to our answer to your comment #1 for more details.

3) How limiting is the assumption that events are contiguous with respect to the reference?

Authors’ response: We thank the reviewer for raising an interesting and important point. Some SVs, such as translocations, will violate the contiguity assumption which underlies MEDICC2 and this assumption was not well communicated in the original manuscript. This has now been clarified in the new Discussion section.

We would also like to emphasise that the simulation framework used for benchmarking in the manuscript was chosen deliberately to not contain this assumption. For this, we included inversions and translocations as well as the complex breakage fusion bridges which all work to move segments away from their position in the reference genome. MEDICC2 outperformed all other methods on these simulations and we are confident in its performance on real life data in which the assumption about the contiguity of events is violated.

To explore further how violations of the contiguity assumption affect the performance of MEDICC2 we conducted new simulations in which we systematically increased the ratio of translocations and inversions over gains and losses up to 25 which is a lot higher than what one would expect in real life tumours (see e.g. Li et al. 2020 Nature). We observe that while the reconstruction error of MEDICC2 gradually rises with increased relative occurrence of translocations and inversions, MEDICC2 seems to be highly robust to these changes and still outperforms all other methods even in scenarios with a very high translocation rate. The results of this new simulation are now included in the manuscript as Supplementary Figure 4 (see also below) and referenced in the Results section. We also show them here for your reference.


Supplementary Figure 4: Effect of translocations and inversions on MEDICC2’s performance.
Translocations/inversions will violate the assumption that copy-number events are contiguous with respect to the reference genome. Simulations with an increasing number of translocations/inversions over a fixed amount of gains and losses (both focal and chromosome wide) for 20 leaves and a mutation rate of 0.05 show that: a) MEDICC2’s reconstruction error increases only slightly with increased translocations and inversions. b) For all ratios, MEDICC2 still outperforms all other methods. Breakpoint-based methods such as Sitka seem most strongly affected by translocations and inversions. Error bars correspond to standard errors. c) The number of inferred gains and losses increases linearly with the increasing presence of translocations and inversions. A linear model with a fixed intercept of 400 (the number of expected gains/losses), shows a slope of 31 +- 1. For example, a ratio of 10, which leads to 4,000 additional translocations and inversions, leads to MEDICC2 inferring an additional 310 gains and losses on average.

Looking at the total number of events inferred by MEDICC2, we see that MEDICC2 infers extra gain and loss events due to the presence of the translocations and inversions. This is to be expected as, for example, two segments that are first brought into proximity by a translocation event and subsequently gained together will be counted as two separate gains by MEDICC2. As we expect these translocation and inversion events to happen equally spread out over all samples, we think that they affect the original pairwise distance matrix in the manner of random noise across all samples thereby conserving the relative distance between samples and still resulting in a good reconstruction of the original tree.
By creating a linear fit with a fixed intercept of 400, we get a slope of 30 +- 1 (see panel C). For a ratio of 10, we have the initial 400 gains/losses as well as 4,000 additional translocations and inversions. In order to take these additional 4,000 additional copy-number neutral events into account, MEDICC2 infers an extra 300 gains/loss events.

4) Changepoint methods (e.g. , sitka https://doi.org/10.1101/2020.05.06.058180 ) are also interesting in this discussion: the no breakpoint reuse Dollo model is less constricting and likely more biologically valid than the ISA of interval-based methods, but don't consider copy-number magnitude treat breakpoints independently (unlike MEDICC2). As a result, they are potentially (and in my experience) considerably faster, but a head-to-head comparison on accuracy with MEDICC2 would be interesting.

Authors’ response: We thank the reviewer for highlighting the need to place our method in the context of existing alternative approaches in the literature. To this end we have included Sitka in our benchmark routine.

Running Sitka on the new simulation data, we found that while it was indeed substantially faster than MEDICC2, it performed worse than all other methods. We double checked that all Sitka runs completed successfully and that the tool did not report any errors. While we cannot rule out that there might be some biases in the simulations that we are not aware of, we want to offer three main reasons to explain the poor performance of Sitka.

1) Sitka is based on a breakpoint method. That means it does not take allele-specific copy-numbers into account nor can it detect events of varying magnitude. Finally, Sitka cannot detect the presence/absence of gains/losses that affect the whole chromosome. However, in our simulations we observe frequent whole chromosome gains and losses. Due to the prevalence of whole chromosome events in real cancer (for example caused by segregation errors during cell division), we believe that this does not unfairly bias our simulations against Sitka but rather constitute a shortcoming of the method.

2) Sitka follows a perfect phylogeny assumption which aims to place breakpoints as internal nodes and assumes that breakpoints are additive along the tree. This assumption is routinely violated in copy-numbers as individual copy-number events can overlap with each other. This is acknowledged in the Sitka manuscript as follows: “we use an observation model which assigns positive probability to arbitrary deviations from the perfect phylogeny assumption, while encouraging configurations where few loci and cells are involved in violations” (Section 2.1). While running Sitka we observed that breakpoint nodes were placed as leaf nodes in roughly ¾ of all cases. While we removed the leaves to make the comparison to our simulations possible, we also note that the fact that Sitka places these nodes as leaves in the first place hints towards an inability to resolve the tree with the perfect phylogeny assumption.

3) Sitka employs a Markov Chain Monte Carlo (MCMC) algorithm to explore the tree space. However, due to the sheer size of the space of possible trees, even for few leaves, we do not believe that this approach is well suited for the task. For some hand-picked examples we created a neighbour joining tree from the breakpoint matrix created by Sitka which resulted in a lower error score for the resulting trees than the ones created with the Sitka MCMC approach. This hints towards a general problem with the MCMC approach. However, a more extensive analysis of the Sitka model is required which would be outside of the scope of this work. In principle a higher value for the number of steps of the MCMC process might result in a higher accuracy. However, here we used the values proposed both by the GitHub repository as well as the Sitka manuscript.

The new simulation results for Sitka have been included in the manuscript as Supplementary Figure 3b and are described in Results section 1.

We would also like to note that we also performed additional investigations into the observed performance differences between MEDICC2 and MEDALT and found that MEDALT cannot deal with homozygous deletions as it calculates the asymmetric pairwise number of evolutionary events between samples. As a segment cannot be gained from copy-number zero, MEDALT assigns a distance of infinity for total copy-number zero and therefore all samples with homozygous deletions are considered to be direct children of the diploid root, resulting in a highly inaccurate tree. Even though we consider this behaviour of MEDALT to be a major shortcoming, we decided to adjust the simulation in order to enable a more fair comparison.

We have included the new Supplementary Figure 3b here for your reference:


5) The SV analysis is interesting but I'm confused as to what "next" or "nearest" copy-number boundary means (nearest to the first MEDICC2 breakpoint, the second breakpoint, or nearest to the SV boundary)? Also, the reported concordance (even for duplications/deletions only) is fairly low (<50%) — while 100% concordance wouldn't be expected, it'd be useful to see this analysis taken further to understand where this divergence is coming from. Currently the reported result is one-sided (how many of MEDICC2's calls show up in the SV data?) but a precision/recall type analysis would yield more insight (how many SV supported duplication/deletions are identified by MEDICC2?). Likewise are the patterns in differences? - e.g., is this jitter (the SV boundary is just out of the 100kb proximity)? Are boundary breakpoints mismatched (that is, each breakpoint overlaps with an SV boundary but not the same event)?

Authors’ response: We thank the reviewer for their comment and apologise that our structural variant analyses were not clearly described in the text. We have added additional explanatory text to the Results and Methods sections and also created Supplementary Figure 6 to better explain our approach:


Supplementary Figure 6: Explanation of the structural variant validation routine.
a) In order to select MEDICC2 events and structural variants for the validation routine we checked for all MEDICC2 events that have at least one overlap with a structural variant. MEDICC2 event A does not overlap with any structural variant and is therefore omitted from the analysis. MEDICC2 event B overlaps with the structural variant B on both breakpoints whereas MEDICC2 event C only overlaps on one side. b) We compared MEDICC2 to a null model that just considers the next segment boundary. In the left example, the next segment boundary does not overlap with the structural variant whereas in the right example it does.


The text changes in the results sections are as follows:

We then counted how often the second SV breakpoint overlaps with i) the second MEDICC2 event boundary, ii) the nearest copy-number segment boundary (with respect to the first breakpoint) or iii) a random copy-number segment boundary on the same chromosome (Methods).

The following clarifications were added to the Methods section:

The “next segment boundary” was defined as the closest segment boundary to the initial overlapping breakpoint in the direction of the other SV boundary. That means if the initial overlap matches the start of a SV, the next boundary will be chosen downstream. Similarly, the random segment boundary was chosen from all possible segment boundaries between the initial overlap and the end of the chromosome in the direction of the second SV boundary.

Revisiting the SV dataset, we first realised that many of the SVs did not overlap with any copy-number changes or only overlapped on one side with a copy-number change. We believe that this effect is due to subclonal SVs as well as general noise in the SV data. As these SVs cannot be detected by MEDICC2 (or any other methods based on the change in copy-numbers), we remove all SVs that do not overlap with a copy-number change on both sides. This selection step already provided an increase in accuracy as shown below (Supplementary Figure 7,21):

PCAWG

Supplementary Figure 7: Model validation using structural variants. Pairs of MEDICC2 events and SVs were chosen based on an overlap of the starting segment. We assume MEDICC2 events to be supported by the SV if the ends also overlap. SVs were filtered based on their type (only duplications and deletions - bottom row - or all types - top row) and on their size.

Gundem et al.

Supplementary Figure 21: Model validation using SVs on multi-sample prostate cancer data. We repeated the SV validation analysis for the 10 patients from the Gundem et al. cohort. Despite the limited number of samples (50 in total) we find a strong overlap of MEDICC2 events with SVs similar to the single-sample data (Supplementary Figure 7).


Precision / Recall
As we select MEDICC2 events and SVs based on an initial overlap (i.e. they have to overlap on a single breakpoint), we do not have “false positives” in the classical sense. However, in order to check how many SVs are captured by our analysis, we created a “reverse analysis”, i.e. check for all SVs whether they overlap with a MEDICC2 event. Here we used the same categories to classify the overlap: one or both breakpoints overlap with a MEDICC2 event.
Note that the number of SVs in the “both” category is the same as the number of MEDICC2 events in that category. The number of MEDICC2 events and SVs with label “one overlap” differs because multiple MEDICC2 events can overlap with a single SV and vice versa. This new reverse analysis is now included in the manuscript as Supplementary Figure 9.


Supplementary Figure 9: Overlap of structural variants with MEDICC2 events. Repeating the SV validation analysis from the perspective of the SVs. To this end, we select all SVs that overlap on at least one breakpoint with a MEDICC2 event (marked as “One segment boundary matches”) and check which percentage of those also overlap on the matching second breakpoint (“Both segment boundaries match”). Note that due to symmetry in the analysis, the number of SVs in the “Both segment boundaries match” category is the same as the number of MEDICC2 events in that category. The number of MEDICC2 events and SVs with label “One segment boundary matches” is not the same because multiple MEDICC2 events can overlap with a single SV and vice versa.


Distance distribution
Next, we investigated the distribution of distances between the MEDICC2 event breakpoint and the expected positions of the SVs. We find that 61% of the missed SV breakpoints were within 10Mbp of the corresponding MEDICC2 event breakpoint (20% within 1Mbp). This figure is now included as Supplementary Figure 8.

Supplementary Figure 8: Model validation using structural variants, distance of mismatched breakpoints. In the SV validation analysis, for every mismatched breakpoint (labelled as “One segment boundary matches” in Supplementary Figure 7) we measured the distance (in Mb) between the mismatched MEDICC2 and SV breakpoints. For all filters, more than 40% of all mismatched MEDICC2 breakpoints were within 10Mb of the correct SV breakpoint.


Finally, in order to look at the effect of mismatched breakpoints (i.e. MEDICC2 overlaps with breakpoints from a different SV), we relaxed the requirement for both breakpoints to stem from the same SV and simply count an overlap if MEDICC2 overlaps with any SV, as requested. As expected this increases the accuracy, however the increase is quite subtle, as visible in the Reviewer figure below.

Reviewer figure:

We believe that these new analyses substantially strengthen our previous findings and confirm that MEDICC2 events are frequently supported by SV calls.

6) For the bulk analysis if A31 - are samples assumed to be homogeneous? How valid is this assumption (with respect to these specific samples) and could violations of this assumption potentially confound results, especially with regards to the conclusion of parallel events?

Authors’ response: We thank the reviewer for this comment and agree that the methods and their inherent assumptions for the bulk analysis of the Gundem et al. data could have been made clearer in the manuscript. We have addressed each point raised by the Reviewer #1’s major comment 6) in parts i), ii), and iii) in the response to reviewer text below. In addition, we have added a section tilted “Bulk data processing and analysis” to the Methods as well as additional text to to the Introduction, main text, and Discussion that are also reproduced at the end of these responses to Reviewer #1’s major comment 6.

i) For the bulk analysis of A31 - are the samples assumed to be homogeneous?

In our analysis, samples were not assumed to be homogeneous but subclonal SCNAs identified as being present in less than 50% of tumour cells within a sample were not taken forward for phylogenetic reconstructions. This is due to the constraints of the subclonal SCNA estimation performed by the Battenberg algorithm that are explained below.

The Battenberg algorithm was designed to analyse individual tumour samples and cannot jointly analyse multiple samples from the same tumour. It models the clonal composition of each genomic segment within each tumour sample independently. When a genomic segment in a sample is identified as being likely to demonstrate subclonal SCNA, it adopts an heuristic to choose from multiple possible SCNA solutions. Such a heuristic is necessary as there can be any number of subclones with distinct subclonal copy-number states to explain the observed state. However, for any given segment, the Battenberg algorithm makes the most parsimonious assumption that there are only two subclones with distinct copy-number states, and that those copy-number states differ by at most by one copy of the chromosomal region.

Next, the likely proportion of tumour cells in a sample that that carry each of the two subclonal SCNA states at this segment (two pairs of a major and a minor copy number at a genomic region) is calculated and the subclonal SCNA that is present in the greatest proportion of cells (≥50%) is reported as the first option in the “nMaj1_A” and “nMin1_A” columns. The other subclonal SCNA inferred as present in the sample in a lower proportion of cancer cells is reported separately. Therefore, while Battenberg detects subclonal SCNAs, it only produces one full genome-wide copy-number profile of the dominant subclone carrying both clonal SCNAs and subclonal SCNAs present in ≥50% of tumour cells. It does not attempt to identify which of the minor subclonal SCNAs present in ≤50% of tumour sample cells co-occur and does not produce full genome wide copy-number profiles of individual “minor” copy-number subclones. Without such genome-wide copy-number profiles of minor subclones we cannot generate input for MEDICC2.

Therefore, for each sample we took the clonal SCNAs and, in regions where a subclonal SCNA was identified, the most prevalent subclonal SCNA state (present in ≥50% of cells) forward for further analysis. As such, the copy-number segmentation data taken forward from all samples to be analysed with MEDICC2 will reflect the copy-number profile of the most prevalent or “dominant” subclone within each sample of the tumour. Our phylogenies are not sample-based SCNA phylogenies and are better described as phylogenies based on the dominant SCNA subclone in each sample.

ii) How valid is this assumption (with respect to these specific samples) ?

While we do not assume that these samples are homogeneous, we do however, as described above, take only the genome-wide copy-number profile of the dominant subclone from each sample forward for further analysis. In the case of A31, all individual samples from this tumour when analysed using the Battenberg algorithm have been shown to demonstrate subclonal SCNAs. These subclonal SCNAs will not be taken forward in our analyses due to the lack of genome-wide copy-number profiles specific to minor subclones and as such our phylogenies are unable to reflect all the SCNA information inferred by Battenberg.

Gundem et al. classified the SNV-based clone phylogenies from the 10 prostate cancer cases analysed as demonstrating either polyclonal seeding of metastatic samples (observed in five tumours - A22, A24, A31, A32, and A34) or monoclonal seeding of metastatic samples (observed in the other five tumours - A10, A12, A17, A21, and A29). Polyclonal seeding describes the situation when multiple genetically distinct subclones colonise a single metastatic site resulting in a complex, mixed, subclonal architecture whereas monoclonal seeding describes the situation where each metastatic site was colonised by only a single subclone. Therefore, as A31 is classed as demonstrating polyclonal seeding, it is clear that there are minor subclonal populations present in tumour samples that are not captured by our use of the dominant subclone per sample as defined by Battenberg output. Nonetheless, as the reviewer notes, in the case of A31 the topologies of the SNV-based clone phylogeny and MEDICC2 phylogeny based on the dominant subclone for each sample match. However, this is not true for all the tumours and we discuss this in the main text:

“We observed concordance between the MEDICC2 phylogenies and the SNV-based clone phylogenies reproduced from Gundem et al. in 6 out of 10 tumours and partial concordance in the remaining 4 tumours.”

Finally, if genome-wide copy-number profiles specific to the minor subclones (those present in <50% of tumour cells in a bulk sample) were produced by an algorithm designed for bulk sequencing then these could be used as input for MEDICC2 to produce phylogenetic reconstructions. We have attempted to make this point clear in the Discussion section of the manuscript:

“Since MEDICC2 does not itself infer SCNAs from either bulk or single cell raw data and is agnostic of the sequencing modality used to generate its input, its results are dependent upon accurate cell, bulk sample, or subclone-level copy number profiles, and the resolution of the original data.”

iii) ... could violations of this assumption potentially confound results?

As described in sections i) and ii) in our analysis we do not assume the samples to be homogeneous but only the dominant subclone from each sample is taken forward for multi-sample SCNA analysis with Refphase and MEDICC2 phylogeny construction. There is therefore additional SCNA heterogeneity detected by the Battenberg algorithm that was not included in our phylogenetic reconstruction of this particular tumour’s evolution. In addition, there may be subclonal SCNAs at cellular prevalence in the tumour below the limits of detection by the Battenberg algorithm. However, these omissions of subclonal SCNAs will not in themselves confound results - MEDICC2’s phylogenies will be consistent with the dominant subclone per sample input provided.

If an SCNA estimation algorithm other than Battenberg could produce full genome-wide copy-number profiles for both the major and minor SCNA subclones present in each tumour sample then MEDICC2 would be able to incorporate both major and minor SCNA subclones into its inferred phylogeny.

iv) .. especially with regards to the conclusion of parallel events?

The major or “dominant” SCNA subclone genome-wide copy-number profiles inferred by Battenberg algorithm for each tumour sample are used as input to Refphase for analysis at the tumour level. Refphase performs haplotype-specific analysis across multiple samples from a single tumour by utilising the frequencies of same heterozygous SNPs in the different tumour samples (Watkins et al., 2020).

This may reveal instances of mirrored subclonal allelic imbalance (MSAI) (Jamal-Hanjani, et al. 2017; Watkins et al. 2020) in which SCNAs that affect opposite haplotypes in different samples from the same tumour result in different haplotypes having a higher copy-number in different samples. This causes the identity of the heterozygous SNPs belonging to the most prevalent haplotypes to differ between tumour samples.

A subset of these MSAI events may be considered parallel events. These parallel events involve the same class of SCNA, for example a gain on the “A” haplotype in one sample (“AAB”) and an independent gain on the “B” haplotype in another sample (“ABB”) may constitute evidence for convergent evolution and positive selection. In contrast, independent evolution involving differing classes of SCNA (e.g. a gain of “A” in one sample resulting in an “AAB” copy number state and a loss event in another sample resulting in a lone copy of the “B” allele in another sample) in different samples from the same tumour may result in MSAI but not constitute parallel evolution of the same class of SCNA.

In bulk sequencing these parallel events are identified by identifying the differing sets of heterozygous SNPs with the highest frequencies within samples from the same tumour. The identity of these high-frequency SNPs will be determined by the dominant, most prevalent, copy-number subclone and therefore are not determined by minor subclonal SCNAs (present in < 50% of tumour cells) being excluded from MEDICC2’s phylogenetic analyses. Therefore, the selection of the genome-wide copy-number profiles generated by the Battenberg algorithm that correspond to the dominant copy-number subclone within each tumour sample will not create spurious parallel events.

To reflect these thoughts in the manuscript, we have added a section “Bulk data processing and analysis” to the Methods section in the manuscript that covers these details. We further touch on various aspects of this in the discussion section, for example by stating more explicitly that MEDICC2 itself does not infer SCNAs but happily works with subclone-level copy-numbers if provided.

For the convenience of the reviewer the “Bulk data processing and analysis” to the Methods section is reproduced below.

“Bulk data processing and analysis
The bulk SCNA analysis was performed in two stages. First, as part of the PCAWG cohort [25], each tumour sample was analysed individually with the Battenberg algorithm [73] to produce a sample-level inferred purity, ploidy, and copy-number segmentation with associated allele-specific copy-number states. Only copy-number segmentation from autosomes was included in the study. The Battenberg algorithm is able to detect subclonal SCNAs. However, in our analyses we used only a single full genome-wide copy-number profile representing the dominant subclone per tumour sample comprising both clonal SCNAs and those subclonal SCNAs present in ≥50% of tumour cells within that sample.

Next, these Battenberg outputs, as well as the input data used for Battenberg including heterozygous SNP B-allele frequencies, for all samples from a tumour were jointly analysed to produce haplotype-specific SCNAs through the application of Refphase, a multi-sample reference phasing algorithm [3]. We then defined a tumour consensus segmentation profile by combining breakpoints from each SCNA segmentation profile from each individual tumour sample. This tumour-level analysis of haplotype-specific SCNAs may reveal instances of mirrored subclonal allelic imbalance (MSAI) [3,4] in which SCNAs that affect the opposite haplotypes in different samples from the same tumour result in different haplotypes having a higher copy-number in different samples. This causes the identity of the heterozygous SNPs belonging to the most prevalent haplotypes to differ between tumour samples.

A subset of these MSAI events may be considered parallel events. These parallel events involve the same class of SCNA, for example a gain on the “A” haplotype in one sample (e.g. from “AB” to “AAB”) and an independent gain on the “B” haplotype in another sample (e.g. from “AB” to “ABB”) may constitute evidence for convergent evolution and positive selection. In contrast, independent evolution involving differing classes of SCNA in different samples from the same tumour may result in MSAI but not constitute parallel evolution of the same class of SCNA. An example of this could include a gain of “A” in one sample resulting in an “AAB” copy-number state and a loss event in another sample resulting in a lone copy of the “B” allele.

Reference phasing, as described above, considers all genomic segments independently of each other, and while often segments span entire chromosomes, sometimes different reference samples may be chosen for different segments on the same chromosome. Since phasing is restricted to continuous regions of allelic imbalance, we estimate phasing along the genome across multiple segments within chromosomes (“horizontal phasing”) by using an evolutionary criterion. Briefly, the assignment of heterozygous SNPs to “A” and “B” haplotypes for all bins within a single chromosome is chosen to minimise the number of copy-number events between a diploid normal sample and the tumour.”

Minor Comments

1) The model section in methods would greatly benefit from a figure (even supplementary).

Authors’ response: We thank the reviewer for this important comment. We have updated main Figure 1b to provide a better running example throughout the manuscript. We have also added a new Supplementary Figure 1 which depicts an optimal and suboptimal solution for the MED problem using that running example, shows simplifications of the FSTs and FSAs used in MEDICC2 and illustrates the composition strategy. References to this new Supplementary Figure 1 have been added to the "Model" subsection of the "Methods" section.

2) "Validation dataset" and "Overlap of MEDICC2 events with structural variants" mentions the data of "individual cells" when discussing PCAWG bulk tumor samples - is this an error?

Authors’ response: We thank the reviewer for identifying this error in which “individual cells” should have been written as “individual samples”. We have corrected the text.

3) P.20, line 8: "poisson" -> "Poisson"

Authors’ response: This has been corrected.

4) P.20, line 19: "The start of an event of gain or loss events in the sequence The start of an event"

Authors’ response: This has been corrected.

5) P.27, line 21: "The logged ratios Logged ratios"

Authors’ response: These have been changed to “Log Ratios”

6) The paper switches back and forth between "tumor" and "tumour"

Authors’ response: The main text, all Figures, and all Figure legends have been checked for “tumor” and changed to “tumour”.

Reviewer #2
The authors present MEDICC2, a significant update to their previous similarly named method. The new method is able to account for whole genome duplication, uses a new lazy method for efficient computation of the FST score, and is able to reconstruct ancestral allele specific copy number states. The authors show broad utility by applying their data to the problem of WGD detection in single sample WGS, and phylogenetic reconstruction in multi-sample WGS and single cell low pass WGS. The software implementing the method is high quality and easy to use. I list my major and minor concerns below.
Major comments

1. Page 4 line 29, the authors should emphasize that the calculation of MED is linear in the length of the sequence. It would be helpful to have a more thorough breakdown of the time complexity relative to number of cells/samples and max copy number.

Authors’ response: We would like to thank the reviewer for pointing out the missing information regarding the runtime of MEDICC2. For the datasets used in the manuscript (Gundem et al. 2015 and Minussi et al. 2021) the runtime is reported as Supplementary Tables 2 and 3. In the case of the single-cell dataset (Minussi et al. 2021) we have furthermore shown the effect of using multiple cores. In order to get a more detailed view on the runtime of MEDICC2 we have created extensive runtime analysis through simulations, the results of which are reported at the end of Results Section 1 (“Inferring phylogenies…”), Supplementary Table 1 and Supplementary Figure 5.

We also wanted to clarify that while the calculation of the MED is linear in the number of segments, the overall runtime of MEDICC2 is not. As the calculation of a single MED is linear in the number of segments and the overall runtime scale with N^2 (N being the number of samples), the overall runtime also scales with the number of segments. In order to clarify this point we added the following line to the main text:

While the MED computation between two samples is linear in the number of genomic segments, pairwise MED calculation between all samples makes the overall algorithmic complexity quadratic in the number of samples.

For the simulation approach we used the same framework that was also used for the reconstruction accuracy benchmarking and simulated a wide range of trees with between 3 and 1000 samples and between 100 and 400 segments. Generally speaking, running MEDICC2 on data with ≤25 samples is feasible in a few minutes on a personal computer without extensive computational resources. However, in the case of large-scale single-cell datasets, as the calculation of evolutionary distances scales with O(N^2), we recommend the use of MEDICC2’s parallelization capabilities by running it with multiple cores.


Supplementary Figure 5: Performance on simulated data.
The overall runtime of MEDICC2 is quadratic in the number of samples. MEDALT’s total runtime is also quadratic in the number of samples but is less strongly affected by the number of segments since it is not computing the symmetric edit distance via a common ancestor. Sitka has a linear runtime relationship to the number of samples. The data was simulated using our simulation framework with a mutation rate of 0.05 and 100, 200 and 400 segments with 5 independent simulations per datapoint. Inferences that took longer than 24 hours were omitted and error bars represent standard errors between the 5 runs. Some data points do not have an error bar because 4 out of the 5 runs took longer than 24 hours, for example MEDICC2 (single core) 750 samples with 100 segments.


With respect to the copy-numbers, the limit of maximum copy-number 8 is currently hard coded and the finite state transducer is pre-built with copy-numbers ranging from 0 to 8. A lower copy-number therefore does not result in a smaller finite state transducer and therefore is not linked to a speed increase. In future iterations, we plan to make multiple pre-compiled FSTs available to the users such that MEDICC2 automatically selects the smallest, appropriate FST from which datasets with low copy-number would benefit.


2. In figure 5, why is chromosome 5 minor allele copy number heterogeneous in the clones at the bottom half of the phylogeny. The q arm of chromosome 5 is inferred to be lost producing LOH many times throughout the phylogeny. Is there a solution for which this region is lost once? Would some other region then be inferred to be recurrently gained or lost for such a solution? How are the authors sure of the presented solution over a solution for which 5q is lost only once? Alternatively, are some of the calls of loss or lack of loss related to the noisiness of the data?

Authors’ response: We thank the reviewer for raising the subject of the effect of noise in the input data on MEDICC2’s output. MEDICC2 creates its phylogenetic tree using the neighbour joining algorithm from the pairwise distance matrix between the samples. Neighbour joining provides an approximation to the NP-complete Steiner tree problem in phylogenetics (see also new Discussion) which, paired with our minimum event distance, aims to minimise the events observed in the tree. In the case of conflicting signal in the data, parallel events will be introduced to account for these conflicts. These parallel events can be due to noise or actual parallel evolution. Single-cell data is naturally quite noisy and in the case of the 5q loss, choosing a tree in which 5q is recurrently lost therefore can be considered as the “lesser evil” with respect to an alternative solution in which other segments would be recurrently lost or gained (in particular chromosomes 1, 7, 10 and 11).

In order to emphasise this point we have run MEDICC2 on two subsets of the dataset, one which has copy-number 1|1 on 5q and the other with copy-number 2|0 (see Reviewer figure below). On the lower half of the 2|0 part, one can clearly see a group of cells that would be more appropriately grouped together with the 1|1 cells. This is visible looking at chromosomes 1, 7, 10 and 11. These cells are exactly the ones that MEDICC2 places together with the 1|1 cells, and which seem to recurrently lose 5q in the original MEDICC2 tree.

We acknowledge that this was not sufficiently emphasised in the previous version of the manuscript and we discuss MEDICC2’s dependence on accurate copy-number profiles in the Discussion section.

Reviewer Figure:


3. The authors limited comparison with MEDALT, a minimum spanning tree based method, could be improved. The authors do not specify how they convert the spanning tree to a phylogeny for comparison with MEDICC2 results. A reasonable approach would be to take all internal node samples/cells and place them on leaves connected to the internal node by a zero length branch. Is something like this what was done? Can the authors provide more insight into the differences between the MEDICC2 and MEDALT solutions and why MEDALT performs so poorly?

Authors’ response: We apologise for not describing the approach we took with regards to converting the spanning trees to phylogenies in the previous version of the manuscript. We now have added a new subsection into Methods termed “Comparison to other methods” to describe the way MEDALT spanning trees were converted into phylogenies. The reviewer was correct in that internal node samples were replaced with internal tree nodes and the original sample connected with branch length zero. We have updated the corresponding section in the Methods:

After running MEDALT, we transformed the minimum spanning trees into phylogenies. To this end, we replaced all cells that are positioned on internal nodes of the tree with dummy nodes and added the samples back in with branch length zero as children leaves of the respective dummy nodes. Note, that the minimum spanning trees can create multifurcations which we cannot resolve and are left as multifurcating trees in the phylogeny.

We performed additional investigations into the observed performance differences between MEDICC2 and MEDALT and found that MEDALT cannot deal with homozygous deletions as it only calculates the asymmetric MED between samples. As a segment cannot be gained from copy-number zero, MEDALT assigns a distance of infinity between any sample that has copy number zero and any other sample that does not have copy number zero at that locus. Additionally, any pair of samples with one copy number zero segment each in different loci will always have a distance of infinity from “both sides”. In any such case MEDALT considers all samples with homozygous deletions to be direct children of the diploid root, resulting in a highly inaccurate tree.

Even though we consider the behaviour of MEDALT to be a serious shortcoming, we decided to adjust the simulation in order to enable a more fair comparison. To this end, we adjusted the simulation to never delete segments from haplotype 2, thereby avoiding homozygous deletions. The update to the simulation is mentioned as follows in the Methods section:

In order to prevent the occurrence of homozygous deletions, we prevented deletions of haplotype 2 which in turn also lowered the effective loss to gain ratio.

The change to the simulation resulted in a decline of reconstruction error of MEDALT while leaving the other methods mainly unchanged. MEDALT still performs worse than MEDICC2; however, now the difference is less striking. We have therefore updated Figure 2b as follows:


In our understanding, there are two main reasons for the (still) low reconstruction performance of MEDALT.

1) Similar to MEDICC2, MEDALT creates a pairwise matrix of minimum event distances between all pairs of samples. However, MEDALT cannot deal with allele-specific copy-numbers, therebye discarding important information. Secondly, MEDALT does not model WGDs which results in a skewed distance distribution in the presence of WGDs. Finally, as pointed out above, MEDICC2 calculates a symmetric event distance (that means, the event between two samples is calculated via the most recent common ancestor), whereas MEDALT calculates the distance asymmetrically between any two samples. This assumes a parent-child relationship between all pairs of samples which, as we describe below, is not accurate for tumour samples. The asymmetric distance calculation also explains why MEDALT cannot deal with homozygous deletions.

2) MEDALT only solves the minimum spanning tree problem (MST), not the actual Steiner tree phylogeny problem (see also new Discussion). While the MST can be efficiently solved in polynomial time (likely the reason for its use in MEDALT), MSTs are not phylogenies. The MST formulation implicitly assumes that all ancestral nodes are directly observed in the sequencing data, a largely invalid assumption since the internal nodes represent a previous stage of the cancer evolution that in most cases has been surpassed by the current cancer subclones. The internal nodes are therefore generally not accessible by experiments.

In our simulation the internal nodes are extinct evolutionary states that are inferred. We would argue that this is a realistic reflection of how data is present in real-life data and therefore the MST is not a suitable tool for phylogenetic reconstruction.

MSTs can always be transformed into phylogenies (as described above). Some phylogenies therefore can be represented correctly by a MST. However, this property is only true for ladder-like trees. In balanced trees on the other hand, MSTs cannot be restructured to represent the underlying phylogeny. See the Reviewer figure below for the simplest example with five samples. MEDALT places sample “a” as the MRCA. Transforming the MST into a phylogeny results in a trifurcation after the MRCA. The correct evolutionary relationship (i.e. the fact that the samples b, c, d and e are more closely related to each other than to sample a) is lost. Note that for simplicity of display we changed all branch lengths to be 1.

Reviewer figure:


We have rewritten the discussion section of the manuscript to better explain the simplifications used by MEDALT and other competing tools and hope that this clarifies the point raised by the reviewer.

4. The WGD results on page 8 are somewhat confusing. What is the breakdown of PCAWG WGD annotations for the 2778 cases? As written it sounds as if all 2778 are WGD.

Authors’ response: We would like to thank the reviewer for this helpful comment. We acknowledge that our description of our WGD inference results from the PCAWG samples was unclear and have adapted the passage as follows:

“Next, we tested MEDICC2’s ability to detect WGDs by comparing our results to the WGD status in PCAWG, which was inferred from the relationship between tumour ploidy and the percentage of the genome affected by LOH across the cohort25 (Methods) with 818 samples labelled as WGD positive and 1960 as WGD negative.
MEDICC2 correctly predicted the WGD status of 2668 out of the 2778 cases (96.0%), 12 of which were predicted to have undergone two consecutive WGD events. All of the 110 incorrect predictions were false negatives, i.e. they were labelled as WGD in PCAWG but not called by MEDICC2.”

5. Page 8, line 20, how many of the 110 were annotated as WGD uncertain?

Authors’ response: We thank the reviewer for pointing out this missing information in the previous version of the manuscript. We have changed the text as stated below. Note also that false predictions that are labelled “WGD uncertain” are marked as grey circles in Figure 2D.

“Since PCAWG WGD annotations are also based on biological data with inherent noise and may contain errors, we investigated whether the 110 missed cases of WGD were marked as ‘WGD uncertain’ by the PCAWG heterogeneity and evolution working group. Indeed, tumours with status ‘WGD uncertain’ were significantly overrepresented amongst these tumours (17 out of the 110 incorrect MEDDIC2 predictions, P =1.210-8, chi-square test). To increase sensitivity and in order to mitigate the effect of noisy data, we created 100 bootstrap replicates for each sample (Methods) and calculated the WGD evidence scores for each replicate. Marking samples as WGD if at least 5% of their bootstrap runs exhibited at least one WGD event increased the detection accuracy of WGDs to 98.8% (33 incorrect predictions: 6 false positives and 27 false negatives) (Figure 2d, Supplementary Figure 4) while maintaining an over-representation of false predictions among tumours with status ‘WGD uncertain’ (7 out of the 33 incorrect MEDDIC2 predictions, P=9.610-6, chi-square test). Bootstrap sampling identified an additional 15 samples (total of 27) that underwent two successive WGDs (Figure 2d).”


6. Page 8, line 27, 5% of bootstrapped samples is a very low bar for calling WGD. What threshold is actually needed to provide a significant boost in detection accuracy?

Authors’ response: We thank the referee for highlighting this point and agree that this threshold was not well justified in the previous version of the manuscript.

We have therefore created a new Supplementary Figure 11 exploring the effect of the threshold for the WGD bootstrap detection. Any threshold value below 54% will increase the accuracy of the WGD detection with respect to the standard WGD detection. For the precision, recall and f1 score for all threshold values please see Supplementary Figure 11a. As expected, the recall falls with increasing threshold while the precision rises (reaching a value of 1.0 for a threshold of 20%). The accuracy and f1 score both reach their maximum value (0.990 and 0.983 respectively) for a threshold of 3%. Both values decrease after this peak, however, it should be noted that both accuracy and f1 score stay reasonably high for threshold values ≤10%. This relative independence of the classifier performance from the choice of threshold value can also be seen from the precision-recall curve (Supplementary Figure 11) and its AUC of 0.99. Since we did not want to optimise threshold selection for the cohort at hand, but provide a general approach for WGD detection, we settled on the ad-hoc threshold value of 5%.

In addition to the new Supplementary Figure 11 we have made the following addition to the text:

We found the WGD detection accuracy to be largely independent of the bootstrap percentage threshold chosen (area under the precision-recall curve AUC=0.99, Supplementary Figure 11).


Supplementary Figure 11: Effect of the bootstrap percentage threshold for the bootstrap WGD detection.
a) Accuracy, precision, recall and f1 score for all possible values of the bootstrap percentage threshold for WGD detection. As expected, the recall decreases with increasing threshold while the precision rises (reaching a value of 1.0 for a threshold of 20%). The accuracy and f1 score both reach their maximum value (0.990 and 0.983 respectively) for a threshold of 3%. Both values decrease after this peak. However, both accuracy and f1 score stay reasonably high for threshold values <=10%. b) Precision-recall curve for the MEDICC2 bootstrap WGD detection with an area under the curve of 0.99.


7. The authors apply MEDICC2 to the Gundem et al., for which subclonality at sampled sites like makes sample level phylogenies less informative for certain patients. For A31 it appears that a sample phylogeny is equivalent to the clone phylogeny. Can the same be said for the data from the other patients? How much of an issue is the subclones and their presence / mixing across multiple sites?

Authors’ response: We thank the reviewer for this comment and agree that the methods and their assumptions for the bulk analysis of the Gundem et al. data could have been made clearer. Furthermore, the reviewer’s question regarding the presence of subclones and their mixing across multiple samples from a tumour is an excellent one and has led us to make alterations throughout the manuscript.

We have addressed each point raised by the Reviewer #2’s major comment 7) in the text below as well as adding sections titled “Bulk data processing and analysis” and “Bulk phylogeny comparison” to the Methods (see at the bottom of this response). Finally, we have added text to to the Introduction, main text, and Discussion that are reproduced at the end of these responses to Reviewer #2’s major comment 7.

“Bulk phylogeny comparison
The SNV-based phylogenetic reconstructions were reproduced from Gundem et al. “Figure 2: Subclonal structure within 10 metastatic lethal prostate cancers.” with additional information from “Supplementary Table - Subclones” (also from Gundem et al.) using the following columns: “cluster” that details the clusters used to generate the original phylogenies, “cluster.colour” that is the cluster’s colour in the original publication, “# subs from WGS data” that shows the number of SNVs present within each cluster, “samples containing” that shows which samples contain each cluster, and “CCF values” that show the cancer cell fraction values for each cluster in each sample that it is present in. The branch lengths of these phylogenies are determined by the number of SNVs present in each cluster that contributes to the branch.

We compared unnormalised root-to-leaf lengths between the SNV-based clone phylogenies and the MEDICC2 phylogenies at the cohort level, and when discussing individual tumours, we normalised each root-to-leaf length by the maximum observed root-to-leaf length of the corresponding phylogeny for that tumour. To compare topologies, we used the regular Robinson-Foulds distance (as implemented in the R package ape 5.6.1) to evaluate similarity between the SNV-based clone phylogenies and the MEDICC2 trees. We took the leaves as annotated in “Figure 2: Subclonal structure within 10 metastatic lethal prostate cancers.” from Gundem et al. When multiple samples were listed as a single leaf we introduced a bifurcation at this position e.g. tumour A12 with leaf AC. In addition, when multiple samples were listed in multiple leaves we chose the leaf with each sample’s largest CCF contribution to represent that sample. Finally, the Gundem et al. trees contain multifurcations, which support a variety of possible bifurcating trees. For comparison with a binary hypothesis tree as inferred by MEDICC2, we considered all possible bifurcations obtainable from any given multifurcation, and used the minimum Robinson-Foulds distance across this set of bifurcations to determine a possible match.”


i) For A31 it appears that a sample phylogeny is equivalent to the clone phylogeny. Can the same be said for the data from the other patients?

In response to the reviewer’s comment we have carefully compared the SNV-based clone phylogenies and the MEDICC2 phylogenies for all the bulk prostate cancer cases once more. This has enabled us to correct an error present in the original manuscript as well as highlight the difference in resolution provided by the SNV-based clone phylogenies that are able to capture multiple subclonal populations within a single tumour sample and our MEDICC2 phylogenies that are based on the dominant (most prevalent) subclone per tumour sample as identified by the Battenberg algorithm. Please see our response to Reviewer 1’s major point 6 for further detail regarding the difference in resolution between the SCNA data used as input to MEDICC2 and the SNV-based cancer cell fraction based phylogenies from Gundem et al.


In the previous version of the manuscript we stated the following:

“We observed an overall agreement between the MEDICC2 and SNV trees, with identical topologies for 7 out of 10 patients.”

Which now reads:

“we observed exact concordance, defined as Robinson-Foulds distances of zero, between the SCNA-based MEDICC2 phylogenies and the SNV-based clone phylogenies reproduced from Gundem et al. in 6 out of 10 tumours (A10, A12, A21, A29, A31, and A34) and partial concordance in the remaining 4 tumours with Robinson-Foulds distances greater than zero (A17:3, A22:7, A24:2, and A32:2).”


ii) How much of an issue is the subclones and their presence / mixing across multiple sites?

Gundem et al. classified the resulting SNV-based clone phylogenies as demonstrating either polyclonal seeding of metastatic samples (observed in five tumours - A22, A24, A31, A32, and A34) or monoclonal seeding of metastatic samples (observed in the other five tumours - A10, A12, A17, A21, and A29). Polyclonal seeding describes the situation when multiple genetically distinct subclones colonise a single metastatic site resulting in a complex, mixed, subclonal architecture whereas monoclonal seeding describes the situation where each metastatic site was colonised by only a single subclone.

The four cases in which the topologies of the SNV-based cluster phylogenies for which we observe only partial concordance with the MEDICC2 phylogenies were A17, A22, A24, and A32. Interestingly, 3 of these 4 cases (A22, A24, and A32) were classed as demonstrating polyclonal seeding. Therefore, 4/5 of the cases classed as demonstrating monoclonal seeding and 2/5 of the cases demonstrating polyclonal seeding show concordance between the SNV-based clone and MEDICC2 dominant subclone phylogenies. In addition, tumour A17 was classed as demonstrating monoclonal seeding but the authors from Gundem et al. state “we consider the evidence for polyclonal seeding in this patient to be inconclusive. For this reason, we have taken a conservative approach and classified A17 as monoclonal...”.

The construction of a phylogeny from the copy-number profile of the dominant (most prevalent and in ≥50% of tumour cells) subclone per tumour sample compared to SNV clustering that may reveal minor subclones (potentially present in <50% of tumour cells) will offer different different levels resolution. Given that all the cases that with partial phylogeny concordance either demonstrate or show some evidence of polyclonal seeding, this may suggest that it may often be the input to MEDICC2 that results in these phylogeny differences rather than a fundamental difference in SCNA dynamics compared to SNV dynamics or systematic error in the phylogeny construction.

In order to make this potential relationship between polyclonal seeding of the metastatic samples and partial rather than full concordance between the MEDICC2 and original SNV-based clone phylogenetic reconstructions we have added the following to the main text:

“We observed exact concordance, defined as Robinson-Foulds distances of zero, between the SCNA-based MEDICC2 phylogenies and the SNV-based clone phylogenies reproduced from Gundem et al. in 6 out of 10 tumours (A10, A12, A21, A29, A31, and A34) and partial concordance in the remaining 4 tumours with Robinson-Foulds distances greater than zero (A17:3, A22:7, A24:2, and A32:2). Notably, using SNV cancer cell fraction information metastatic samples from A22, A24, and A32 were classified as demonstrating polyclonal seeding with multiple subclones present within individual samples while A17 was classified as showing inconclusive evidence of such polyclonal seeding (Gundem et al. 2015). The likely CNPs of these multiple subclones within a single sample will not be captured by taking the CNP of the dominant subclone within that sample and therefore this may contribute to the partial concordance observed between the MEDICC2 and SNV-based phylogenies in these tumours.”

Finally, in order to clarify that MEDICC2 does not itself infer copy number profiles we have added the following sentence to the discussion:

“Since MEDICC2 does not itself infer SCNAs from either bulk or single cell raw data and is agnostic of the sequencing modality used to generate its input, its results are dependent upon accurate cell, bulk sample, or subclone-level copy number profiles, and the resolution of the original data.”


The two added methods sections relevant to this point: “Bulk data processing and analysis” and “Bulk phylogeny comparison” have been reproduced here for the convenience of the reviewer.

“Bulk data processing and analysis
The bulk SCNA analysis was performed in two stages. First, as part of the PCAWG cohort [25], each tumour sample was analysed individually with the Battenberg algorithm [73] to produce a sample-level inferred purity, ploidy, and copy-number segmentation with associated allele-specific copy-number states. Only copy-number segmentation from autosomes was included in the study. The Battenberg algorithm is able to detect subclonal SCNAs. However, in our analyses we used only a single full genome-wide copy-number profile representing the dominant subclone per tumour sample comprising both clonal SCNAs and those subclonal SCNAs present in ≥50% of tumour cells within that sample.

Next, these Battenberg outputs, as well as the input data used for Battenberg including heterozygous SNP B-allele frequencies, for all samples from a tumour were jointly analysed to produce haplotype-specific SCNAs through the application of Refphase, a multi-sample reference phasing algorithm [3]. We then defined a tumour consensus segmentation profile by combining breakpoints from each SCNA segmentation profile from each individual tumour sample. This tumour-level analysis of haplotype-specific SCNAs may reveal instances of mirrored subclonal allelic imbalance (MSAI) [3,4] in which SCNAs that affect the opposite haplotypes in different samples from the same tumour result in different haplotypes having a higher copy-number in different samples. This causes the identity of the heterozygous SNPs belonging to the most prevalent haplotypes to differ between tumour samples.

A subset of these MSAI events may be considered parallel events. These parallel events involve the same class of SCNA, for example a gain on the “A” haplotype in one sample (e.g. from “AB” to “AAB”) and an independent gain on the “B” haplotype in another sample (e.g. from “AB” to “ABB”) may constitute evidence for convergent evolution and positive selection. In contrast, independent evolution involving differing classes of SCNA in different samples from the same tumour may result in MSAI but not constitute parallel evolution of the same class of SCNA. An example of this could include a gain of “A” in one sample resulting in an “AAB” copy-number state and a loss event in another sample resulting in a lone copy of the “B” allele.

Reference phasing, as described above, considers all genomic segments independently of each other, and while often segments span entire chromosomes, sometimes different reference samples may be chosen for different segments on the same chromosome. Since phasing is restricted to continuous regions of allelic imbalance, we estimate phasing along the genome across multiple segments within chromosomes (“horizontal phasing”) by using an evolutionary criterion. Briefly, the assignment of heterozygous SNPs to “A” and “B” haplotypes for all bins within a single chromosome is chosen to minimise the number of copy-number events between a diploid normal sample and the tumour.”


“Bulk phylogeny comparison
The SNV-based phylogenetic reconstructions were reproduced from Gundem et al. “Figure 2: Subclonal structure within 10 metastatic lethal prostate cancers.” with additional information from “Supplementary Table - Subclones” (also from Gundem et al.) using the following columns: “cluster” that details the clusters used to generate the original phylogenies, “cluster.colour” that is the cluster’s colour in the original publication, “# subs from WGS data” that shows the number of SNVs present within each cluster, “samples containing” that shows which samples contain each cluster, and “CCF values” that show the cancer cell fraction values for each cluster in each sample that it is present in. The branch lengths of these phylogenies are determined by the number of SNVs present in each cluster that contributes to the branch.

We compared unnormalised root-to-leaf lengths between the SNV-based clone phylogenies and the MEDICC2 phylogenies at the cohort level, and when discussing individual tumours, we normalised each root-to-leaf length by the maximum observed root-to-leaf length of the corresponding phylogeny for that tumour. To compare topologies, we used the regular Robinson-Foulds distance (as implemented in the R package ape 5.6.1) to evaluate similarity between the SNV-based clone phylogenies and the MEDICC2 trees. We took the leaves as annotated in “Figure 2: Subclonal structure within 10 metastatic lethal prostate cancers.” from Gundem et al. When multiple samples were listed as a single leaf we introduced a bifurcation at this position e.g. tumour A12 with leaf AC. In addition, when multiple samples were listed in multiple leaves we chose the leaf with each sample’s largest CCF contribution to represent that sample. Finally, the Gundem et al. trees contain multifurcations, which support a variety of possible bifurcating trees. For comparison with a binary hypothesis tree as inferred by MEDICC2, we considered all possible bifurcations obtainable from any given multifurcation, and used the minimum Robinson-Foulds distance across this set of bifurcations to determine a possible match.”


8. Page 19, line 14, the authors normalize by the maximum root to leaf distance. Was this done independently per left (sample) or was the entire tree normalized by the longest branch in the tree? The SNV / SCNA branches appear to be locally correlated (dependent on normalization) with the exception of the trunk, the branch to E (burst of complex SCNA) and the branch to C (driver SNVs?). The text here could be a little more clear and care should be taken with describing the normalization. Possibly an independent normalization (based on the cohort?) would better expose the branches with expected ratio of SNV to SCNA vs those that have unexpected differences in rates?

Authors’ response: We agree with the reviewer that the methods regarding the comparisons between the SNV-based clone phylogenies and MEDICC2 phylogenies could have been made clearer. In order to address this, we have added a Methods section “Bulk phylogeny comparison” describing both the normalisation and the comparison between the Gundem et al. SNV-based clone phylogenies and the MEDICC2 SCNA-based phylogenies.


With respect to the normalisation, we carefully evaluated multiple different ways of normalising the branch lengths to make them comparable. In the end, we don’t believe that a simple normalisation based on for example the maximum root-to-leaf length across the whole cohort would improve transparency of the comparisons or help with the analysis. While more sophisticated normalisation methods might be conceivable, we believe this would be beyond the scope of this paragraph, whose purpose it is mainly to illustrate the overall agreement between SNV and SCNA trees and highlight some important differences. Therefore we decided to always report the actual branch lengths in number of events for maximum interpretability and transparency, and additionally note the maximum root-to-leaf distance per tree to put the actual branch length in context. We hope that this satisfies the concerns of the reviewer. We also understand that the reviewer would like to see some cohort-level comparisons on the branch lengths, which we now added in form of an overall correlation analysis (new Supplementary Figure 22), which is reproduced below.


Supplementary Figure 22: Comparison of root-to-leaf distances across the cohort
Scatterplot showing the absolute root-to-leaf lengths for samples from the MEDICC2 phylogenies and SNV-based clone phylogenies reproduced from Gundem et al. Data points are coloured by the tumour the samples are from and their shape corresponds to whether the MEDICC2 and SNV-based clone phylogenies demonstrated concordance (circles) or partial concordance (triangles) as defined by the Robinson-Foulds distance. The grey shaded area represents the 95% confidence interval. ρ and P values are from a Spearman correlation test.

We use this new analysis to identify that there is a significant correlation between SNV root-to-leaf distances and the MEDICC2 SCNA-based root-to-leaf distances in our Gundem et al. cohort. However, we observe two tumours (A34 and A17) as particular outliers with long mutational root-to-leaf distances.

Please find the new methods section added to help address the reviewer’s comment below:

“Bulk phylogeny comparison
The SNV-based phylogenetic reconstructions were reproduced from Gundem et al. “Figure 2: Subclonal structure within 10 metastatic lethal prostate cancers.” with additional information from “Supplementary Table - Subclones” (also from Gundem et al.) using the following columns: “cluster” that details the clusters used to generate the original phylogenies, “cluster.colour” that is the cluster’s colour in the original publication, “# subs from WGS data” that shows the number of SNVs present within each cluster, “samples containing” that shows which samples contain each cluster, and “CCF values” that show the cancer cell fraction values for each cluster in each sample that it is present in. The branch lengths of these phylogenies are determined by the number of SNVs present in each cluster that contributes to the branch.

We compared unnormalised root-to-leaf lengths between the SNV-based clone phylogenies and the MEDICC2 phylogenies at the cohort level, and when discussing individual tumours, we normalised each root-to-leaf length by the maximum observed root-to-leaf length of the corresponding phylogeny for that tumour. To compare topologies, we used the regular Robinson-Foulds distance (as implemented in the R package ape 5.6.1) to evaluate similarity between the SNV-based clone phylogenies and the MEDICC2 trees. We took the leaves as annotated in “Figure 2: Subclonal structure within 10 metastatic lethal prostate cancers.” from Gundem et al. When multiple samples were listed as a single leaf we introduced a bifurcation at this position e.g. tumour A12 with leaf AC. In addition, when multiple samples were listed in multiple leaves we chose the leaf with each sample’s largest CCF contribution to represent that sample. Finally, the Gundem et al. trees contain multifurcations, which support a variety of possible bifurcating trees. For comparison with a binary hypothesis tree as inferred by MEDICC2, we considered all possible bifurcations obtainable from any given multifurcation, and used the minimum Robinson-Foulds distance across this set of bifurcations to determine a possible match.”

Minor comments

1. Page 6, line 60, "counting genomic segments" is ambiguous, counting the number of copies of each segment?

Authors’ response: The text has been changed to “counting the number of copies of each segment”.

2. Page 8, line 9, "predicted to have undergone two WGD events" … please specify if these events were parallel or consecutive

Authors’ response: We have changed the text to specify “two consecutive WGD events”.

3. Page 8, line 32, did you mean supp figure 4 instead of supp figure 14?

Authors’ response: We have corrected this error.

4. For figure 5, i assume the left is total copy number and the right is minor copy number but i couldn't find labels or info from the figure legend.

Authors’ response: We thank the reviewer for this helpful remark and have added labels to the figure as shown below. Similar changes were made to Supplementary Figure 23.


Figure 5: Inferred phylogeny for single-cell data with 1,023 cells. Inferred phylogeny and allele-specific copy-number profiles for patient TN2 from Minussi et al. 2021. The diploid and most recent common ancestor to all cells are marked with green and blue circles, respectively. We manually selected clades from the phylogeny to match the superclones and subclones of the original publication. These are marked next to the tree in the colours of the original publication and with horizontal lines. The structure of the tree corresponds very clearly with distinct features of the copy-number profiles and matches the clonal structure derived in the original publication. Selected synapomorphies of the clone structure are highlighted with a yellow border and annotated on the figure.


Other changes
Updated Gundem preprocessing
In the previous version of our manuscript we applied reference phasing to each consensus genomic segment across all samples from a single tumour separately. This enabled us to identify SCNAs in the same genomic region that originate from opposite haplotypes revealing mirrored subclonal allelic imbalance and parallel evolution. In this latest version of the manuscript we have also applied a process we term “horizontal phasing” in order to attempt to remove potentially artifactual inferred SCNA transitions introduced by the segment by segment choice of the optimal reference sample for phasing within a chromosome during reference phasing. Horizontal phasing reassigns the heterozygous SNPs assigned to the “A” or “B” haplotype along a chromosome in order to minimise the number of copy-number events between a diploid normal sample and the tumour sample. For further details see Methods section “Bulk data processing and analysis”.

**Second round of review**

**Reviewer 1**

As in my initial review, I still believe this manuscript is well written and a valuable contribution to cancer genomics methodology. In the revision, the authors have thoroughly addressed the comments I made. The rewritten discussion effectively places the algorithm in the context of existing work. The extended simulated analyses, including adding a breakpoint based method, simulations with model violations, and runtime analysis depict a fair and favorable picture of the capabilities of the algorithm.

**Reviewer 2**

The revisions to the manuscript have satisfied my concerns.
